# Acetylshikonin suppressed growth of colorectal tumour tissue and cells by inhibiting the intracellular kinase, T‐lymphokine‐activated killer cell‐originated protein kinase.

**DOI:** 10.1111/bph.14981

**Published:** 2020-04-10

**Authors:** Ran Zhao, Bu Young Choi, Lixiao Wei, Mangaladoss Fredimoses, Fanxiang Yin, Xiaorong Fu, Hanyong Chen, Kangdong Liu, Joydeb Kumar Kundu, Zigang Dong, Mee‐Hyun Lee

**Affiliations:** ^1^ Department of Pathophysiology, School of Basic Medical Sciences Zhengzhou University Zhengzhou China; ^2^ China‐US (Henan) Hormel Cancer Institute Zhengzhou China; ^3^ Department of Pharmaceutical Science and Engineering, School of Convergence Bioscience and Technology Seowon University Chungbuk South Korea; ^4^ The Hormel Institute University of Minnesota Austin Minnesota; ^5^ The Collaborative Innovation Center of Henan Province for Cancer Chemoprevention Zhengzhou China; ^6^ Li Ka Shing Applied Virology Institute University of Alberta Edmonton Alberta Canada

## Abstract

**Background and Purpose:**

Overexpression or aberrant activation of the T‐lymphokine‐activated killer cell‐originated protein kinase (TOPK) promotes gene expression and growth of solid tumours, implying that TOPK would be a rational target in developing novel anticancer drugs. Acetylshikonin, a diterpenoid compound isolated from *Lithospermum erythrorhizon* root, exerts a range of biological activities. Here we have investigated whether acetylshikonin, by acting as an inhibitor of TOPK, can attenuate the proliferation of colorectal cancer cells and the growth of patient‐derived tumours, in vitro and in vivo.

**Experimental Approach:**

Targets of acetylshikonin, were identified using kinase profiling analysis, kinetic/binding assay, and computational docking analysis and knock‐down techniques. Effects of acetylshikonin on colorectal cancer growth and the underlying mechanisms were evaluated in cell proliferation assays, propidium iodide and annexin‐V staining analyses and western blots. Patient‐derived tumour xenografts in mice (PDX) and immunohistochemistry were used to assess anti‐tumour effects of acetylshikonin.

**Key Results:**

Acetylshikonin directly inhibited TOPK activity, interacting with the ATP‐binding pocket of TOPK. Acetylshikonin suppressed cell proliferation by inducing cell cycle arrest at the G1 phase, stimulated apoptosis, and increased the expression of apoptotic biomarkers in colorectal cancer cell lines. Mechanistically, acetylshikonin diminished the phosphorylation and activation of TOPK signalling. Furthermore, acetylshikonin decreased the volume of PDX tumours and reduced the expression of TOPK signalling pathway in xenograft tumours.

**Conclusion and Implications:**

Acetylshikonin suppressed growth of colorectal cancer cells by attenuating TOPK signalling. Targeted inhibition of TOPK by acetylshikonin might be a promising new approach to the treatment of colorectal cancer.

**Abbreviations**
PDX: patient‐derived tumour xenograft
RSK: ribosomal s6 kinase
TOPK: T (T‐LAK)‐cell‐originated protein kinase

**What is already known**

- The intracellular protein kinase, TOPK/PBK, is highly expressed in colorectal cancer cells.

**What this study adds**

- Acetylshikonin inhibited colorectal cancer growth dependent on the expression of TOPK/PBK.

**What is the clinical significance**

- Acetylshikonin could provide a new approach to treatments for colorectal cancer.

## 1 INTRODUCTION

Colorectal cancer is one of the most common causes of cancer‐related death. It comprises a substantial proportion of the global burden of cancer morbidity and mortality, being the fourth most common cause of cancer mortality, accounting for 600,000 deaths annually (Rossi, Anwar, Usman, Keshavarzian, & Bishehsari, 2018). Colorectal cancer is the third most commonly diagnosed cancer among men and the second most diagnosed among women worldwide (Kolligs, 2016; Roswall & Weiderpass, 2015). New cases diagnosed each year in the United States are estimated to be around 95,520 which results in about 50,260 patients deaths (Siegel et al., 2017). Although there have been remarkable progress in developing anticancer therapies including recently approved immune check point inhibitors (Davide et al., 2019), the incidence of and mortality from colorectal cancer is still alarming. Because of the heterogenous nature of colorectal cancer, interventions in a range of oncogenic signalling pathways would be a rational approach for developing new therapies. One of the emerging oncogenic signalling molecules is an intracellular protein kinase, the T‐lymphokine‐activated killer cell‐originated protein kinase (https://www.guidetopharmacology.org/GRAC/FamilyDisplayForward?familyId=633; also known as PDZ binding kinase, PBK), which has been implicated in the development and progression of gastric (Ohashi et al., 2017) and ovarian cancers (Ikeda et al., 2016), oesophageal squamous cell carcinomas (Ohashi et al., 2016), and colorectal cancer (Zlobec et al., 2010).

https://www.guidetopharmacology.org/GRAC/FamilyDisplayForward?familyId=633 is a PDZ‐binding kinase (PBK) and a serine–threonine kinase of the MAPK kinase (MAPKK) family (Abe, Matsumoto, Kito, & Ueda, 2000). TOPK is a member of the novel MEK3/6‐related MAPKK family (Aksamitiene, Kholodenko, Kolch, Hoek, & Kiyatkin, 2010) and plays an important role in many kinds of cellular processes, including growth, development, apoptosis, and inflammation (Li et al., 2011; Nandi, 2004; Simons‐Evelyn et al., 2001; Zykova et al., 2010). Elevated expression of TOPK is associated with poor prognosis of ovarian cancer (Ikeda et al., 2016), oesophageal squamous carcinoma (Ohashi et al., 2016), and gastric carcinoma patients (Ohashi et al., 2017). TOPK directly phosphorylates https://www.guidetopharmacology.org/GRAC/FamilyDisplayForward?familyId=514, histone H3 (Ser10), histone H2AX (Ser139), peroxiredoxin 1 (PRX1, Ser32), https://www.guidetopharmacology.org/GRAC/ObjectDisplayForward?objectId=1496 (Thr183/Tyr185), and p53‐related protein kinase (PRPK, Ser250; Oh et al., 2007; Roh et al., 2018; Zykova et al., 2010). These phosphorylated substrates of TOPK activate down‐stream signalling cascades, including the MAPKs, and ribosomal S‐6 kinase (https://www.guidetopharmacology.org/GRAC/FamilyDisplayForward?familyId=541), and transcription factors, including activator protein‐1 (AP‐1) or NF‐κB, thereby promoting cell proliferation, migration, and invasion (Aksamitiene et al., 2010; Oh et al., 2007; Park et al., 2014). TOPK is highly expressed in human colorectal cancer tissues and cell lines and mediates its tumour promoting effects via phosphorylation of ERKs (Zhu et al., 2007). Zhu et al. (2007) also demonstrated that phosphorylation of TOPK at Tyr74 and Tyr272 by https://www.guidetopharmacology.org/GRAC/ObjectDisplayForward?objectId=2206&familyId=619&familyType=ENZYME increases the stability and activity of TOPK, thereby promoting colon cancer growth (Xiao et al., 2016). The TOPK signalling pathway directly promotes colorectal cancer metastasis through its modulation of PRPK (Zykova et al., 2017; Zykova et al., 2018). TOPK also interacts with the DNA binding domain of p53 tumour suppressor protein, thereby down‐regulating p53‐regulated gene transcription (Hu et al., 2010; Lei et al., 2013). Thus, TOPK is a potential drug target for developing new therapies for the treatment of colorectal cancer.

There have been few reports of synthesis and functional studies of TOPK inhibitors, such as HI‐TOPK‐032 (Kim et al., 2012) and https://www.guidetopharmacology.org/GRAC/LigandDisplayForward?ligandId=7813 (Sugimori et al., 2017). Whereas HI‐TOPK‐032 reduced the growth of colon cancer xenograft tumours in mice (Kim et al., 2012), OTS964 inhibited the size of glioma stem cells tumour spheres in vitro (Sugimori et al., 2017). However, the latter study also demonstrated that the surviving glioma stem cells start to regrow as tumour spheres, thus limiting the therapeutic value of OTS964. Moreover, OTS964 showed substantial adverse haematological reactions in an in vivo xenograft study (Matsuo et al., 2014). Hu et al. (2019) recently reported the synthesis of a series of 1‐phenyl phenanthridin‐6(5*H*)‐one compounds as TOPK inhibitors, which reduced the growth of colorectal tumour growth in a xenograft mouse model. These studies suggest that TOPK is a valid drug target for developing anticancer therapies.

In the present study, we have attempted to evaluate the potential of developing acetylshikonin, a major biologically active compound present in *Lithospermum erythrorhizon* root (Cho, Paik, & Hahn, 1999; Rajasekar et al., 2012), as a TOPK inhibitor and assess its anti‐cancer effects in cultureds of colorectal cancer cells and in patient‐derived xenograft (PDX) tumour models in mice. Acetylshikonin reportedly inhibits human pancreatic cancer cell proliferation through inhibition of NF‐κB activity, attenuates HepG2 hepatoma cell growth by suppressing https://www.guidetopharmacology.org/GRAC/FamilyDisplayForward?familyId=262#1332, and reduces obesity and hepatic steatosis in db/db mice (Cho & Choi, 2015; Gwon, Ahn, Chung, Moon, & Ha, 2012; Park et al., 2017). These findings led us to examine whether acetylshikionin could inhibit colorectal tumour growth and to elucidate its underlying mechanisms. Here, we report that acetylshikonin directly interacts with TOPK and inhibits TOPK kinase activity, resulting in reduced proliferation of colon cancer cells and diminished growth of PDX tumours in mice. Our study suggests that acetylshikonin, as an inhibitor of TOPK, may be a potential candidate for clinical development as an anticancer therapy for colorectal cancer.

## 2 METHODS

### 2.1 Cell culture

The human CCD‐18Co (Cat#CRL‐1459, RRID:CVCL_2379), normal colon cell line and colorectal cancer cell lines HCT‐15 (Cat#CCL‐225, RRID:CVCL_0292), HCT‐116 (Cat#CCL‐247, RRID:CVCL_0291), SW 620 (Cat#CCL‐227, RRID:CVCL_0547), DLD‐1 (Cat#CCL‐221, RRID:CVCL_0248), HT‐29 (Cat#HTB‐38, RRID:CVCL_0320), and SW 480 (Cat#CCL‐228, RRID:CVCL_0546) were purchased from American Type Culture Collection (ATCC). The cells were cultured in a minimum essential medium (CCD‐18Co), RPMI‐1640 (HCT‐15, DLD‐1), McCoy's 5A (HCT‐116, HT‐29), or L‐15 (SW 620, SW 480) containing penicillin (100 units·ml^−1^), streptomycin (100 μg·ml^−1^), and 10% FBS (Biological Industries, Kibbutz Beit‐Haemek, Israel). The cells were maintained at 5% CO_2_, 37°C in a humidified atmosphere. All cells were cytogenetically tested and authenticated before being frozen. Each vial of frozen cells was thawed and maintained in culture for a maximum of 8 weeks.

### 2.2 In vitro kinase assay

c‐Jun (400 ng), ERK1 (200 ng), and histone H3.3 (200 ng) were used as substrates for in vitro kinase assays with 200 ng of active TOPK, MEK1, https://www.guidetopharmacology.org/GRAC/ObjectDisplayForward?objectId=1936 and https://www.guidetopharmacology.org/GRAC/ObjectDisplayForward?objectId=1937&familyId=557&familyType=ENZYME. Reactions were conducted at 30°C for 30 min in 1× kinase buffer (25‐mM Tris–HCl, pH 7.5, 5‐mM β‐glycerophosphate, 2‐mM DTT, 0.1‐mM Na_3_VO_4_, 10‐mM MgCl_2_, and 5‐mM MnCl_2_) containing 200‐μM ATP. The reactions were stopped, and the proteins were detected by western blotting.

### 2.3 In vitro and ex vivo pull‐down assay

A recombinant human TOPK protein (200 ng) and SW 620 cell lysates (500 μg) were incubated with acethylshikonin‐Sepharose 4B (or Sepharose 4B only as a control) beads in reaction buffer (50‐mM Tris–HCl, pH 7.5, 5‐mM EDTA, 150‐mM NaCl, 1‐mM DTT, 0.01% NP‐40, and 0.2‐mM PMSF, 20× protease inhibitor). After incubation with gentle rocking overnight at 4°C, the beads were washed three times with washing buffer (50‐mM Tris–HCl, pH 7.5, 5‐mM EDTA, 150‐mM NaCl, 1‐mM DTT, 0.01% NP‐40, and 0.2‐mM PMSF), and binding was visualized by western blotting. For the ATP competitive binding assay, active TOPK was incubated with acetylshikonin‐Sepharose 4B beads and vehicle, 10‐, 100‐, or 1,000‐μM ATP, following the procedure described above for the binding assay.

### 2.4 Computational modelling

To further confirm that acetylshikonin can bind with TOPK, we performed in silico docking using the Schrödinger Suite 2017 software programs (Schrödinger, 2017). The sequence of TOPK was downloaded from the National Center for Biotechnology Information (GI: 83305809). The TOPK crystal structure was built with prime followed by refining and minimizing loops in the binding site, and then it was prepared under the standard procedures of the Protein Preparation Wizard. Hydrogen atoms were added consistent with a pH of 7, and all water molecules were removed. The TOPK ATP‐binding site‐based receptor grid was generated for docking. Acetylshikonin was prepared for docking by default parameters using the LigPrep program. Then, the docking of acetylshikonin with TOPK was accomplished with default parameters under the extra precision (XP) mode using the program Glide. Then, TOPK modelling structure was refining and minimizing loops in the binding site. When the docking was performed, usually several docking models were generated. Herein, we based on the docking score and ligand interaction diagram to choosing best docked representative structure for our final docking model.

### 2.5 Cell proliferation assay

The cells were seeded (2 × 10^3^ cells per well for HCT‐116 and HCT‐15, 4 × 10^3^ cells per well for SW 620, and 1 × 10^3^ cells per well for DLD‐1) in 96‐well plates and incubated for 36 hr and then treated with different doses of acetylshikonin or vehicle. After incubation for 24, 48, or 72 hr, cell proliferation was measured by MTT assay. For anchorage‐independent cell growth assessment, the cells (8 × 10^3^ per well) suspended in 10% maintenance media were added to 0.3% agar with vehicle, 0.3‐, 0.6‐, or 1.25‐μM acetylshikonin, in a top layer over a base layer of 0.5% agar with vehicle, 0.3‐, 0.6‐, or 1.25‐μM acetylshikonin. The cultures were maintained at 37°C in a 5% CO_2_ incubator for 3 weeks, and then colonies were visualized under a microscope and counted using the Image‐Pro Plus software program (Media Cybernetics, Rockville, MD, USA, Version 6.0, RRID:SCR_007369).

### 2.6 Analysis of cell cycle and apoptosis

The cells (2 × 10^5^ cells) were seeded in 60‐mm dishes and treated with 0‐, 5‐, or 10‐μM acetylshikonin for 24 hr. For cell cycle analysis, the cells were fixed in 70% ethanol and stored at −20°C for 24 hr. After staining with annexin‐V for apoptosis or propidium iodide for cell cycle assessment, the cells were analysed using a BD FACSCalibur Flow Cytometer (BD Biosciences, San Jose, CA, USA).

### 2.7 Western blot analysis

Cell pellets were incubated on ice for 30 min in NP‐40 cell lysis buffer (50‐mM Tris, pH 8.0, 150‐mM NaCl, 0.5–1% NP‐40, protease inhibitor cocktail, dephosphorylation inhibitor tablets, and 1‐mM PMSF). After centrifugation at 18,000 x *g* for 20 min, the supernatant fractions were harvested as total cellular protein extracts. Determination of protein concentration was performed using the BCA Quantification Kit (Solarbio, Beijing, China, Cat#PC0020). The total cellular protein extracts were separated by SDS‐PAGE and transferred to PVDF membranes in a transfer buffer. Membranes were blocked with 5% nonfat‐dry milk in 1× PBS‐T (PBS containing 0.05% Tween‐20) and incubated with antibodies against pTOPK, TOPK, pMEK, MEK, pERK, ERK, pRSK, RSK, pc‐Jun, c‐Jun, p21, PARP, caspase‐3, cleaved PARP, cleaved caspase‐3, cleaved caspase‐7, or β‐actin. Blots were washed three times in 1× PBS‐T buffer, followed by incubation with the appropriate HRP‐linked IgG. The specific proteins were visualized using the enhanced chemiluminescene (ECL) detection reagent and the Amersham Imager 600 (GE Healthcare Life Science, Pittsburgh, PA, USA). Quantification was done with ImageJ free software (Version 1.47, RRID:SCR_003070), and each lane was normalized to β‐actin. The immuno‐related procedures used comply with the recommendations made by the *British Journal of Pharmacology* (Curtis et al., 2015).

### 2.8 Lentiviral infection

Each viral vector and packaging vectors (*pMD2G, psPAX2, shTOPK #1–4*) were transfected using the Simple‐Fect Transfection Reagent (Signaling Dawn Biotech, Wuhan, Hubei, China, Cat#profect‐01) into 293T cells. The viral particles were harvested by filtration using a 0.22‐μm filter and then stored at −20°C. The cultured HCT‐15, HCT‐116, SW 620, or DLD‐1 colon cancer cells were infected with virus particles and 8 μg·ml^−1^ polybrene (Millipore, Billerica, MA, USA, Cat#TR‐1003) for 24 hr. Then, the cells were selected with puromycin for 24 hr, and the selected cells were used for subsequent experiments. The cells of DLD‐1 were seeded (1 × 10^3^ cells per well) in 96‐well plates, and after incubation for 24, 48, or 72 hr, cell proliferation was measured by CellTiter 96® Aqueous One Solution Cell Proliferation Assay (MTS assay, Promega, G3581); the protocol is followed by CellTiter 96® Aqueous One Solution Cell Proliferation Assay Technical Bulletin.

### 2.9 Reporter gene activity assay

Transient transfection was conducted using the Simple‐Fect Transfection Reagent, and the luciferase reporter gene activity assays were performed according to the instructions of the manufacturer (Promega, Madison, WI, USA). Briefly, the cells (2 × 10^4^) were seeded the day before transfection into 24‐well culture plates. The cells were co‐transfected with the *NF‐κΒ‐luc* or *pGL‐3‐luc* firefly reporter plasmid and the *pRL‐SV* control *Renilla* reporter plasmid. Following incubation for 24 hr, the cells were treated with vehicle or acetylshikonin for an additional 24 hr and then harvested in a lysis buffer. Firefly and *Renilla* luciferase activities were assessed using the substrates provided in the reporter assay system (Promega, Cat#E2810). The *Renilla* luciferase activity was used for normalization of transfection efficiency. Luciferase activity was measured using the Luminoskan Ascent plate reader (Thermo‐Scientific, Swedesboro, NJ, USA).

### 2.10 PDX mouse model

All animal care and experimental procedures complied with, and were approved by, the Ethics Committee of Zhengzhou University (Zhengzhou, Henan, China). Six‐ to eight‐week‐old female NOD.CB17‐Prkdcscid/NcrCrl mice (Vital River Labs, Beijing, China) with body weight 18–20 g were used for these experiments. Animals were housed in a specific pathogen‐free facility using IVC level cages, bedding material made of corncob granules, housing five mice per cage. The mice were maintained on a 12‐hr light/dark cycle with food and water available ad libitum, at a temperature of 22 ± 3°C and 40–70% relative humidity. Animal studies are reported in compliance with the ARRIVE guidelines (Kilkenny *et al*., 2010) and with the recommendations made by the *British Journal of Pharmacology*.

The PDX mouse model has been widely used in preclinical studies to identify therapeutic targets, including specific molecules and molecular interactions, and to serve as a guide for clinical treatment of cancer (Lai et al., 2017). The PDX tumour samples were obtained from three different patients (HJG41, HJG175, and HJG152; see Figure S5B for details) with permission from the Ethical Committee of China‐US (Henan) Hormel Cancer Institute and with full informed consent of the patients. The PDX tumour mass (100‐130 mg per mouse) was subcutaneously implanted into the back of SCID mice. When tumours reached an average volume of ~100 mm^3^, mice were divided into three treatment groups by randomization and blinding methods for further experimentation as follows: for HJG41 and HJG175 cases, vehicle group (*n* = 5 for HJG41, *n* = 8 for HJG152); (2) 60 mg·kg^−1^ of acetylshikonin (*n* = 5 for HJG41, *n* = 8 for HJG152); and (3) 120 mg·kg^−1^ of acetylshikonin (*n* = 5 for HJG41, *n* = 8 for HJG152). For HJG 175 cases, vehicle group (*n* = 10); (2) 80 mg·kg^−1^ of acetylshikonin (*n* = 10); and (3) 160 mg·kg^−1^ of acetylshikonin (*n* = 10). Tumour volume was calculated from measurements of three diameters of the individual tumour base using the following formula: tumour volume (mm^3^) = (length × width × height × 0.5). Acetylshikonin was administered by gavage once a day until tumours reached ~1.0 cm^3^ total volume; at which time mice were killed by an overdose of pentobarbital (4%, i.p.), before tumour collection.

### 2.11 Immunohistochemical (IHC) analysis

Subcutaneous tumours were collected from the mice and fixed, and paraffin‐embedded sections (5 μm) were prepared for IHC analysis. After antigen unmasking, the sections were blocked with 5% goat serum and incubated at 4°C overnight with antibodies rabbit anti‐Ki‐67 (1:200, Abcam, Cat#ab16667), rabbit anti‐phosphorylated TOPK (pTOPK) (1:200, Sigma, Cat#SAB4504053, RRID:AB_2070042), rabbit anti‐phosphorylated ERK1/2 (Thr202/Tyr204) (pERK) (1:100), rabbit anti‐phosphorylated RSK (Ser380) (pRSK) (1:100), and rabbit anti‐phosphorylated c‐Jun (pc‐Jun) (1:100). After incubation with a rabbit secondary antibody, DAB (3,3′‐diaminobenzidine) staining was used following the manufacturer's instructions to visualize the protein targets. Sectioned tissues were counterstained with haematoxylin, dehydrated through a graded series of alcohol into xylene, and mounted under glass coverslips. Images were obtained using OLYMPUS IMAGING BX43. The fluorescence intensity was quantified using Image‐Pro Plus software (Version 6.0, RRID:SCR_007369).

### 2.12 Data and statistical analysis

The design and analysis of this study complies with the recommendations of the *British Journal of Pharmacology* on experimental design (Curtis et al., 2018). Animals were randomly allocated to the experimental groups. Data collection and evaluation of all experiments were performed blindly of the group identity. Statistical analysis was carried out using the software GraphPad Prism (v7, GraphPad Software, USA, RRID:SCR_002798). Differences among multiple groups were tested using one‐way ANOVA followed by Dunnett's post hoc comparisons. Post hoc tests were conducted only if *F* was significant and there was no variance inhomogeneity. A value of *P* < .05 was used as the criterion for statistical significance, and the data are shown as mean values ± *SD.*

### 2.13 Materials

Acetylshikonin (purity >95%) was purchased from Chem Faces (Wuhan, Hubei, China) and isolated in‐house from *L. erythrorhizon* root for animal experiments. Active TOPK (Cat#T14‐10G‐10), MEK1 (Cat#M02‐10G‐10), Aurora A (Cat#A28‐18G‐10), Aurora B (Cat#A33‐10G‐10), inactive c‐Jun protein (TOPK substrate, Cat#J05‐55G‐20), and inactive ERK1 protein (MEK1 substrate, Cat#M29‐14G‐20) for kinase assays were purchased from SignalChem Biotech Inc. (Richmond, BC, Canada). The histone H3.3 human recombinant protein (substrate of Aurora A and Aurora B, Cat#M2507S) for kinase assays was purchased from New England Biolabs, Inc. (Ipswich, MA, USA). c‐Src kinase assay was purchased from Cyclex (Andes, NY, USA, Cat#CY‐1083). Antibodies used in this study were as follows: rabbit anti‐phosphorylated TOPK (Thr9) (pTOPK) (1:1,000, Cell Signaling Technology, Cat#4941, https://www.guidetopharmacology.org/GRAC/ObjectDisplayForward?objectId=1994, rabbit anti‐TOPK (1:1,000, Cell Signaling Technology, Cat#4942, https://www.guidetopharmacology.org/GRAC/ObjectDisplayForward?objectId=1994, rabbit anti‐phosphorylated MEK (Ser217/221) (pMEK) (1:1,000, Cell Signaling Technology, Cat#9154, https://www.guidetopharmacology.org/GRAC/ObjectDisplayForward?objectId=1994, mouse anti‐MEK (1:1,000, Cell Signaling Technology, Cat#4694, https://www.guidetopharmacology.org/GRAC/ObjectDisplayForward?objectId=1994, rabbit anti‐phosphorylated ERK (Thr202/Tyr204) (pERK) (1:1,000, Cell Signaling Technology, Cat#4370, https://www.guidetopharmacology.org/GRAC/ObjectDisplayForward?objectId=1994, rabbit anti‐ERK (1:1,000, Cell Signaling Technology, Cat#4695, https://www.guidetopharmacology.org/GRAC/ObjectDisplayForward?objectId=1994, rabbit anti‐phosphorylated RSK (Ser380) (pRSK) (1:1,000, Cell Signaling Technology, Cat#11989, https://www.guidetopharmacology.org/GRAC/ObjectDisplayForward?objectId=1994, rabbit anti‐RSK (1:1,000, Cell Signaling Technology, Cat#5528, https://www.guidetopharmacology.org/GRAC/ObjectDisplayForward?objectId=1994, rabbit anti‐phosphorylated c‐Jun (pc‐Jun) (1:1,000, Cell Signaling Technology, Cat#3270, https://www.guidetopharmacology.org/GRAC/ObjectDisplayForward?objectId=1994, rabbit anti‐c‐Jun (1:1,000, Cell Signaling Technology, Cat#9165, https://www.guidetopharmacology.org/GRAC/ObjectDisplayForward?objectId=1994, rabbit anti‐cyclin D1 (1:1,000, Cell Signaling Technology, Cat#2922, https://www.guidetopharmacology.org/GRAC/ObjectDisplayForward?objectId=1994, mouse anti‐cyclin D3 (1:1,000, Cell Signaling Technology, Cat#2936, https://www.guidetopharmacology.org/GRAC/ObjectDisplayForward?objectId=1994, rabbit anti‐p21 waf1/cip1 (1:1,000, Cell Signaling Technology, Cat#2947, https://www.guidetopharmacology.org/GRAC/ObjectDisplayForward?objectId=1994, rabbit anti‐PARP (1:1,000, Cell Signaling Technology, Cat#9542, https://www.guidetopharmacology.org/GRAC/ObjectDisplayForward?objectId=1994, rabbit anti‐caspase‐3 (1:1,000, Cell Signaling Technology, Cat#9662, https://www.guidetopharmacology.org/GRAC/ObjectDisplayForward?objectId=1994, rabbit anti‐cleaved‐PARP (1:1,000, Cell Signaling Technology, Cat#5625, https://www.guidetopharmacology.org/GRAC/ObjectDisplayForward?objectId=1994, rabbit anti‐cleaved caspase‐3 (1:1,000, Cell Signaling Technology, Cat#9664, https://www.guidetopharmacology.org/GRAC/ObjectDisplayForward?objectId=1994 and rabbit anti‐cleaved caspase‐7 (1:1,000, Cell Signaling Technology, Cat#8438, https://www.guidetopharmacology.org/GRAC/ObjectDisplayForward?objectId=1994 by western blot were purchased from Cell Signaling Technology (Beverly, MA, USA). Rabbit anti‐phosphorylated TOPK (pTOPK) (1:200, Sigma, Cat#SAB4504053, https://www.guidetopharmacology.org/GRAC/ObjectDisplayForward?objectId=1994 to detect immunohistochemical was purchased from Sigma. Antibodies rabbit anti‐Ki‐67 (1:200, Abcam, Cat#ab16667, https://www.guidetopharmacology.org/GRAC/ObjectDisplayForward?objectId=1994 to detect Ki‐67 by immunohistochemical assay was purchased from Sigma. The mouse anti‐β‐actin antibody (1:5,000, Cat#TA‐09) as a loading control was obtained from ZSGB‐Bio Company (Beijing, China). Goat anti‐rabbit antibody (1:5,000, Cat#ZB‐2301) and goat anti‐mouse antibody (1:5,000, Cat#ZB‐2305) were obtained from ZSGB‐Bio Company (Beijing, China).

### 2.14 Nomenclature of targets and ligands

Key protein targets and ligands in this article are hyperlinked to corresponding entries in http://www.guidetopharmacology.org/, the common portal for data from the IUPHAR/BPS Guide to PHARMACOLOGY (Harding et al., 2018), and are permanently archived in the Concise Guide to PHARMACOLOGY 2019/20 (Alexander, Fabbro et al., 2019; Alexander, Kelly et al., 2019).

## 3 RESULTS

### 3.1 Acetylshikonin is a novel inhibitor of TOPK but has no effect on MEK1

**FIGURE 1:**
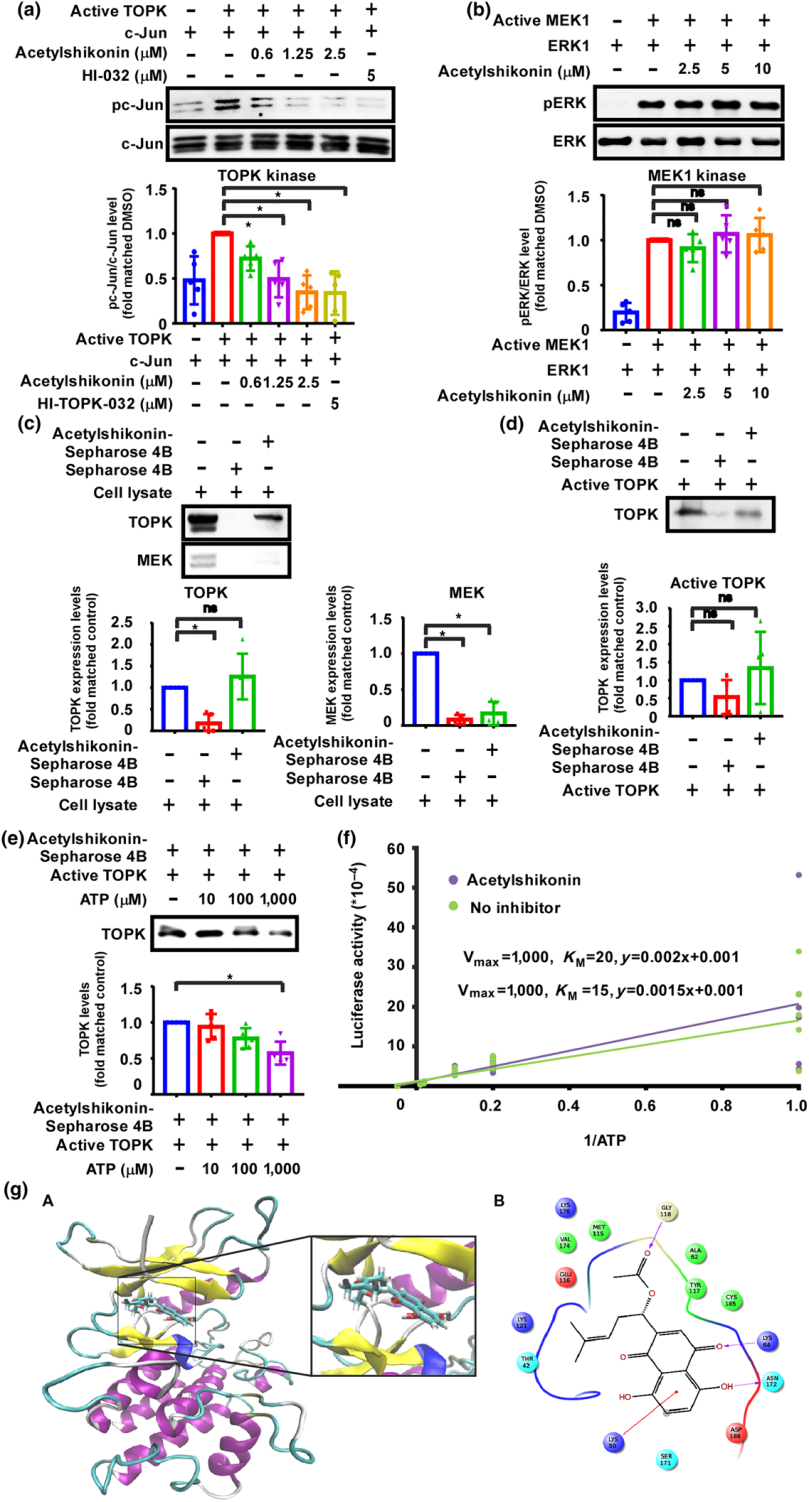
TOPK is a potential target of acetylshikonin. (a) in vitro kinase assay to check the effect of acetylshikonin on TOPK activity, and densitometric quantification was evaluated. Data shown are individual values with means ± SD; n=5 independent experiments. *P < .05, significantly different as indicated. (b) *in vitro* kinase assay to check the effect of acetylshikonin on MEK1 activity, and densitometric quantification was evaluated. Data shown are individual values with means ± SD; n=5 independent experiments. ns, no significant difference as indicated. (c) The binding of acetylshikonin with TOPK and MEK present in SW 620 cell lysate was determined using Sepharose 4B and acetylshikonin‐conjugated Sepharose 4B beads, and densitometric quantification was evaluated. Data shown are individual values with means ± SD; n=5 independent experiments. *P < .05, significantly different as indicated; ns, no significant difference as indicated. (d) The binding of acetylshikonin with a recombinant TOPK protein was determined using Sepharose 4B and acetylshikonin‐conjugated Sepharose 4B beads, and densitometric quantification was performed. Data shown are individual values with means ± SD; n=5 independent experiments. ns, no significant difference as indicated. (e) The specificity of the binding of acetylshikonin with active TOPK in the presence of ATP and densitometric quantification of recombinant TOPK protein were evaluated. Data shown are individual values with means± SD; n=5 independent experiments. *P < .05, significantly different as indicated. (f) Kinetic analysis of the inhibition of TOPK by acetylshikonin competing with ATP. Data shown are individual values; n=5 independent experiments. (g) The interaction between acetylshikonin and TOPK was predicted using a computational docking model: (A) Acetylshikonin binding at the ATP‐binding pocket of TOPK. TOPK structure is shown in ribbon representation, and acetylshikonin is shown as sticks. (B) The ligand interaction diagram of acetylshikonin with TOPK

To examine the effects of acetylshikonin on TOPK activity, we conducted a TOPK kinase assay. Acetylshikonin inhibited the kinase activity of TOPK but had no effect on the kinase activity of https://www.guidetopharmacology.org/GRAC/ObjectDisplayForward?objectId=2062, which is another subfamily of the MAPKKs (Figure 1a,b). The direct binding between acetylshikonin and TOPK was revealed by ex vivo and in vitro pull‐down assays (Figure 1c,d). Acetylshikonin was incubated with SW 620 cell lysate ex vivo or with a human recombinant active TOPK protein in vitro. To confirm whether TOPK is a major target for acetylshikonin, we performed a kinase profiling assay. Kinase profiling assay results showed that acetylshikonin (20 μM) inhibited the kinase activity of Aurora A, Aurora B, and c‐Src but had no effect on the kinase activity of PDK1, Akt1, JNK1, or ERK1 (Figure S1A). We then conducted kinase assays to further confirm the inhibitory effects of acetylshikonin against Aurora A, Aurora B, or c‐Src activity (Figure S1B–D). The kinase assay results showed that acetylshikonin had little effect on the activity of these kinases. To determine whether acetylshikonin can bind to TOPK, we performed an ATP competitive pull‐down assay using a recombinant active TOPK protein. The binding of acetylshikonin with TOPK in the presence of ATP (10, 100, or 1,000 μM) was reduced as ATP concentration was increased (Figure 1e), indicating that acetylshikonin might interact with the ATP‐binding pocket of TOPK. Furthermore, we confirmed that the competitive nature of acetylshikonin and ATP binding to TOPK, by kinetic analysis with an increasing *K*_M_ value 15 to 20 (Figure 1f). To have better insight into how does acetylshikonin interacts with TOPK, we docked it computationally at the ATP‐binding pocket of TOPK, using several programs in the Schrödinger Suite 2017 (Figure 1g). From the docking model, we found that acetylshikonin binds and fits into the ATP‐binding pocket of TOPK very well forming hydrogen bonds at Lys64, Gly118, and Asn172. These results indicated that acetylshikonin might be a potential inhibitor of TOPK.

### 3.2 TOPK as a potential target of acetylshikonin in suppressing colon cancer cell growth

**FIGURE 2:**
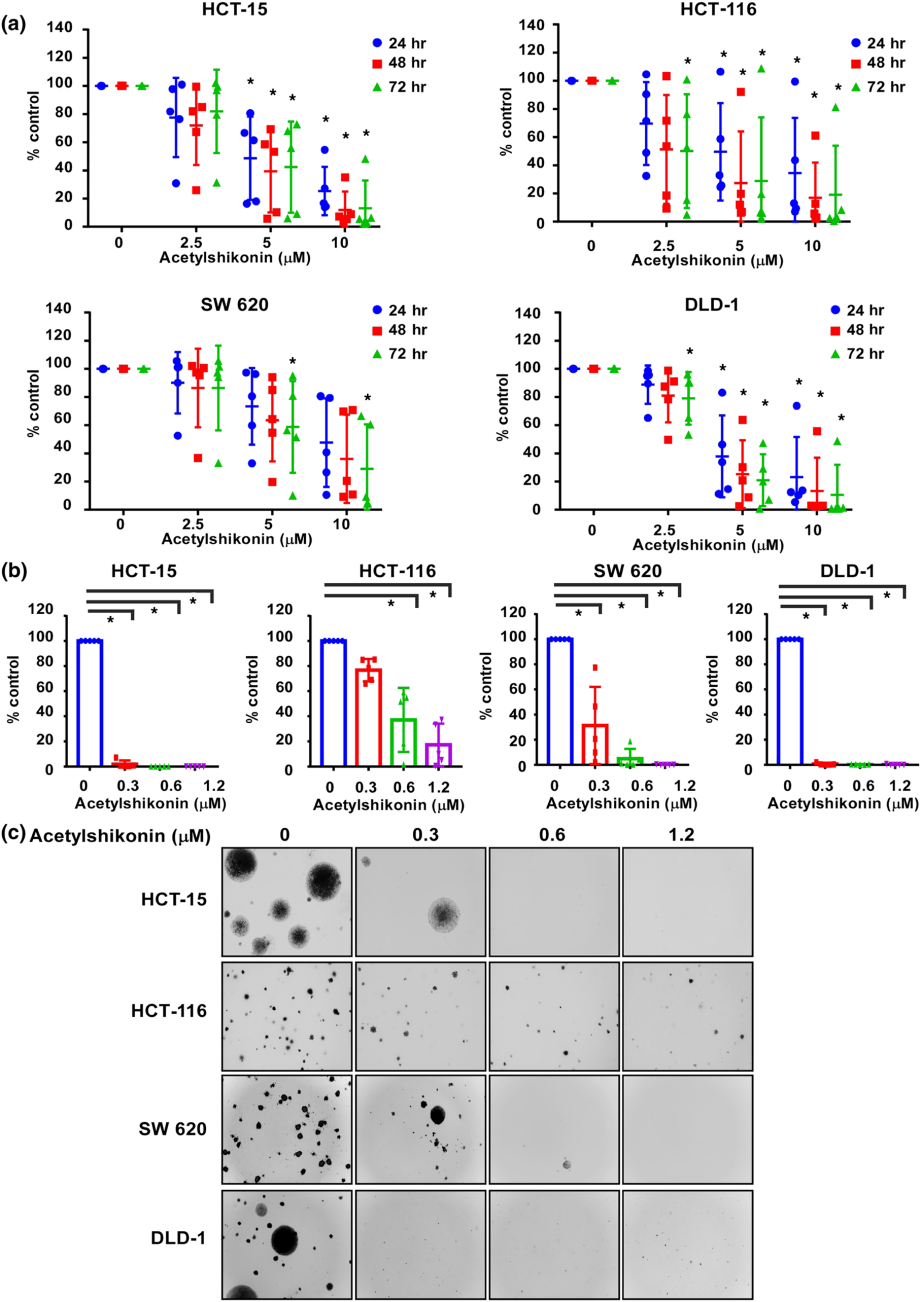
TOPK inhibits proliferation of several colon cancer cell lines. (a) The effect of acetylshikonin on growth of HCT‐15, HCT‐116, SW 620, and DLD‐1 cells was measured by MTT assay at 24, 48, or 72 hr. Data are shown as means ± *SD* of five independent experiments. (b) The effect of acetylshikonin on anchorage‐independent growth of colon cancer cells was evaluated. Data are shown as means ± *SD* of five independent experiments. (c) Representative photographs of the effects of acetylshikonin on anchorage‐independent growth are shown. For (a) and (b), *P < .05, significantly different from untreated control cells

An ideal anti‐cancer agent should lack cytotoxicity against normal cells. We, therefore, first assessed the effect of acetylshikonin on the proliferation of normal human colon cells. Incubation of CCD‐18Co colon cells with acetylshikonin (0, 2.5, 5, or 10 μM) for 24, 48, or 72 hr did not show cytotoxicity up to 10 μM at 72 hr (Figure S2A). Based on this result, we selected the highest concentration of acetylshikonin as 10 μM for further experiments. Because the initial assessment of the expression of pTOPK, TOPK, and the members of TOPK signalling pathway, such as pERK, pRSK, and pc‐Jun, was elevated in HCT‐15, HCT‐116, SW 620, and DLD‐1 colon cancer cells, compared to HT‐29 and SW 480 colon cancer cells (Figure S2B), we studied the effects of acetylshikonin on HCT‐15, HCT‐116, SW 620, and DLD‐1 colon cancer cell lines. Treatment of these cells with acetylshikonin (0, 2.5, 5, or 10 μM) significantly inhibited growth in a time‐ and concentration‐dependent manner compared to DMSO control (Figure 2a). Acetylshikonin showed less inhibition of cell proliferation in HT‐29 and SW 480 cells, compared to the other cell lines (Figure S2C). In addition, acetylshikonin attenuated anchorage‐independent growth of HCT‐15, HCT‐116, SW 620, and DLD‐1 cell lines (0.3, 0.6, or 1.25 μM) compared to the untreated DMSO control (Figure 2b). Representative images illustrate the number and size of colonies (Figure 2c).

### 3.3 Acetylshikonin induces cell cycle arrest and apoptosis of colon cancer cells

**FIGURE 3:**
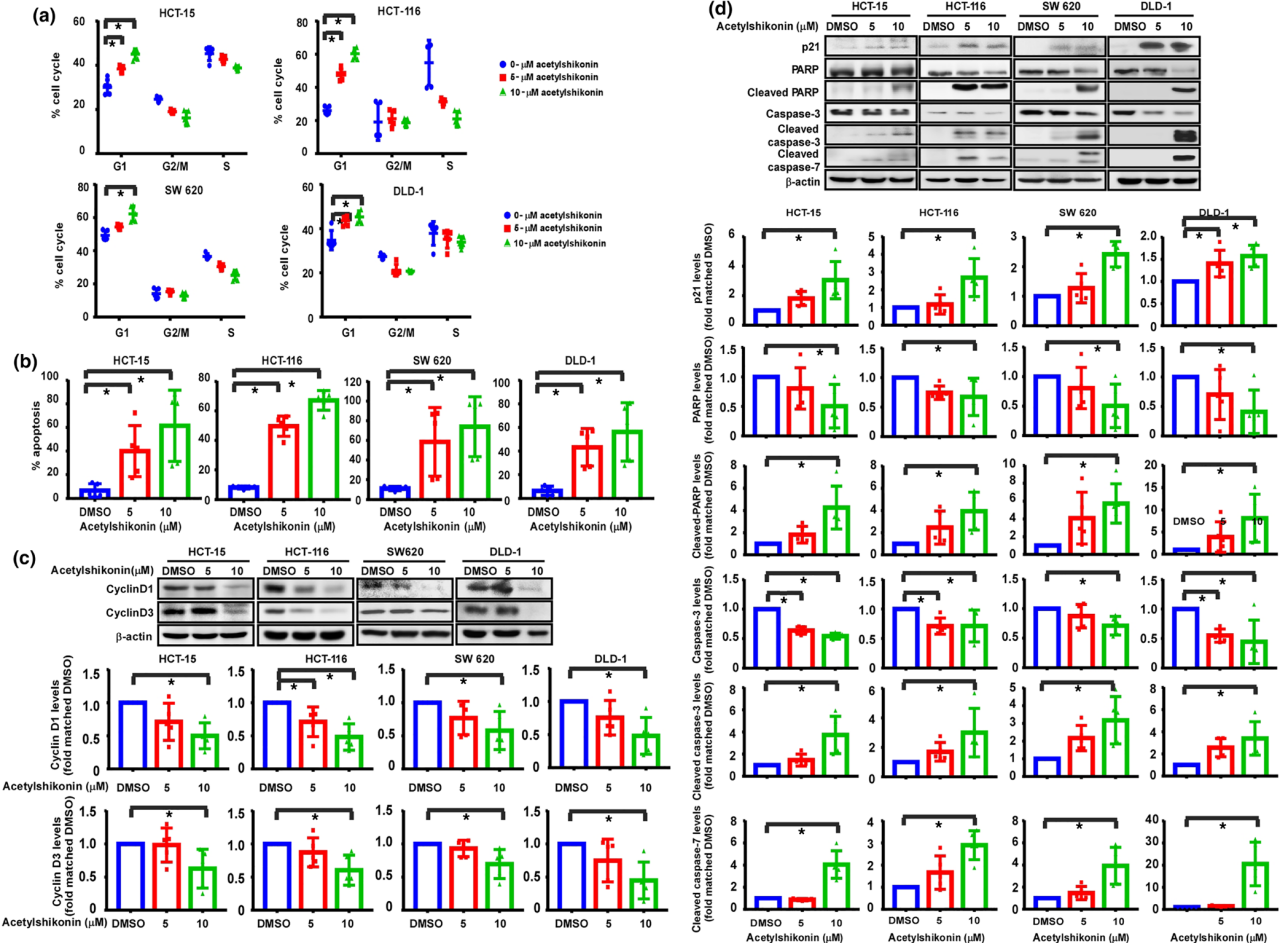
Acetylshikonin induces cell cycle arrest and apoptosis of colon cancer cells. The effects of acetylshikonin on cell cycle phase (a) or apoptosis (b) were assessed in colon cancer cells. The cells were treated with 0‐, 5‐, or 10‐μM acetylshikonin and then incubated 24 hr for cell cycle analysis or for the annexin‐V staining (i.e., apoptosis) assay. Data shown are individual values with means± SD; n=5 independent experiments. *P < .05, significantly different as indicated. The effects of acetylshikonin on the expression of biomarkers and densitometric quantification associated with cell cycle (c) and apoptosis (d) are shown. The cells were treated with 0‐, 5‐, or 10‐μM acetylshikonin and incubated 24 hr for detection of cyclin D1, cyclin D3, p21, cleaved PARP, caspase‐3, cleaved caspase‐3, and cleaved caspase‐7. Data shown are individual values with means± SD; n=5 independent experiments.*P < .05, significantly different as indicated

Treatment with acetylshikonin affected cell cycle distribution and induced apoptosis in colon cancer cell lines (Figure 3). Acetylshikonin (5 or 10 μM) showed significant G1 phase cell cycle arrest in HCT‐15, HCT‐116, SW 620, and DLD‐1 cells (Figure 3a). We further examined how acetylshikonin altered the expression of proteins associated with the G1 phase of the cell cycle (Figure 3b). Acetylshikonin reduced the expression of cyclin D1 and cyclin D3 as compared with the DMSO control (Figure 3b). Acetylshikonin (5 or 10 μM) also induced apoptosis in these cell lines (Figure 3c). Treatment of colon cancer cells with acetylshikonin‐increased levels of p21, cleaved PARP, cleaved caspase‐3, and cleaved caspase‐7, and decreased the total levels of PARP, and caspase‐3 as compared to DMSO controls (Figure 3d).

### 3.4 Acetylshikonin inhibits TOPK signalling

**FIGURE 4:**
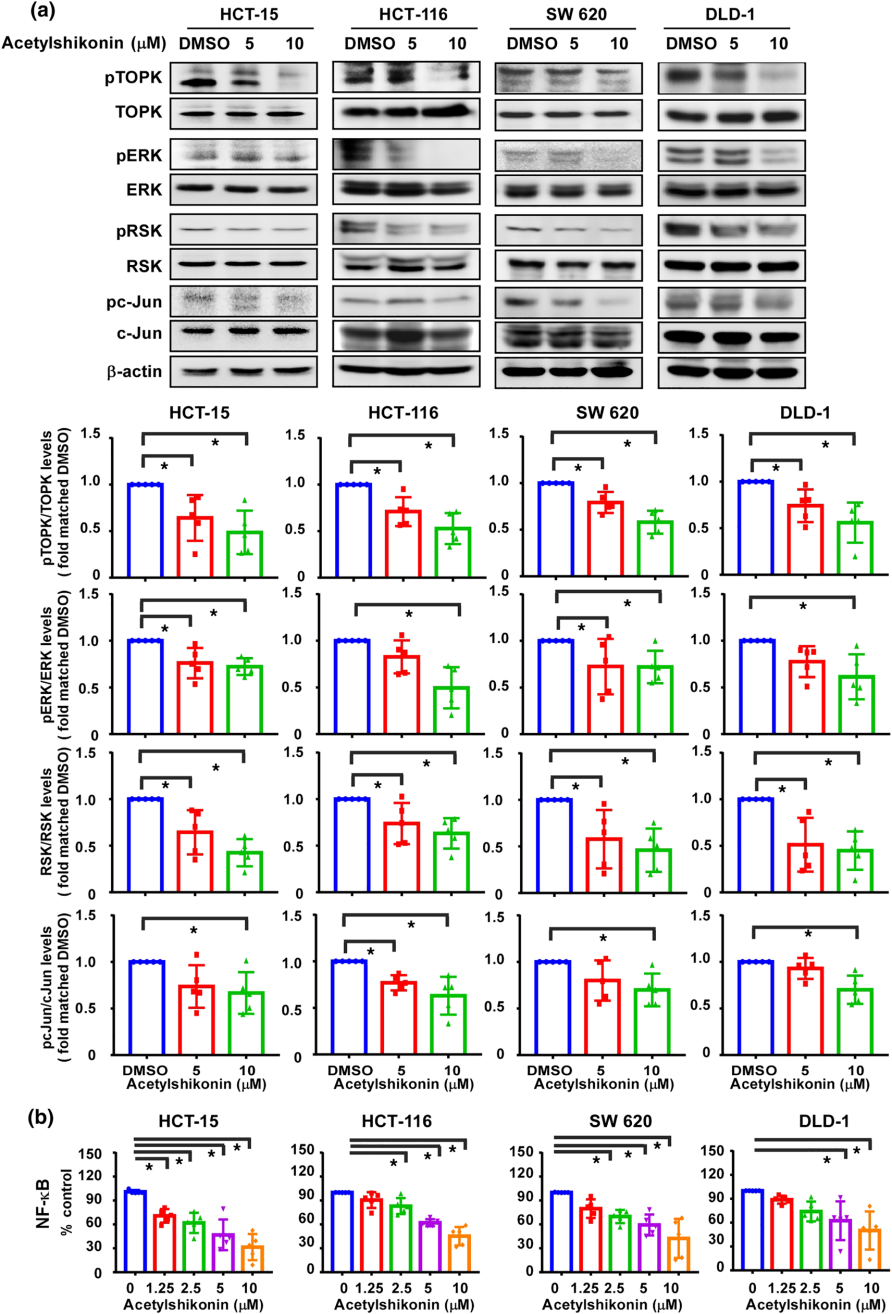
Acetylshikonin attenuates the expression of proteins involved in TOPK signalling. (a) The effect of acetylshikonin on TOPK signalling in colon cancer cells was assessed by western blot analysis followed by densitometric quantification. The cells were treated with 0‐, 5‐, or 10‐μM acetylshikonin and harvested at 24 hr, and then cell lysates were subjected to western blotting. Data shown are individual values with means± SD; n=5 independent experiments. *P < .05, significantly different as indicated. (b) The effect of acetylshikonin was evaluated in colon cancer cells transfected with an *NF‐κB* luciferase reporter plasmid. Data shown are individual values with means± SD; n=5 independent experiments. Data shown are individual values with means± SD; n=5 independent experiments

Because TOPK signalling pathways are involved in cell cycle, apoptosis, and cancer cell growth, we determined the effects of acetylshikonin on the expression of phosphorylated TOPK (Thr9), phosphorylated ERK (Thr202/Tyr204), phosphorylated RSK (Ser380), and phosphorylated c‐Jun (Ser73) as well as on the transcriptional activity of NF‐κB (Figure 4). Treatment of HCT‐15, HCT‐116, SW 620, or DLD‐1 colon cancer cells with acetylshikonin decreased the levels of phosphorylated TOPK (Thr9), phosphorylated ERK (Thr202/Tyr204), phosphorylated RSK (Ser380), and phosphorylated c‐Jun (Ser73; Figure 4a). The total levels of TOPK, ERK, RSK, and c‐Jun were unchanged as compared to DMSO controls (Figure 4a). Furthermore, transient transfection of HCT‐15, HCT‐116, SW 620, or DLD‐1 colon cancer cells with an *NF‐κB‐luciferase* plasmid followed by treatment with acetylshikonin showed significant inhibition of *NF‐κB* transcriptional activity (Figure 4b).

**FIGURE 5:**
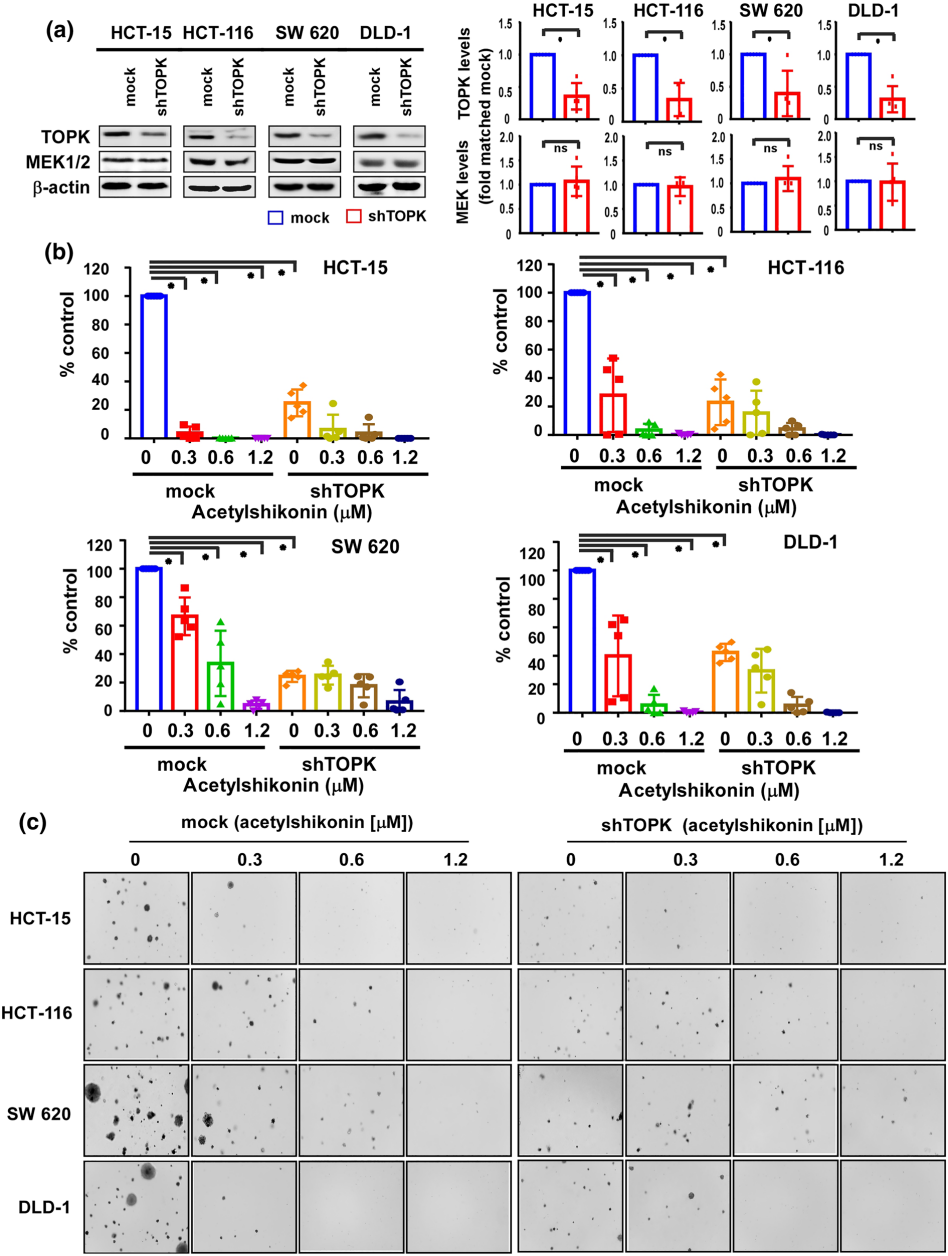
TOPK is a potential target in colon cancer cells. (a) The expression of TOPK in HCT‐15, HCT‐116, SW 620, and DLD‐1 cells expressing shRNA‐mock or shRNA‐TOPK was evaluated by western blotting. Data shown are individual values with means± SD; n=5 independent experiments. *P < .05, significantly different as indicated; ns, not significant. (b) Anchorage‐independent growth was assessed in HCT‐15, HCT‐116, SW 620, and DLD‐1 colon cancer cells expressing shRNA‐mock or shRNA‐TOPK. Data shown are individual values with means± SD; n=5 independent experiments. *P < .05, significantly different as indicated. (c) Representative photographs of the effects of TOPK knock‐down on anchorage‐independent growth are shown for HCT‐15, HCT‐116, SW 620, and DLD‐1 colon cancer cells

To confirm that the inhibition of TOPK expression impairs growth of colon cancer cells, we assessed cell proliferation after knock‐down of TOPK by transferring *shRNA‐TOPK* #1–4 into colon cancer cell lines. The anchorage‐independent cell growth was attenuated by *shRNA‐TOPK* #4 (Figure S3A–D). We confirmed that silencing of TOPK by shRNA abolished the expression of TOPK in HCT‐15, HCT‐116, SW 620, or DLD‐1 cells (Figure 5a) and treatment of these TOPK knock‐down cells with acetylshikonin failed to inhibit anchorage‐independent growth (Figure 5b,c).

### 3.5 Anti‐proliferative and apoptotic effects of acetylshikonin in HCT‐116 cells are independent of p53 status

**FIGURE 6:**
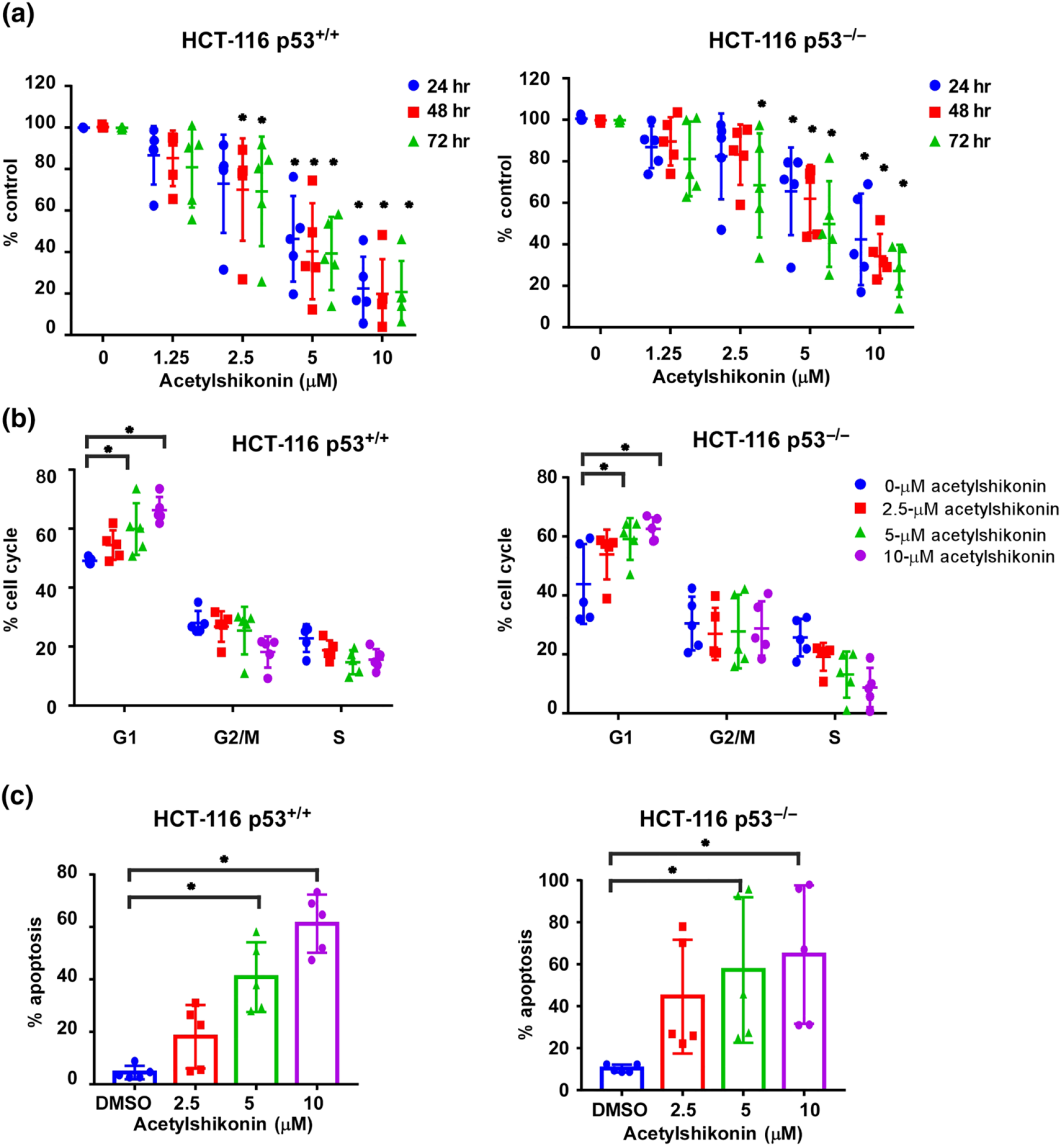
Acetylshikonin inhibits colon cancer cell growth irrespective of cellular p53 status. (a) The growth of HCT‐116 p53^+/+^ and p53^−/−^ cells was determined by MTT assay, after treatment with various concentrations of acetylshikonin (0, 1.25, 2.5, 5, or 10 μM) for 24, 48, or 72 hr, and similar results were obtained. Data shown are individual values with means± SD; n=5 independent experiments. *P < .05, significantly different as indicated. (b) The effect of treatment with acetylshikonin on cell cycle of HCT‐116 p53^+/+^ and p53^−/−^ cells was evaluated. Data shown are individual values with means± SD; n=5 independent experiments. *P < .05, significantly different as indicated. (c) The effect of acetylshikonin on apoptosis was assessed in HCT‐116 p53^+/+^ and p53^−/−^ cells. Data shown are individual values with means± SD; n=5 independent experiments. *P < .05, significantly different as indicated

To address whether the anti‐proliferative and apoptotic effects of acetylshikonin on colon cancer cells are dependent on intracellular p53, we treated HCT‐116 p53^+/+^ (p53 wild type) and HCT‐116 p53^−/−^ (p53 deficient) colon cancer cells with various concentrations (0, 1.25, 2.5, 5, or 10 μM) of acetylshikonin for 24, 48, or 72 hr (Figure 6). The results revealed that proliferation of HCT‐116 p53^+/+^ cells was significantly inhibited by treatment with acetylshikonin at 5 μM (Figure 6a). Cell cycle was arrested at G1 phase in both cell types irrespective of p53 status (Figure 6b). Treatment with 5‐μM acetylshikonin induced apoptosis in HCT‐116 p53^+/+^ and HCT‐116 p53^−/−^ cells by 40.87% and 57.26%, respectively (Figure 6c). We confirmed the expression of p53 in HCT‐116 53^+/+^ and HCT‐116 p53^−/−^ cells (Figure S4).

### 3.6 Acetylshikonin inhibits the growth of TOPK‐positive PDX tumours in SCID mice

**FIGURE 7:**
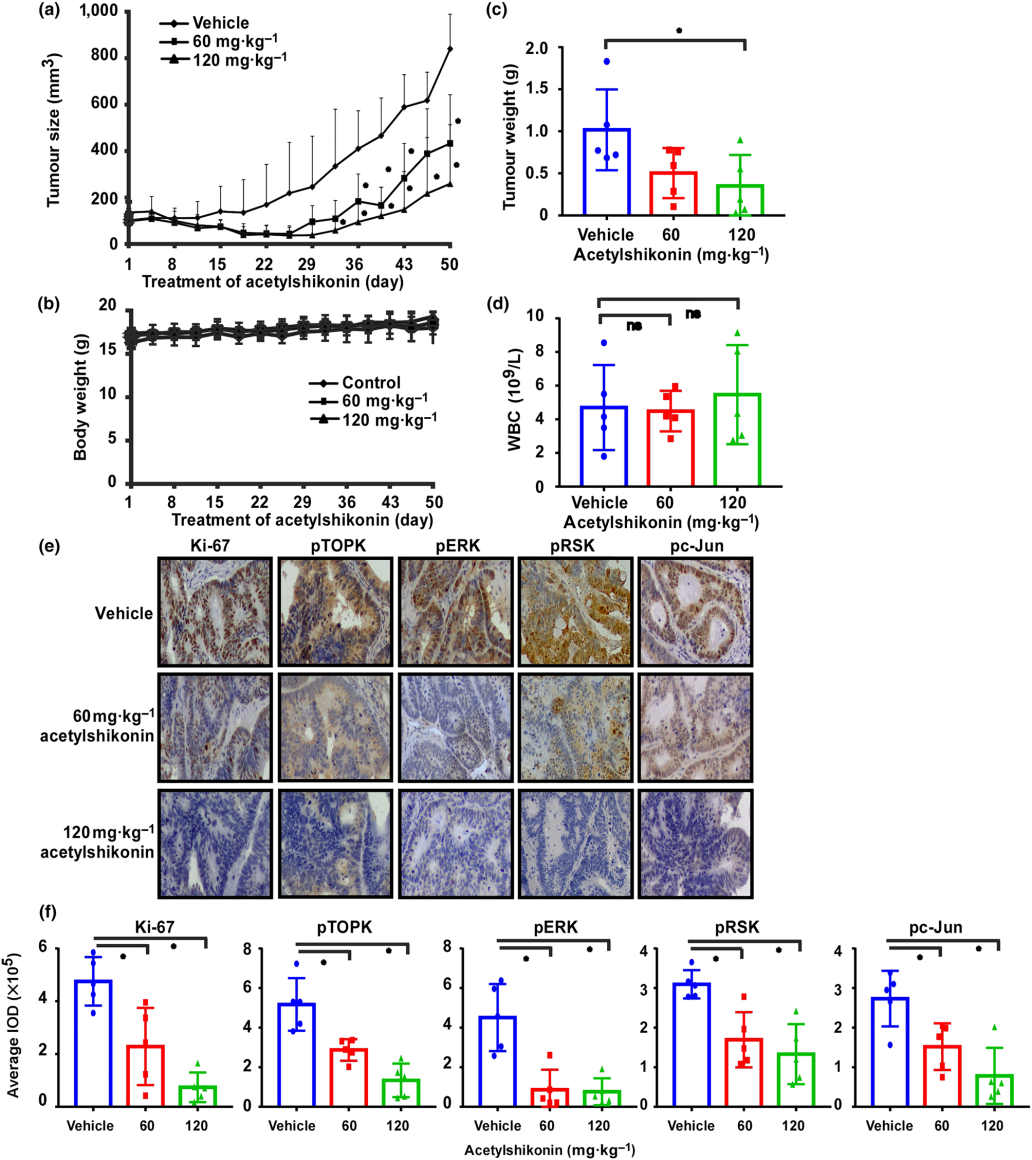
Acetylshikonin attenuates the growth of PDX tumours (HJG41) in mice. (a) The effect of acetylshikonin on the size of PDX tumours was assessed (as volume) over 50 days. Vehicle or acetylshikonin (60 or 120 mg·kg^−1^ body weight) was administered by gavage, and tumour volume was measured twice a week. Data are shown as mean values ± *SD,* from *n* = 5 in each group.. (b) No changes in the body weight were observed in mice treated with vehicle, 60 or 120 mg·kg^−1^ acetylshikonin. (c) Tumour weight was measured after treatment on the last day of the study. (d) White blood cell (WBC) count from PDX tumour‐bearing mice treated with vehicle or acetylshikonin (60 or 120 mg·kg^−1^). In (c ) and (d), Data shown are individual values with means± SD; n=5 mice per group. *P < .05, significantly different as indicated; ns, not significant. (e) The expression of Ki‐67, pTOPK, pERK, pRSK, or pc‐Jun was examined by immunohistochemical analysis (100× magnification). (f) The expression of Ki‐67, pTOPK, pERK, pRSK, or pc‐Jun was quantified from four separate areas on each slide and an average of five samples in vehicle‐ and acetylshikonin‐treated groups. Data shown are individual IOD values with means± SD; n=5. *P < .05, significantly different as indicated

To examine the anti‐tumour activity of acetylshikonin in vivo, we used three PDX models, HJG41, HJG175, and HJG152, which exhibited a range of levels of TOPK (Figures 7, S5, and S6). The PDX tumour mass was implanted into SCID mice, and then vehicle or acetylshikonin (60 or 120 mg·kg^−1^ body weight for HJG41 and HJG152, and 80 or 160 mg·kg^−1^ body weight for HJG175) was administered by oral gavage once a day for 50, 88, or 46 days for HJG41, HJG152, and HJG175, respectively. The results showed that treatment of HJG41 or HJG175 PDX tumour‐bearing mice with acetylshikonin at a dose of 120 or 160 mg·kg^−1^, respectively, significantly reduced tumour volume and weight compared to the vehicle‐treated group (Figures 7a,b and S6A, B), without changing body weight (Figures 7c and S6C). However, tumour growth of HJG152, which had low expression of TOPK, was not affected by treatment with acetylshikonin (Figure S6E–H). To further analyse haematopoietic toxicity, we counted the white blood cell (WBC) number after treatment with acetylshikonin. The results revealed that the WBC count was not affected by treatment with acetylshikonin (Figures 7d and S6D). Tumour tissues were prepared for IHC analysis, and the expression of Ki‐67, phosphorylated TOPK, ERK, RSK, or c‐Jun was examined (Figure 7e,f). The results indicated that expression of all these protein markers were significantly reduced after treatment with acetylshikonin in the HJG41 tumours compared with vehicle‐treated controls (Figure 7e,f).

## 4 DISCUSSION

Colorectal cancer is a major public health issue worldwide. Because of the involvement of diverse genetic and epigenetic alterations in colon cancer pathogenesis, a combination of therapies would improve therapeutic outcome. Despite substantial progress in developing anticancer therapies including the inhibitors of immune checkpoints and that of TK receptors for the treatment of colon cancer, novel therapeutic avenues are always being sought. Among the various oncogenic intracellular kinases, the TOPK family has been identified as a promising drug target (Hu et al., 2019; Kim et al., 2012; Sugimori et al., 2017). In this study, we have examined whether acetylshikonin, a natural diterpenoid, can inhibit colon carcinogenesis by acting as an inhibitor of TOPK. Acetylshikonin, a major bioactive component of leaf extracts of *L*. *erythrorhizon* root (Gwon et al., 2012; Pietrosiuk et al., 2006; Zhao, Wu, Wu, Zhao, & Chen, 2016), exhibits various pharmacological activities including induction of autophagy, anti‐oxidative, anti‐inflammatory, anti‐proliferative, anti‐fertility, and anticancer effects (He, Li, Su, Huang, & Zhu, 2016; Pietrosiuk et al., 2006; Skrzypczak et al., 2015; Wu et al., 2011; Zeng, Zhu, & Su, 2018). Several studies have demonstrated that the compound inhibits growth of a variety of cancers, including colorectal, breast, liver, medullary thyroid carcinoma, pancreas, melanoma, and gastric cancers (Cho & Choi, 2015; Hasenoehrl et al., 2017; Kim, Lee, Park, & Choi, 2016; Kretschmer et al., 2012; Park et al., 2017; Vukic et al., 2017; Zeng, Liu, & Zhou, 2009). Mechanistically, acetylshikonin has been reported to act by down‐regulating NF‐κB, https://www.guidetopharmacology.org/GRAC/ObjectDisplayForward?objectId=2844 expression, and CYP2J2 activity, and the https://www.guidetopharmacology.org/GRAC/LigandDisplayForward?ligandId=821/https://www.guidetopharmacology.org/GRAC/FamilyDisplayForward?familyId=738 axis, or by up‐regulating expression of https://www.guidetopharmacology.org/GRAC/ObjectDisplayForward?objectId=1441 (Cho et al., 2018; Cho & Choi, 2015). To identify a direct target for acetylshikonin, we examined the effect of acetylshikonin on the kinase activity of TOPK and MEK1 by an in vitro kinase assay (Figure 1a,b). Acetylshikonin clearly inhibited TOPK kinase activity (Figure 1a) but had no effect on MEK1 activity (Figure 1b). We used a kinase profiling assay to assess the effects of acetylshikonin on the kinase activity of several other kinases associated with TOPK signalling. The results indicated that acetylshikonin at 20 μM inhibited the kinase activity of Aurora A, Aurora B, and c‐Src but had no effect on the activity of JNK1, ERK1, PDK1, or PKB (Figure S1A). in vitro kinase assay further confirmed the inhibitory effects of acetylshikonin on Aurora A, Aurora B, and c‐Src kinases. However, the compound had no effect on the catalytic activity of these kinases at or below 2.5‐μM concentration (Figure S1B–D). We have made prediction of binding scores between acetylshikonin and TOPK and compared that with the binding score of a reported TOPK inhibitor, 3‐deoxysappanchalcone, by using computational modelling. The score between acetylshikonin to TOPK and that of 3‐deoxysappanchalcone to TOPK is −6.94 and −5.02, respectively (Zhao et al., 2018). Moreover, the ATP competitive pull‐down assay indicated that acetylshikonin might interact with the ATP‐binding pocket of TOPK. We also confirmed the ATP competitive binding of acetylshikonin with TOPK by kinetic analysis with an increasing *K*_M_ value 15 to 20 (Figure 1e,f). Kim et al. (2012) reported the synthesis of a TOPK inhibitor, HI‐TOPK‐032, which also inhibited MEK1 activity by 40%, whereas acetylshikonin interacted with TOPK directly and inhibited TOPK kinase activity without affecting the activity of MEK1 (Figure 1). The results of computer modelling and in vitro binding assays were further supported by significant inhibition of the growth of HCT‐15, HCT‐116, SW 620, and DLD‐1 colon cancer cell lines (Figure 2) as assessed by MTT and soft agar colony formation assays. The observed anti‐proliferative effects of acetylshikonin was in accordance with the report of Zeng et al. (2009), who reported that acetylshikonin inhibited proliferation of PANC1 cells (Cho & Choi, 2015). Whereas acetylshikonin inhibited proliferation of PANC1 cells at 12.5 μM, it induced apoptosis and G1 phase arrest in HCT‐15, HCT‐116, SW 620, and DLD‐1 colon cancer cell lines at 2.5 μM (Figure 3). This variation in effective concentration may be a cell type‐specific phenomenon. However, it is notable that these colon cancer cell lines (HCT‐15, HCT‐116, SW 620, and DLD‐1) express very high levels of TOPK (Figure S2B). It is interesting to note that acetylshikonin had no effect on proliferation of HT‐29 or SW 480 cells that express low levels of TOPK (Figure S2C). Our results suggest that TOPK is a major target of acetylshikonin to inhibit proliferation and induce apoptosis in colon cancer cells. Several signalling molecules, including ERK, RSK, and c‐Jun (Li et al., 2016; Zhu et al., 2007) are involved in TOPK signalling and acetylshikonin decreased the phosphorylation of TOPK, ERK, RSK, and c‐Jun and the transcriptional activity of NF‐κB (Figure 4). These results are in good agreement with other studies reporting that TOPK inhibition decreased the phosphorylation levels of MAPK signalling and NF‐κB transcriptional activity (Gao et al., 2017; Yang et al., 2016). Cheng et al. (2008) found that shikonin derivatives including acetylshikonin inhibited LPS‐induced NOS through the down‐regulation of NF‐κB transcriptional activation by suppression of ERK phosphorylation. Cho and Choi (2015) observed that acetylshikonin could inhibit cancer cell proliferation by regulating the NF‐κB transcriptional activity. Furthermore, acetylshikonin did not affect the proliferation of TOPK‐deficient cells (Figures 5b,c and S3B–D), suggesting the requirement of TOPK in acetylshikonin‐induced apoptosis in colon cancer cells. Although Lei and Fu reported the interaction of TOPK with mutant p53 or with the DNA binding domain of p53 (Hu et al., 2010; Lei et al., 2015), our results showed that acetylshikonin inhibited proliferation, arrested cell cycle, and induced apoptosis in both p53‐positive and p53‐negative cells. Thus, acetylshikonin can exert its anticancer effects independently of cellular p53 status (Figure 6).

The PDX mouse tumour model is a preclinical in vivo model that resembles human tumour growth (Nunes et al., 2015; Tentler et al., 2012). The inhibition of the growth of PDX tumours expressing high levels of TOPK in mice after treatment with acetylshikonin (Figure 7) further demonstrated the anti‐tumour effects of the compound. Compared with vehicle‐treated mice, mice given acetylshikonin p.o did not show changes in body weight or any marked signs of toxicity, thus indicating the potential of developing acetylshikonin as a novel therapy for colorectal cancer. It is interesting to note that the growth of the PDX tumours derived from patient HJG152 which expressed low levels of TOPK was not markedly affected by acetylshikonin (Figures S5A and 6e,f), whereas statistically significant inhibition of tumour volume and weight in the HJG175 PDX (medium TOPK expression) and in HJG41 PDX (high levels of TOPK expression) by acetylshikonin was observed. IHC analysis of xenograft tumours showed that acetylshikonin decreased the expression of Ki‐67, pTOPK, pERK, pRSK, or pc‐Jun (Figure 7e,f), further suggesting that the compound interrupted major components of TOPK signalling. Other inhibitors of TOPK, such as OTS514 and OTS964, induced haematopoietic toxicity as reported by decreased WBC count in an in vivo study (Matsuo et al., 2014; Nakamura et al., 2015). The present study revealed that acetylshikonin did not affect the WBC count in our mouse model (Figure 7d). It has been reported that some glioma stem cells are resistant to the TOPK inhibitor OTS964 (Sugimori et al., 2017). Whether acetylshikonin, as a TOPK inhibitor, can cause other serious side effects or can induce resistance in colon cancer cells needs to be examined in further investigations.

In summary, our study suggests that acetylshikonin can inhibit the growth of colorectal cancer tumours, in vitro and in vivo, by suppressing TOPK signalling through its direct targeting of TOPK, without modulation of MEK1 activity. As acetylshikonin inhibited tumour growth in the mouse model only when the PDX tumour tissue expressed high levels of TOPK, and showed no marked side effects, the compound or its derivatives can be considered for further clinical development of molecular target‐based therapy for colorectal cancer.

## CONFLICT OF INTEREST

The authors declare no conflicts of interest.

## AUTHOR CONTRIBUTIONS

R.Z., M.L., and Z.D. were involved in the study concept and design, acquisition of data, analysis and interpretation of data, and drafting of the manuscript. R.Z., L.W., H.C., F.Y., X.F., and K.L. performed the experiments. B.C. and M.F. provided material support. R.Z., M.L., A.B., and Z.D. wrote the manuscript. Z.D. and M.L. supervised the study. All authors read and approved the final manuscript.

## DECLARATION OF TRANSPARENCY AND SCIENTIFIC RIGOUR

This Declaration acknowledges that this paper adheres to the principles for transparent reporting and scientific rigour of preclinical research as stated in the *BJP* guidelines for https://bpspubs.onlinelibrary.wiley.com/doi/epdf/10.1111/bph.14207, https://bpspubs.onlinelibrary.wiley.com/doi/epdf/10.1111/bph.14208, and https://bpspubs.onlinelibrary.wiley.com/doi/epdf/10.1111/bph.14206, and as recommended by funding agencies, publishers and other organisations engaged with supporting research.

## Supporting information

Supporting info item

Supporting info item

**Figure S1.** The effect of acetylshikonin is assessed against several different kinases. (A) Kinase activity assay results of 7 different kinases indicate the effect of acetylshikonin at 20 μM. (B) *in vitro* kinase assay analysis the effect of acetylshikonin on Aurora A activity and densitometric quantification was evaluated by five independent experiments. Densitometric quantification data are shown as mean values ± S.D. The asterisks (* *p* < 0.05) indicate a significant inhibition of Aurora A activity treated acetylshikonin. (C) *in vitro* kinase assay analysis the effect of acetylshikonin on Aurora B activity and densitometric quantification was evaluated by five independent experiments. Densitometric quantification data are shown as mean values ± S.D. The asterisks (* *p* < 0.05) indicate a significant inhibition of Aurora B activity treated acetylshikonin and (D) *in vitro* kinase assay analysis the effect of acetylshikonin on c‐Src activity was evaluated by five independent experiments. Data are shown as mean values ± S.D. The asterisks (*p < 0.05) indicate a significant inhibition of c‐Src activity treated acetylshikonin.

**Figure S2.** Acetylshikonin suppresses growth of colon cancer cells by targeting TOPK. (A) Effects of acetylshikonin on normal CCD‐18Co colon cells. Data are shown as means ±S.D. of five independent experiments. The asterisks (*p < 0.05) indicate a significant difference between untreated control and acetylshikonin‐treated cells. (B) The expression of TOPK signaling pathway in colon cancer cells was assessed by Western blot analysis and densitometric quantification was evaluated (number of independent experiment *n*=5). Densitometric quantification data are shown as mean values ± S.D. The asterisks (* *p* < 0.05) indicate a significant different expression of TOPK signalling pathway in colon cancer cell lines. (C) Treatment of SW 480 and HT‐29 cells with acetylshikonin. Cells were treated with 0, 2.5, 5, or 10 μM acetylshikonin and proliferation was estimated by MTT assay at 24, 48, or 72 h (number of independent experiment *n*=5). Data are shown as mean values ±S.D. The asterisks (*p < 0.05) indicate a significant difference between untreated control and acetylshikonin‐treated cells.

**Figure S3.** TOPK enhances proliferation of DLD‐1 colon cancer cells. (A) The expression of TOPK in DLD‐1 cells which was infected shRNA‐mock or shRNA‐TOPK #1‐4 virus was evaluated by Western blotting and densitometric quantification was evaluated (number of independent experiment *n*=5). Densitometric quantification data are shown as mean values ± S.D. The asterisks (* *p* < 0.05) indicate a significant difference expression level of TOPK shRNA‐mock and shRNA‐TOPK‐expressing cells. (B) The effect of acetylshikonin on growth of DLD‐1 cells was estimated by MTS assay at 0, 24, 48, and 72 h (number of independent experiment *n*=5) Data are shown as means values ±S.D. (C) Anchorage‐independent growth was assessed in DLD‐1 cells expressing shRNA‐mock or shRNA‐TOPK (number of independent experiment n=5). Data are shown as means ±S.D. (D) Representative photos of anchorage‐independent colonies. Data are shown as mean value ±S.D. The asterisks (* *p* < 0.05) indicate a significant difference between shRNA‐mock and shRNA‐TOPK‐expressing cells, respectively.

**Figure S4.** The expression of p53 in HCT 116 p53^+/+^ and HCT 116 p53^‐/‐^ cells. Cells were evaluated by Western blotting with a p53 antibody and densitometric quantification was evaluated (number of independent experiment *n*=5). Densitometric quantification data are shown as mean values ± S.D. The asterisks (* *p* < 0.05) indicate a significant difference expression level of p53 between HCT 116 p53^+/+^ and HCT 116 p53^‐/‐^ cells.

**Figure S5.** The characteristics of patient tumor samples in the PDX mouse model. (A) Expression of TOPK in tumor samples used for the PDX mouse model and densitometric quantification was evaluated (number of independent experiment *n*=5). Densitometric quantification data are shown as mean values ± S.D. The asterisks (* *p* < 0.05) indicate a significant difference expression level of TOPK in the PDX mouse model. (B) Characteristics of patients (HJG41, HJG175, and HJG152) tumors were used in the PDX mouse model.

**Figure S6.** Acetylshikonin attenuates the growth of PDX tumors (HJG175 and HJG152) in mice. (A, E) The effect of acetylshikonin on the volume of PDX tumors (HJG175 and HJG152) was plotted over 46 and 88 days, respectively. Vehicle or acetylshikonin (80 or 160 mg/kg for HJG175 and 60 or 120 mg/kg for HJG 152) were administered by oral gavage. Tumor volume was measured twice a week, *n*=10 in each group for the case of HJG175 and *n*=8 in each group for the case of HJG152. The asterisk (* p < 0.05) indicates a significant decrease in volume of tumors from vehicle or acetylshikonin‐treated mice. Data are shown as mean values ± S.D. (B, F) PDX tumor weight from mice treated with vehicle or acetylshikonin. (C, G) No changes in body weight were observed in mice treated with vehicle, or acetylshikonin. (D, H) White blood cell (WBC) count from mice treated with vehicle or acetylshikonin (80 or 160 mg/kg for HJG175 and 60 or 120 mg/kg for HJG 152).

**Figure S7.** The expression of Ki‐67, pTOPK, pERK, pRSK, or pc‐Jun in HJG175 and HJG152 PDX tumors. (A) Representative photos of Ki‐67, pTOPK, pERK, pRSK, or pcJun expression (B) Quantified graphs of the expression of Ki‐67, pTOPK, pERK, pRSK, or pc‐Jun. Each sample was quantified from 4 separate areas on each slide and an average of *n*=5 (vehicle and treatment) samples per group. Data are expressed as IOD values ±S.D. The asterisks (* p < 0.05) indicate a significance difference between treated tissues compared to untreated controls.

## References

[bph14981-bib-0001] Abe, Y. , Matsumoto, S. , Kito, K. , & Ueda, N. (2000). Cloning and expression of a novel MAPKK‐like protein kinase, lymphokine‐activated killer T‐cell‐originated protein kinase, specifically expressed in the testis and activated lymphoid cells. The Journal of Biological Chemistry, 275(28), 21525–21531. 10.1074/jbc.M909629199 10781613

[bph14981-bib-0002] Aksamitiene, E. , Kholodenko, B. N. , Kolch, W. , Hoek, J. B. , & Kiyatkin, A. (2010). PI3K/Akt‐sensitive MEK‐independent compensatory circuit of ERK activation in ER‐positive PI3K‐mutant T47D breast cancer cells. Cellular Signalling, 22(9), 1369–1378. 10.1016/j.cellsig.2010.05.006 20471474 PMC2893265

[bph14981-bib-0003] Alexander, S. P. H. , Fabbro, D. , Kelly, E. , Mathie, A. , Peters, J. A. , Veale, E. L. , … Collaborators, C. G. T. P. (2019). The Concise Guide to PHARMACOLOGY 2019/20: Enzymes. British Journal of Pharmacology, 176, S297–S396. 10.1111/bph.14752 31710714 PMC6844577

[bph14981-bib-0004] Alexander, S. P. H. , Kelly, E. , Mathie, A. , Peters, J. A. , Veale, E. L. , Faccenda, E. , … Collaborators, C. G. T. P. (2019). The Concise Guide to PHARMACOLOGY 2019/20: Introduction and Other Protein Targets. British Journal of Pharmacology, 176, S1–S20. 10.1111/bph.14747 31710719 PMC6844537

[bph14981-bib-0005] Cheng, Y. W. , Chang, C. Y. , Lin, K. L. , Hu, C. M. , Lin, C. H. , & Kang, J. J. (2008). Shikonin derivatives inhibited LPS‐induced NOS in RAW 264.7 cells via downregulation of MAPK/NF‐κB signaling. Journal of Ethnopharmacology, 120(2), 264–271. 10.1016/j.jep.2008.09.002 18835347

[bph14981-bib-0006] Cho, B. H. , Jung, Y. H. , Kim, D. J. , Woo, B. H. , Jung, J. E. , Lee, J. H. , … Park, H. R. (2018). Acetylshikonin suppresses invasion of Porphyromonas gingivalis infected YD10B oral cancer cells by modulating the interleukin‐8/matrix metalloproteinase axis. Molecular Medicine Reports, 17(2), 2327–2334. 10.3892/mmr.2017.8151 29207110 PMC5783479

[bph14981-bib-0007] Cho, M. , Paik, Y. , & Hahn, T. (1999). Physical stability of shikonin derivatives from the roots of *Lithospermum erythrorhizon* cultivated in Korea. Journal of Agricultural and Food Chemistry, 47(10), 4117–4120. 10.1021/jf9902853 10552776

[bph14981-bib-0008] Cho, S. C. , & Choi, B. Y. (2015). Acetylshikonin inhibits human pancreatic PANC‐1 cancer cell proliferation by suppressing the NF‐κB activity. Biomolecules & Therapeutics (Seoul), 23(5), 428–433. 10.4062/biomolther.2015.102 PMC4556202 26336582

[bph14981-bib-0009] Curtis, M. J. , Alexander, S. , Cirino, G. , Docherty, J. R. , George, C. H. , Giembycz, M. A. , … Ahluwalia, A. (2018). Experimental design and analysis and their reporting II updated and simplified guidance for authors and peer reviewer. British Journal of Pharmacology, 175, 987–993. 10.1111/bph.14153 29520785 PMC5843711

[bph14981-bib-0010] Curtis, M. J. , Bond, R. A. , Spina, D. , Ahluwalia, A. , Alexander, S. P. A. , Giembycz Mark, A. , … McGrath, J. C. (2015). Experimental design and analysis and their reporting: New guidance for publication in BJP. British Journal of Pharmacology, 172, 3461–3471. 10.1111/bph.12856 26114403 PMC4507152

[bph14981-bib-0011] Davide, C. , Vitiello, P. P. , Cardone, C. , Martini, G. , Troiani, T. , Martinelli, E. , & Ciardiello, F. (2019). Immunotherapy of colorectal cancer: Challenges for therapeutic efficacy. Cancer Treatment Reviews, 76(2019), 22–32. 31079031 10.1016/j.ctrv.2019.04.003

[bph14981-bib-0012] Gao, G. , Zhang, T. , Wang, Q. , Reddy, K. , Chen, H. , Yao, K. , … Dong, Z. (2017). ADA‐07 suppresses solar ultraviolet‐induced skin carcinogenesis by directly inhibiting TOPK. Molecular Cancer Therapeutics, 16(9), 1843–1854. 10.1158/1535-7163.MCT-17-0212 28655782 PMC5587387

[bph14981-bib-0013] Gwon, S. Y. , Ahn, J. Y. , Chung, C. H. , Moon, B. , & Ha, T. Y. (2012). *Lithospermum erythrorhizon* suppresses high‐fat diet‐induced obesity, and acetylshikonin, a main compound of *Lithospermum erythrorhizon*, inhibits adipocyte differentiation. Journal of Agricultural and Food Chemistry, 60(36), 9089–9096. 10.1021/jf3017404 22900585

[bph14981-bib-0014] Harding, S. D. , Sharman, J. L. , Faccenda, E. , Southan, C. , Pawson, A. J. , Ireland, S. , … NC‐IUPHAR . (2018). The IUPHAR/BPS Guide to pharmacology in 2018: Updates and expansion to encompass the new guide to immunopharmacology. Nucleic Acids Research, 46(D1), D1091–D1106. 10.1093/nar/gkx1121 29149325 PMC5753190

[bph14981-bib-0015] Hasenoehrl, C. , Schwach, G. , Ghaffari‐Tabrizi‐Wizsy, N. , Fuchs, R. , Kretschmer, N. , Bauer, R. , & Pfragner, R. (2017). Anti‐tumor effects of shikonin derivatives on human medullary thyroid carcinoma cells. Endocrine Connections, 6(2), 53–62. 10.1530/EC-16-0105 28069896 PMC5424774

[bph14981-bib-0016] He, Y. , Li, Q. , Su, M. , Huang, W. , & Zhu, B. (2016). Acetylshikonin from Zicao exerts antifertility effects at high dose in rats by suppressing the secretion of GTH. Biochemical and Biophysical Research Communications, 476(4), 560–565. 10.1016/j.bbrc.2016.05.162 27264949

[bph14981-bib-0017] Hu, F. , Gartenhaus, R. B. , Eichberg, D. , Liu, Z. , Fang, H. B. , & Rapoport, A. P. (2010). PBK/TOPK interacts with the DBD domain of tumor suppressor p53 and modulates expression of transcriptional targets including p21. Oncogene, 29(40), 5464–5474. 20622899 10.1038/onc.2010.275

[bph14981-bib-0018] Hu, Q. , Gao, T. , Shi, Y. , Lei, Q. , Liu, Z. , Feng, Q. , … Yu, L. T. (2019). Design, synthesis and biological evaluation of novel 1‐phenyl phenanthridin‐6(5*H*)‐one derivatives as anti‐tumor agents targeting TOPK. European Journal of Medicinal Chemistry, 162, 407–422. 30453248 10.1016/j.ejmech.2018.11.007

[bph14981-bib-0019] Ikeda, Y. , Park, J. H. , Miyamoto, T. , Takamatsu, N. , Kato, T. , Iwasa, A. , … Hasegawa, K. (2016). T‐LAK cell‐originated protein kinase (TOPK) as a prognostic factor and a potential therapeutic target in ovarian cancer. Clinical Cancer Research, 22(24), 6110–6117. 10.1158/1078-0432.CCR-16-0207 27334838

[bph14981-bib-0020] Kilkenny, C. , Browne, W. , Cuthill, I. C. , Emerson, M. , Altman, D. G. , & NC3Rs Reporting Guidelines Working Group . (2010). Animal research: Reporting in vivo experiments: The ARRIVE guidelines. British Journal of Pharmacology, 160, 1577–1579. 20649561 10.1111/j.1476-5381.2010.00872.x PMC2936830

[bph14981-bib-0021] Kim, D. J. , Lee, J. H. , Park, H. R. , & Choi, Y. W. (2016). Acetylshikonin inhibits growth of oral squamous cell carcinoma by inducing apoptosis. Archives of Oral Biology, 70, 149–157. 10.1016/j.archoralbio.2016.06.020 27371806

[bph14981-bib-0022] Kim, D. J. , Li, Y. , Reddy, K. , Lee, M. H. , Kim, M. O. , Cho, Y. Y. , … Dong, Z. (2012). Novel TOPK inhibitor HI‐TOPK‐032 effectively suppresses colon cancer growth. Cancer Research, 72(12), 3060–3068. 10.1158/0008-5472.CAN-11-3851 22523035

[bph14981-bib-0023] Kolligs, F. T. (2016). Diagnostics and epidemiology of colorectal cancer. Visceral Medicine, 32(3), 158–164. 10.1159/000446488 27493942 PMC4945785

[bph14981-bib-0024] Kretschmer, N. , Rinner, B. , Deutsch, A. J. , Lohberger, B. , Knausz, H. , Kunert, O. , … Bauer, R. (2012). Naphthoquinones from *Onosma paniculata* induce cell‐cycle arrest and apoptosis in melanoma cells. Journal of Natural Products, 75(5), 865–869. 10.1021/np2006499 22530779 PMC3361261

[bph14981-bib-0025] Lai, Y. , Wei, X. , Lin, S. , Qin, L. , Cheng, L. , & Li, P. (2017). Current status and perspectives of patient‐derived xenograft models in cancer research. Journal of Hematology & Oncology, 10(1), 106 10.1186/s13045-017-0470-7 28499452 PMC5427553

[bph14981-bib-0026] Lei, B. , Liu, S. , Qi, W. , Zhao, Y. , Li, Y. , Lin, N. , … Shen, H. (2013). PBK/TOPK expression in non‐small‐cell lung cancer: Its correlation and prognostic significance with Ki67 and p53 expression. Histopathology, 63(5), 696–703. 24025073 10.1111/his.12215

[bph14981-bib-0027] Lei, B. , Qi, W. , Zhao, Y. , Li, Y. , Liu, S. , Xu, X. , … Shen, H. (2015). PBK/TOPK expression correlates with mutant p53 and affects patients' prognosis and cell proliferation and viability in lung adenocarcinoma. Human Pathology, 46(2), 217–224. 10.1016/j.humpath.2014.07.026 25466965

[bph14981-bib-0028] Li, S. , Zhu, F. , Zykova, T. , Kim, M. O. , Cho, Y. Y. , Bode, A. M. , … Dong, Z. (2011). T‐LAK cell‐originated protein kinase (TOPK) phosphorylation of MKP1 protein prevents solar ultraviolet light‐induced inflammation through inhibition of the p38 protein signaling pathway. The Journal of Biological Chemistry, 286(34), 29601–29609. 10.1074/jbc.M111.225813 21715333 PMC3191001

[bph14981-bib-0029] Li, Y. , Yang, Z. L. W. , Xu, S. , Wang, T. , Wang, T. , Niu, M. , … Li, S. (2016). TOPK promotes lung cancer resistance to EGFR tyrosine kinase inhibitors by phosphorylating and activating c‐Jun. Oncotarget, 7(6), 6748–6764. 10.18632/oncotarget.6826 26745678 PMC4872746

[bph14981-bib-0030] Matsuo, Y. , Park, J. H. M. T. , Yamamoto, S. , Hisada, S. , Alachkar, H. , & Nakamura, Y. (2014). TOPK inhibitor induces complete tumor regression in xenograft models of human cancer through inhibition of cytokinesis. Science Translational Medicine, 6(259), 259ra145. 10.1126/scitranslmed.3010277 25338756

[bph14981-bib-0031] Nakamura, Y. , Matsuo, Y. , Hisada, S. , Ahmed, F. , Huntley, R. , Sajjadi‐Hashemi, Z. , … Jenkins, D. M. (2015). Tricyclic compounds and PBK inhibitors containing the same. US patent: 9453025.

[bph14981-bib-0032] Nandi, A. (2004). Protein expression of PDZ‐binding kinase is up‐regulated in hematologic malignancies and strongly down‐regulated during terminal differentiation of HL‐60 leukemic cells. Blood Cells, Molecules, and Diseases, 32(1), 240–245. 10.1016/j.bcmd.2003.10.004 14757441

[bph14981-bib-0033] Nunes, M. , Vrignaud, P. , Vacher, S. , Richon, S. , Lievre, A. , Cacheux, W. , … Dangles‐Marie, V. (2015). Evaluating patient‐derived colorectal cancer xenografts as preclinical models by comparison with patient clinical data. Cancer Research, 75(8), 1560–1566. 10.1158/0008-5472.CAN-14-1590 25712343

[bph14981-bib-0034] Oh, S. M. , Zhu, F. , Cho, Y. Y. , Lee, K. W. , Kang, B. S. , Kim, H. G. , … Dong, Z. (2007). T‐lymphokine‐activated killer cell‐originated protein kinase functions as a positive regulator of c‐Jun‐NH2‐kinase 1 signaling and H‐Ras‐induced cell transformation. Cancer Research, 67(11), 5186–5194. 10.1158/0008-5472.CAN-06-4506 17545598

[bph14981-bib-0035] Ohashi, T. , Komatsu, S. , Ichikawa, D. , Miyamae, M. , Okajima, W. , Imamura, T. , … Otsuji, E. (2017). Overexpression of PBK/TOPK relates to tumour malignant potential and poor outcome of gastric carcinoma. British Journal of Cancer, 116(2), 218–226. 10.1038/bjc.2016.394 27898655 PMC5243986

[bph14981-bib-0036] Ohashi, T. , Komatsu, S. , Ichikawa, D. , Miyamae, M. , Okajima, W. , Imamura, T. , … Otsuji, E. (2016). Overexpression of PBK/TOPK contributes to tumor development and poor outcome of esophageal squamous cell carcinoma. Anticancer Research, 36(12), 6457–6466. 10.21873/anticanres.11244 27919968

[bph14981-bib-0037] Park, J. H. , Jeong, Y. J. , Won, H. K. , Choi, S. Y. , Park, J. H. , & Oh, S. M. (2014). Activation of TOPK by lipopolysaccharide promotes induction of inducible nitric oxide synthase through NF‐κB activity in leukemia cells. Cellular Signalling, 26(5), 849–856. 10.1016/j.cellsig.2014.01.004 24440499

[bph14981-bib-0038] Park, S. H. , Phuc, N. M. , Lee, J. , Wu, Z. , Kim, J. , Kim, H. , … Liu, K. H. (2017). Identification of acetylshikonin as the novel CYP2J2 inhibitor with anti‐cancer activity in HepG2 cells. Phytomedicine, 24, 134–140. 10.1016/j.phymed.2016.12.001 28160853

[bph14981-bib-0039] Pietrosiuk, A. , Syklowska‐Baranek, K. , Wiedenfeld, H. , Wolinowska, R. , Furmanowa, M. , & Jaroszyk, E. (2006). The shikonin derivatives and pyrrolizidine alkaloids in hairy root cultures of *Lithospermum canescens* (Michx.) Lehm. Plant Cell Reports, 25(10), 1052–1058. 10.1007/s00299-006-0161-2 16670900

[bph14981-bib-0040] Rajasekar, S. , Park, D. J. , Park, C. , Park, S. , Park, Y. H. , Kim, S. T. , … Choi, Y. W. (2012). In vitro and in vivo anticancer effects of *Lithospermum erythrorhizon* extract on B16F10 murine melanoma. Journal of Ethnopharmacology, 144(2), 335–345. 10.1016/j.jep.2012.09.017 22995444

[bph14981-bib-0041] Roh, E. , Lee, M. , Zykova, T. A. , Zhu, F. , Nadas, J. , Kim, H. G. , … Dong, Z. (2018). Targeting PRPK and TOPK for skin cancer prevention and therapy. Oncogene, 37(42), 5633–5647. 10.1038/s41388-018-0350-9 29904102 PMC6195829

[bph14981-bib-0042] Rossi, M. , Anwar, M. J. , Usman, A. , Keshavarzian, A. , & Bishehsari, F. (2018). Colorectal cancer and alcohol consumption‐populations to molecules. Cancers, 10(2), 1–17. 10.3390/cancers10020038 PMC5836070 29385712

[bph14981-bib-0043] Roswall, N. , & Weiderpass, E. (2015). Alcohol as a risk factor for cancer: Existing evidence in a global perspective. Journal of Preventive Medicine and Public Health, 48(1), 1–9. 25652705 10.3961/jpmph.14.052 PMC4322512

[bph14981-bib-0044] Schrödinger . (2017). Schrödinger (2017). New York, NY: LLC.

[bph14981-bib-0045] Siegel, R. L. , Miller, K. D. , Fedewa, S. A. , Ahnen, D. J. , Meester, R. G. S. , Barzi, A. , & Jemal, A. (2017). Colorectal cancer statistics, 2017. CA: A Cancer Journal for Clinicians, 67(3), 177–193. 10.3322/caac.21395 28248415

[bph14981-bib-0046] Simons‐Evelyn, M. , Bailey‐Dell, K. , Toretsky, J. A. , Ross, D. D. , Fenton, R. , Kalvakolanu, D. , & Rapoport, A. P. (2001). PBK/TOPK is a novel mitotic kinase which is upregulated in Burkitt's lymphoma and other highly proliferative malignant cells. Blood Cells, Molecules & Diseases, 27(5), 825–829. 10.1006/bcmd.2001.0452 11783945

[bph14981-bib-0047] Skrzypczak, A. , Przystupa, N. , Zgadzaj, A. , Parzonko, A. , Syklowska‐Baranek, K. , Paradowska, K. , & Nałęcz‐Jawecki, G. (2015). Antigenotoxic, anti‐photogenotoxic and antioxidant activities of natural naphthoquinone shikonin and acetylshikonin and Arnebia euchroma callus extracts evaluated by the umu‐test and EPR method. Toxicology in Vitro, 30(1 Pt B), 364–372. 10.1016/j.tiv.2015.09.029 26434532

[bph14981-bib-0048] Sugimori, M. , Hayakawa, Y. , Koh, M. , Hayashi, T. , Tamura, R. , & Kuroda, S. (2017). Targeting the T‐Lak cell originated protein kinase by OTS964 shrinks the size of power‐law coded heterogeneous glioma stem cell populations. Oncotarget, 9(3), 3043–3059. 10.18632/oncotarget.23077 29423027 PMC5790444

[bph14981-bib-0049] Tentler, J. J. , Tan, A. C. , Weekes, C. D. , Jimeno, A. , Leong, S. , Pitts, T. M. , … Eckhardt, S. G. (2012). Patient‐derived tumour xenografts as models for oncology drug development. Nature Reviews. Clinical Oncology, 9(6), 338–350. 10.1038/nrclinonc.2012.61 PMC3928688 22508028

[bph14981-bib-0050] Vukic, M. D. , Vukovic, N. L. , Obradovic, A. D. , Popovic, S. L. , Zaric, M. M. , Djurdjevic, P. M. , … Baskic, D. D. (2017). Naphthoquinone rich *Onosma visianii* Clem (*Boraginaceae*) root extracts induce apoptosis and cell cycle arrest in HCT‐116 and MDA‐MB‐231 cancer cell lines. Natural Product Research, 1–5. 10.1080/14786419.2017.1374271 28882053

[bph14981-bib-0051] Wu, Y. Y. , Zhu, L. , Ma, X. Y. , Shao, Z. J. , Chen, J. , Chen, X. J. , … Zhou, L. M. (2011). The anti‐proliferation effect of Aikete injection on hepatocellular carcinoma in vitro and in vivo. Pharmaceutical Biology, 49(5), 531–538. 10.3109/13880209.2010.524652 21385105

[bph14981-bib-0052] Xiao, J. , Duan, Q. , Wang, Z. , Yan, W. , Sun, H. , Xue, P. , … Zhu, F. (2016). Phosphorylation of TOPK at Y74, Y272 by Src increases the stability of TOPK and promotes tumorigenesis of colon. Oncotarget, 7(17), 24483–24494. 10.18632/oncotarget.8231 27016416 PMC5029716

[bph14981-bib-0053] Yang, J. , Yuan, D. , Xing, T. , Su, H. , Zhang, S. , Wen, J. , … Dang, D. (2016). Ginsenoside Rh2 inhibiting HCT116 colon cancer cell proliferation through blocking PDZ‐binding kinase/T‐LAK cell‐originated protein kinase. Journal of Ginseng Research, 40(4), 400–408. 10.1016/j.jgr.2016.03.007 27746693 PMC5052442

[bph14981-bib-0054] Zeng, J. , Zhu, B. , & Su, M. (2018). Autophagy is involved in acetylshikonin ameliorating non‐alcoholic steatohepatitis through AMPK/mTOR pathway. Biochemical and Biophysical Research Communications, 2018, 1–6. 10.1016/j.bbrc.2018.07.094 30055803

[bph14981-bib-0055] Zeng, Y. , Liu, G. , & Zhou, L.‐M. (2009). Inhibitory effect of acetylshikonin on human gastric carcinoma cell line SGC‐7901 in vitro and in vivo. World Journal of Gastroenterology, 15(15), 1816–1820. 19370777 10.3748/wjg.15.1816 PMC2670407

[bph14981-bib-0056] Zhao, R. , Huang, H. , Choi, B. Y. , Liu, X. , Zhang, M. , Zhou, S. , … Lee, M. H. (2018). Cell growth inhibition by 3‐deoxysappanchalcone is mediated by directly targeting the TOPK signaling pathway in colon cancer. Phytomedicine, 61(2019), 152813. 31035049 10.1016/j.phymed.2018.12.036

[bph14981-bib-0057] Zhao, W. , Wu, Z. , Wu, X. , Zhao, H. , & Chen, X. (2016). Determination of five naphthaquinones in Arnebia euchroma by quantitative analysis multi‐components with single‐marker. Zhongguo Zhong Yao Za Zhi, 41(20), 3792–3797. 28929657 10.4268/cjcmm20162014

[bph14981-bib-0058] Zhu, F. , Zykova, T. A. , Kang, B. S. , Wang, Z. , Ebeling, M. C. , Abe, Y. , … Dong, Z. (2007). Bidirectional signals transduced by TOPK‐ERK interaction increase tumorigenesis of HCT116 colorectal cancer cells. Gastroenterology, 133(1), 219–231. 10.1053/j.gastro.2007.04.048 17631144

[bph14981-bib-0059] Zlobec, I. , Molinari, F. , Kovac, M. , Bihl, M. P. , Altermatt, H. J. , Diebold, J. , … Lugli, A. (2010). Prognostic and predictive value of TOPK stratified by KRAS and BRAF gene alterations in sporadic, hereditary and metastatic colorectal cancer patients. British Journal of Cancer, 102(1), 151–161. 10.1038/sj.bjc.6605452 19935791 PMC2813744

[bph14981-bib-0060] Zykova, T. , Zhu, F. , Wang, L. , Li, H. , Lim, D. Y. , Yao, K. , … Dong, Z. (2018). Targeting PRPK function blocks colon cancer metastasis. Molecular Cancer Therapeutics, 17(5), 1101–1113. 10.1158/1535-7163.MCT-17-0628 29483219 PMC5940014

[bph14981-bib-0061] Zykova, T. A. , Zhu, F. , Vakorina, T. I. , Zhang, J. , Higgins, L. A. , Urusova, D. V. , … Dong, Z. (2010). T‐LAK cell‐originated protein kinase (TOPK) phosphorylation of Prx1 at Ser‐32 prevents UVB‐induced apoptosis in RPMI7951 melanoma cells through the regulation of Prx1 peroxidase activity. The Journal of Biological Chemistry, 285(38), 29138–29146. 10.1074/jbc.M110.135905 20647304 PMC2937944

[bph14981-bib-0062] Zykova, T. A. , Zhu, F. , Wang, L. , Li, H. , Bai, R. , Lim, D. Y. , … Dong, Z. (2017). The T‐LAK cell‐originated protein kinase signal pathway promotes colorectal cancer metastasis. eBioMedicine, 18, 73–82. 10.1016/j.ebiom.2017.04.003 28412249 PMC5405196

