# Supplementary material for: Acetylshikonin suppressed growth of colorectal tumour tissue and cells by inhibiting the intracellular kinase, T‐lymphokine‐activated killer cell‐originated protein kinase
Source: Br J Pharmacol. 2020 Apr 10;177(10):2303–19. doi: 10.1111/bph.14981 (PMC7174886; doi:10.1111/bph.14981)

Figure 2

A

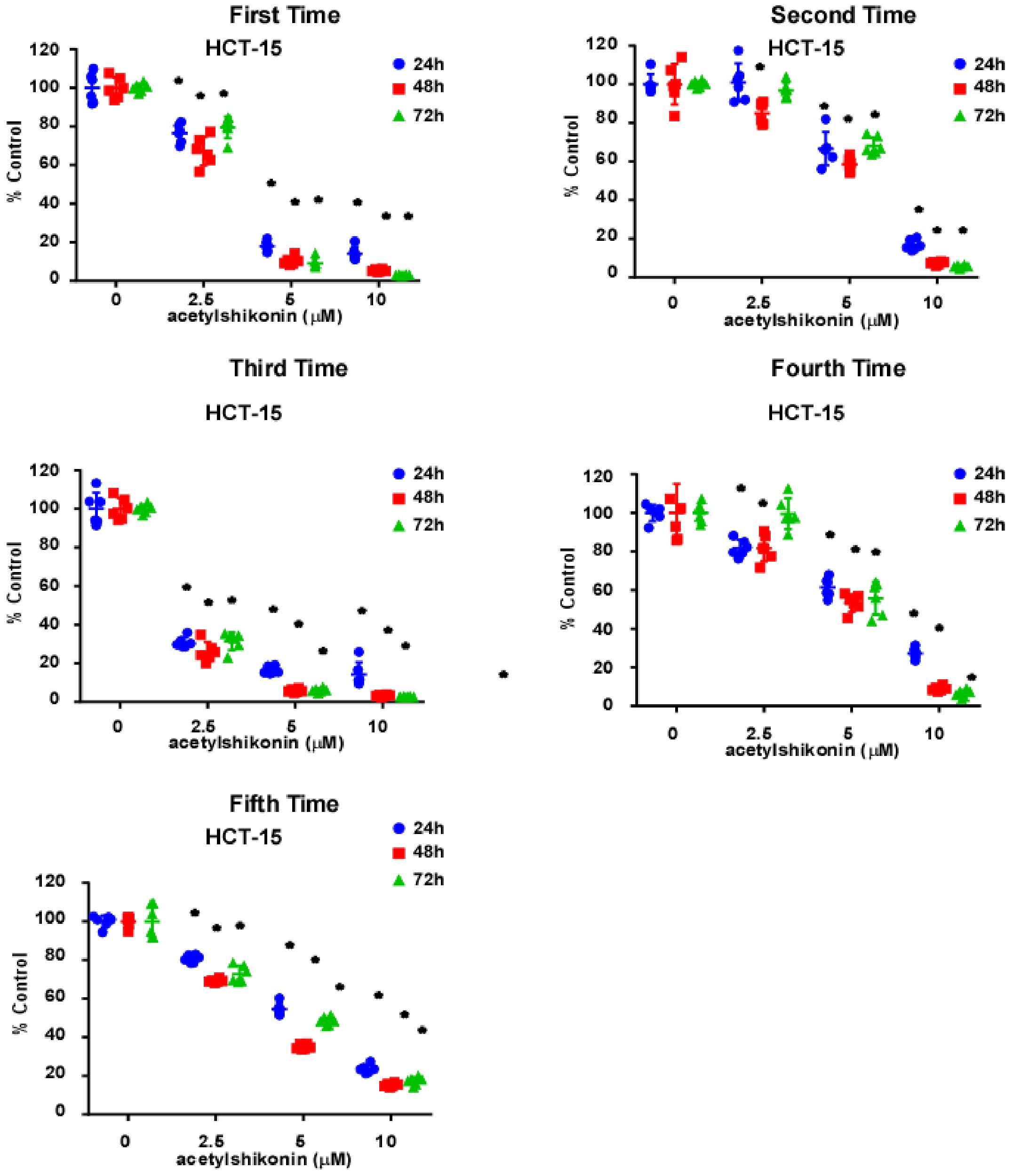
First Time
HCT-15
Second Time
HCT-15
Third Time
HCT-15
Fourth Time
HCT-15
Fifth Time
HCT-15
24h
48h
72h
% Control
120
100
80
60
40
20
0
0
2.5
5
10
acetylshikonin (μM)

Figure 2

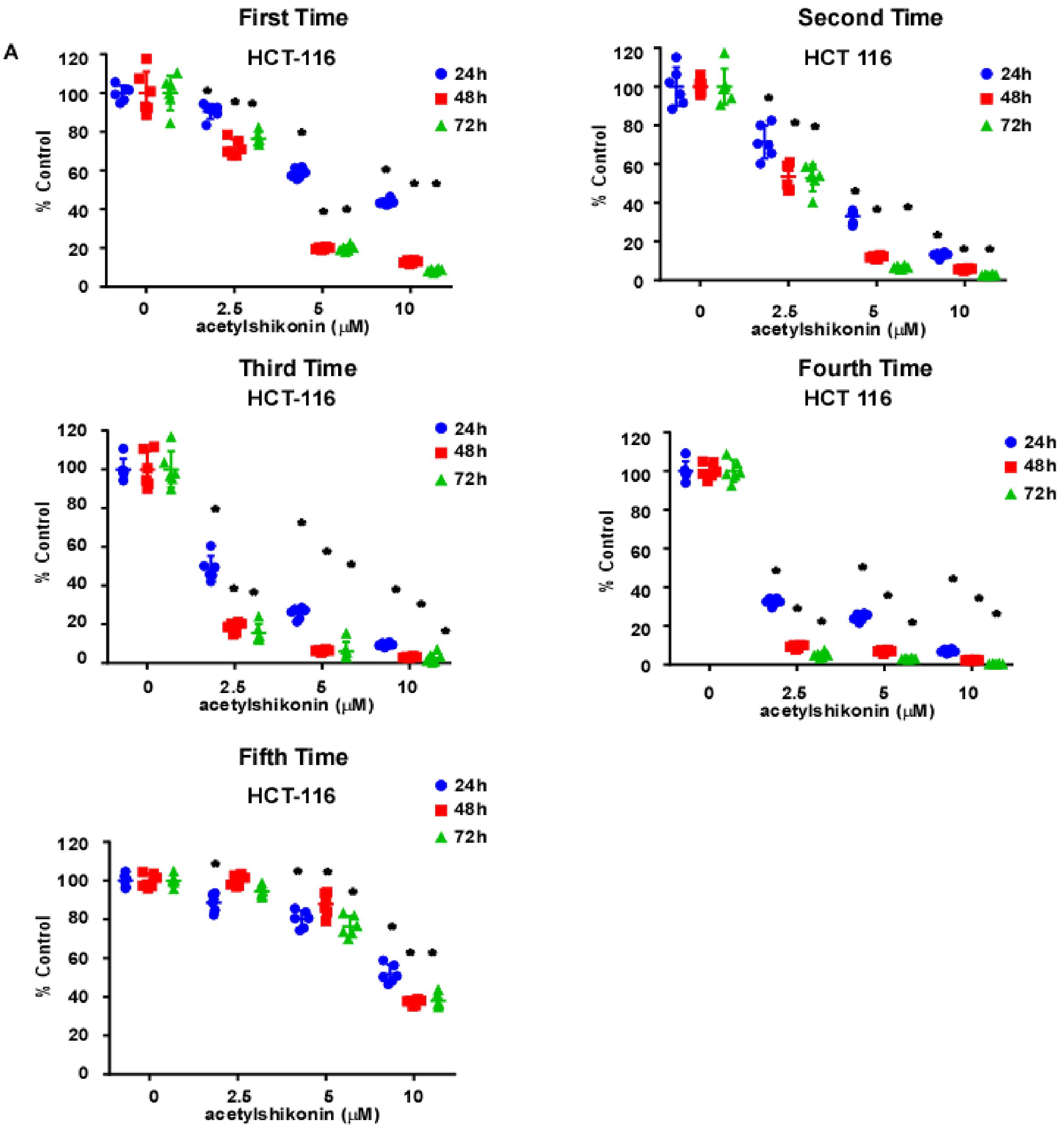
A
First Time
HCT-116
24h
48h
72h
% Control
acetylshikonin (μM)
0
2.5
5
10
Second Time
HCT 116
Third Time
HCT-116
Fourth Time
HCT 116
Fifth Time
HCT-116

Figure 2

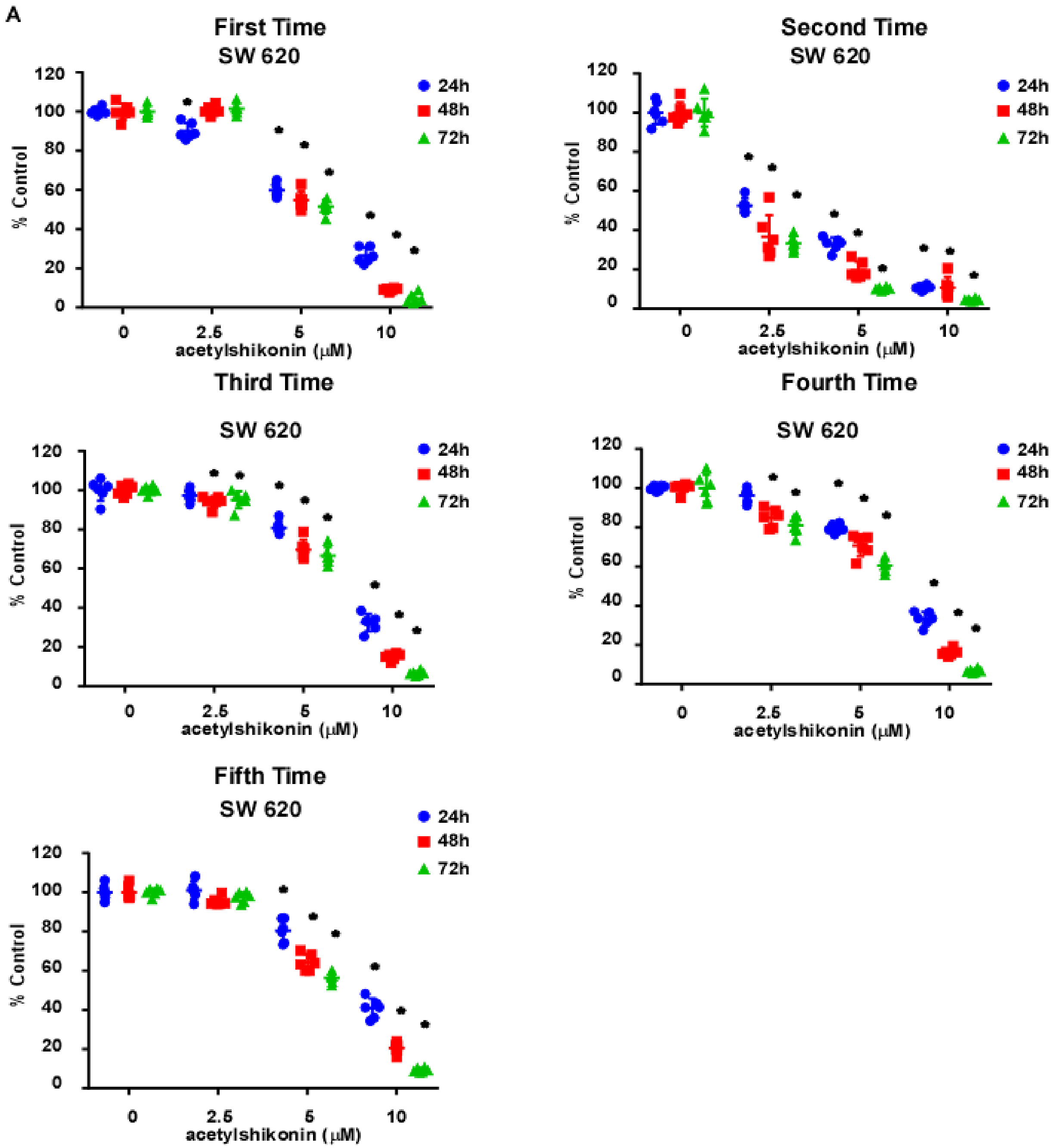

A
First Time
SW 620
Second Time
SW 620
Third Time
SW 620
Fourth Time
SW 620
Fifth Time
SW 620
24h
48h
72h
% Control
acetylshikonin (μM)
0
2.5
5
10

Figure 2

A

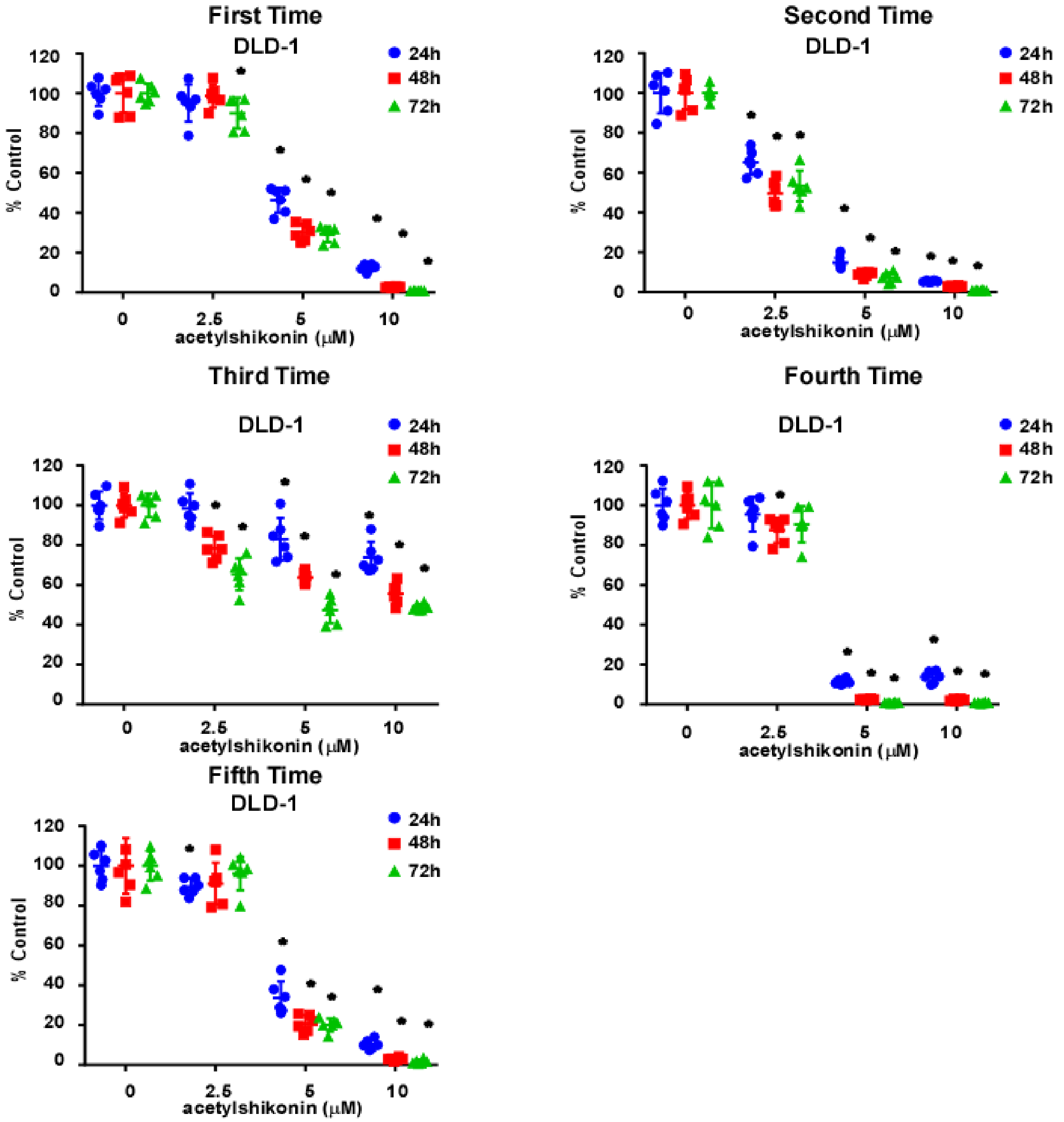

First Time
DLD-1
Second Time
DLD-1
Third Time
DLD-1
Fourth Time
DLD-1
Fifth Time
DLD-1
24h
48h
72h
% Control
120
100
80
60
40
20
0
0
2.5
5
10
acetylshikonin (μM)

Figure 2

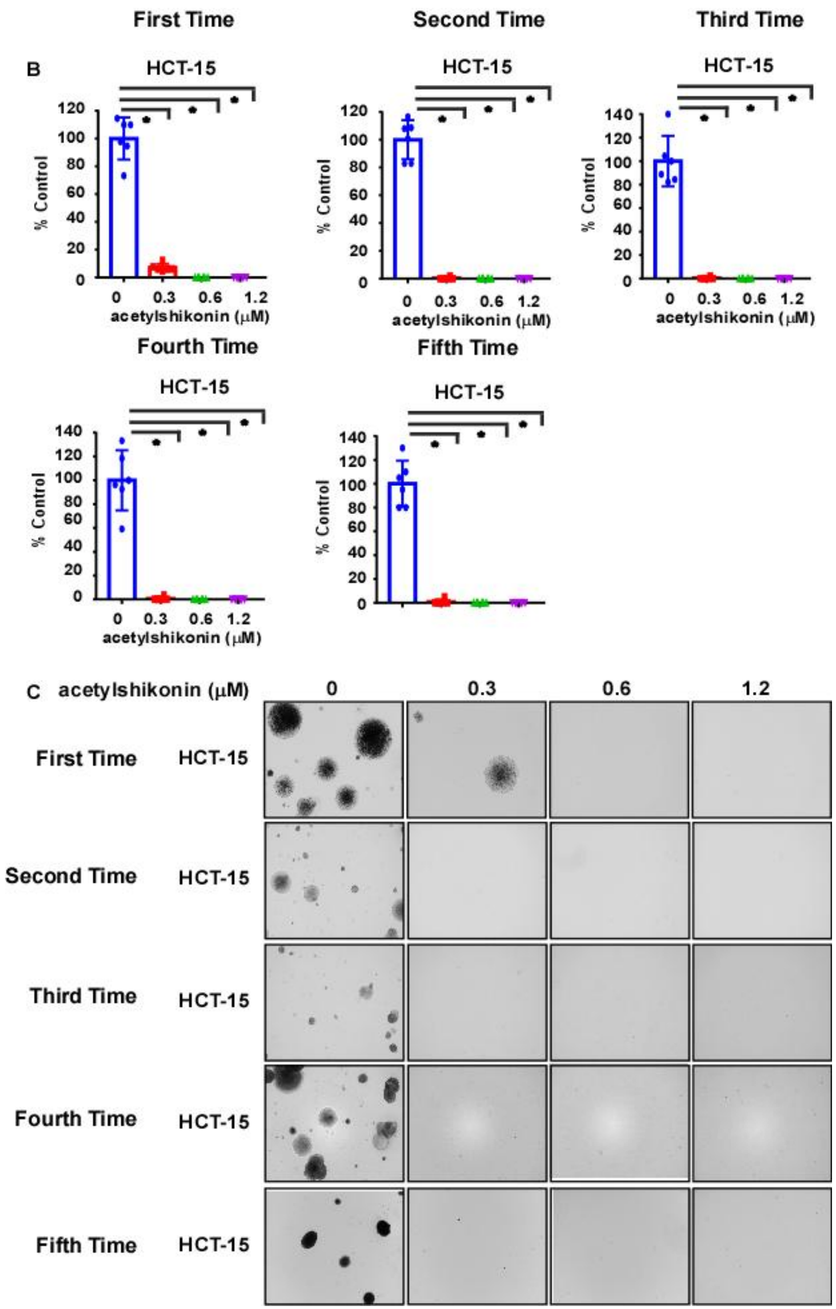

First Time
Second Time
Third Time
B
HCT-15
% Control
acetylshikonin (μM)
0
0.3
0.6
1.2
Fourth Time
Fifth Time
C
acetylshikonin (μM)
First Time
Second Time
Third Time
Fourth Time
Fifth Time

Figure 2

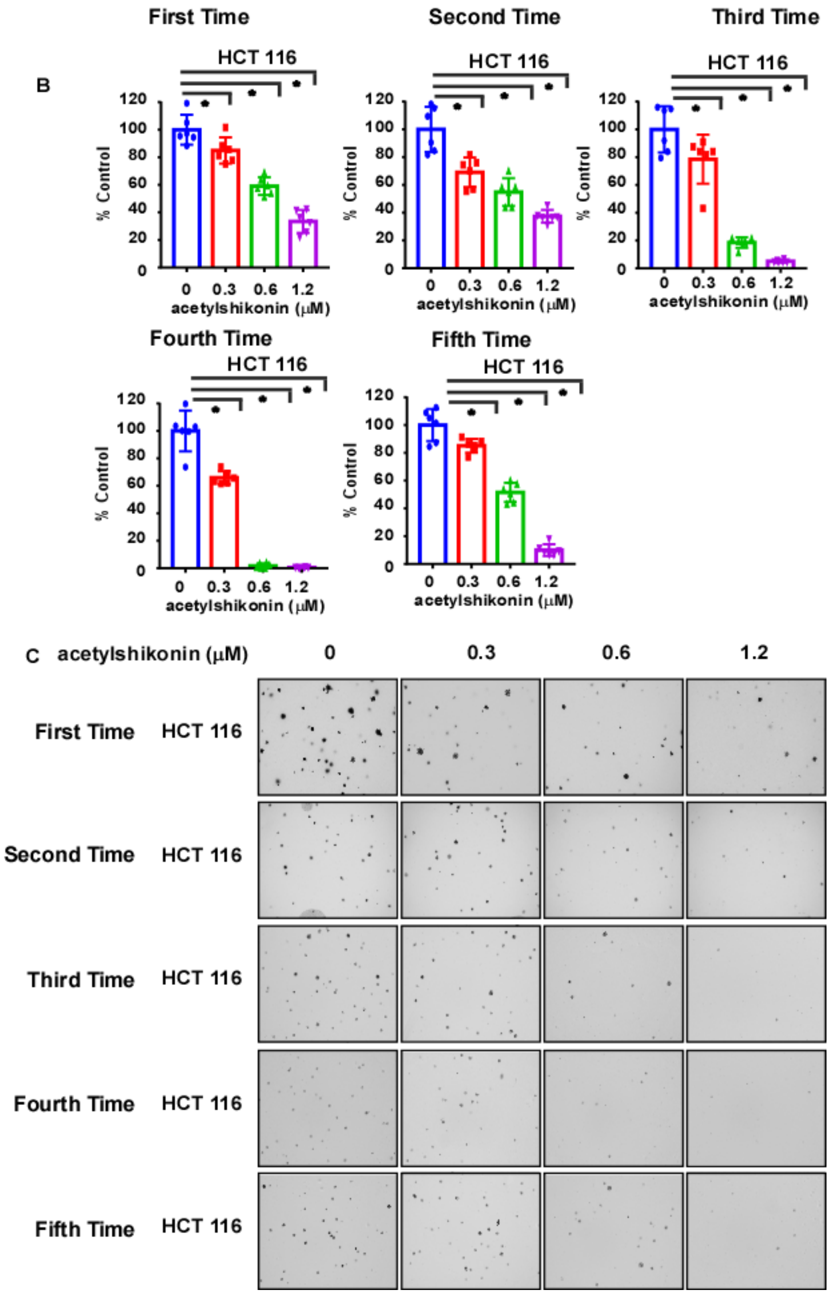

B
First Time
Second Time
Third Time
Fourth Time
Fifth Time
HCT 116
% Control
acetylshikonin (μM)
0
0.3
0.6
1.2
C
acetylshikonin (μM)
First Time HCT 116
Second Time HCT 116
Third Time HCT 116
Fourth Time HCT 116
Fifth Time HCT 116

Figure 2

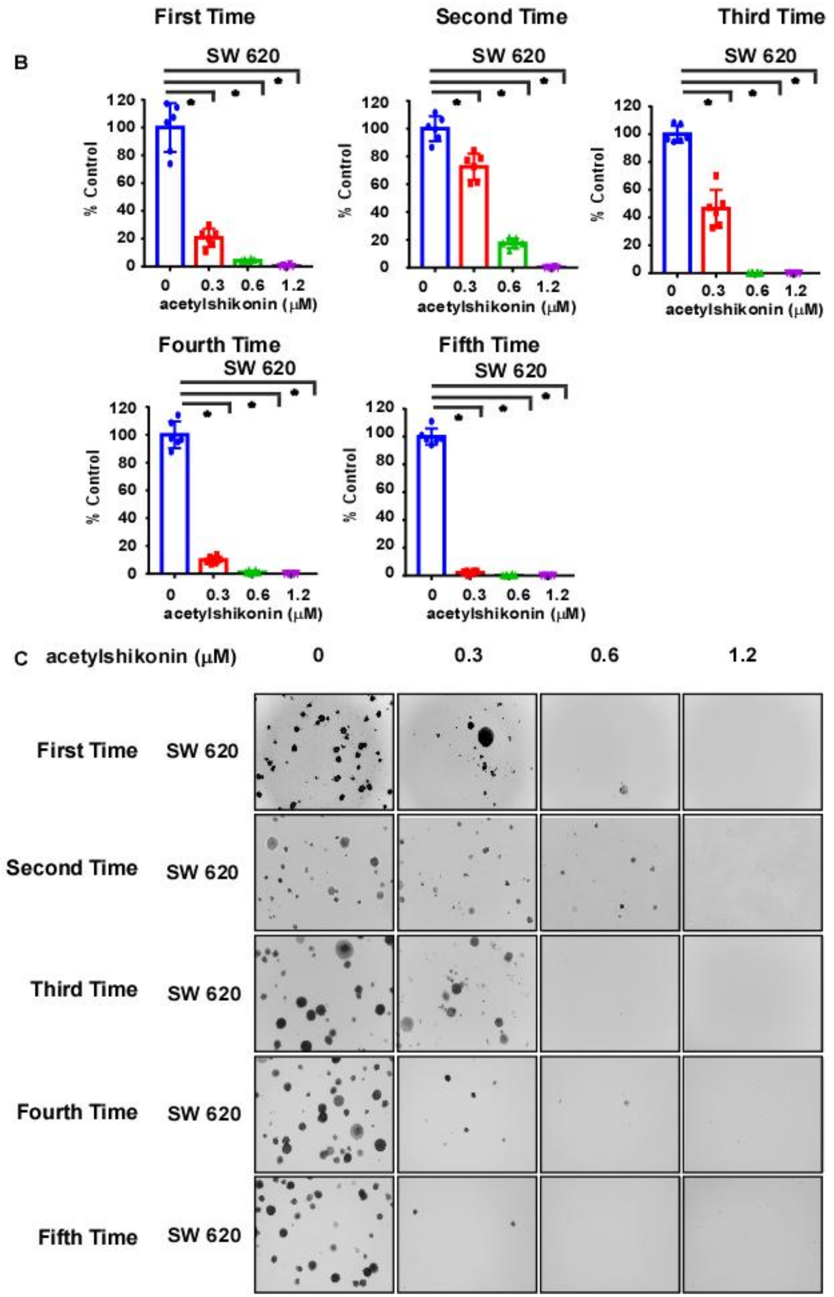
B
First Time
Second Time
Third Time
Fourth Time
Fifth Time
SW 620
% Control
acetylshikonin (μM)
0 0.3 0.6 1.2
C acetylshikonin (μM)
0
0.3
0.6
1.2
First Time SW 620
Second Time SW 620
Third Time SW 620
Fourth Time SW 620
Fifth Time SW 620

Figure 2

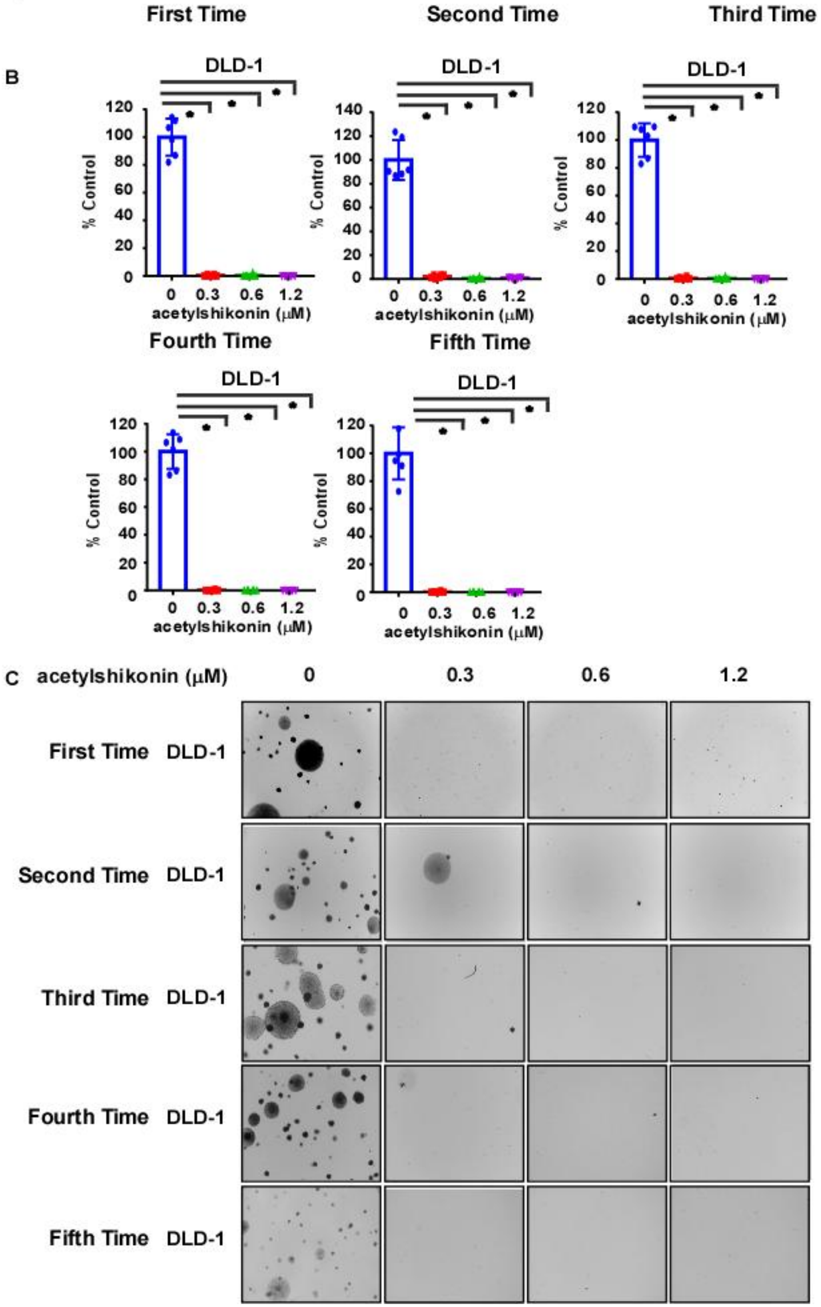

First Time
Second Time
Third Time
B
DLD-1
% Control
acetylshikonin (μM)
0 0.3 0.6 1.2
Fourth Time
Fifth Time
C acetylshikonin (μM)
0
0.3
0.6
1.2
First Time DLD-1
Second Time DLD-1
Third Time DLD-1
Fourth Time DLD-1
Fifth Time DLD-1

Figure 3

A

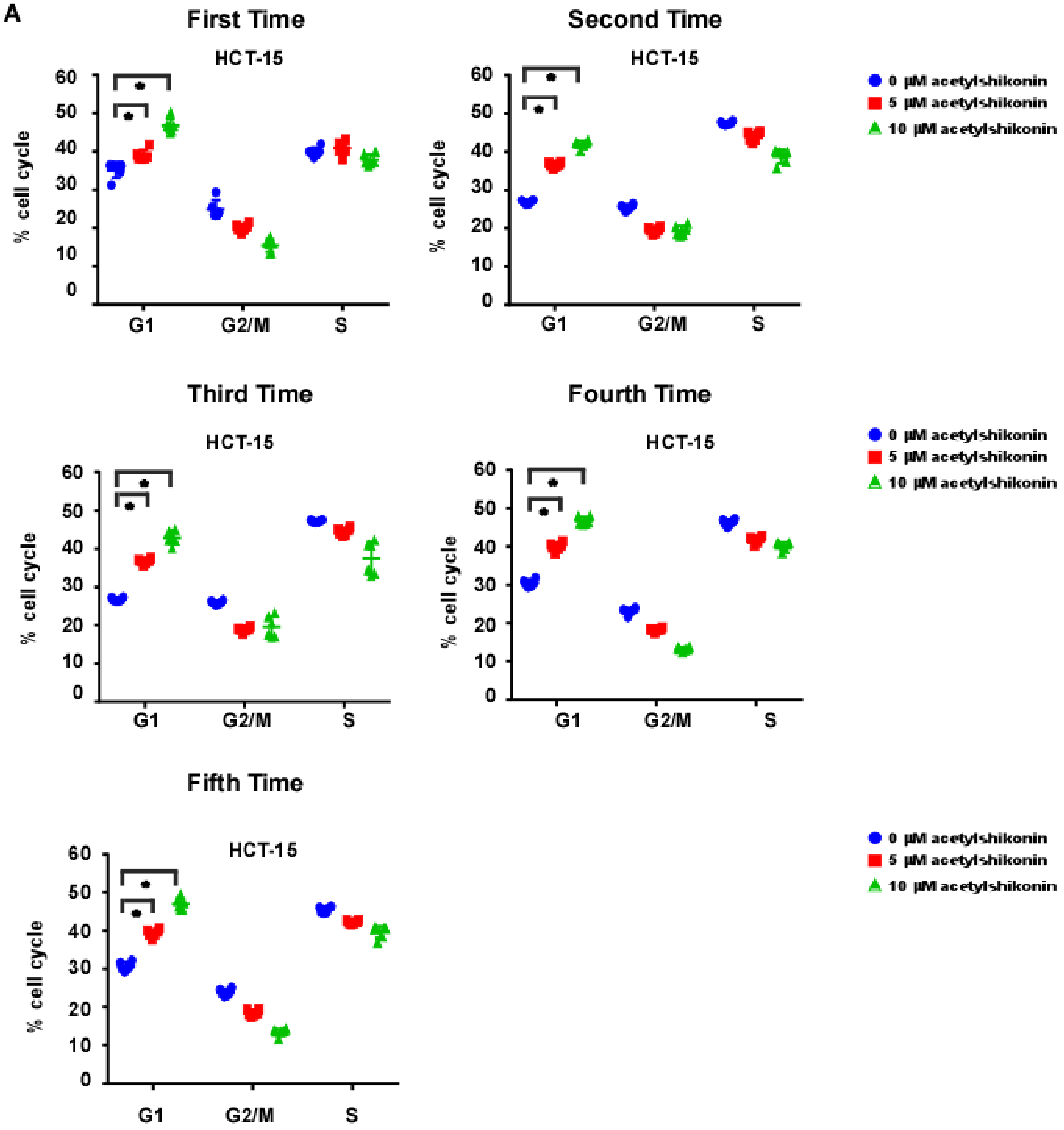
First Time
Second Time
Third Time
Fourth Time
Fifth Time
HCT-15
% cell cycle
0
10
20
30
40
50
60
G1
G2/M
S
*
0 μM acetylshikonin
5 μM acetylshikonin
10 μM acetylshikonin

Figure 3

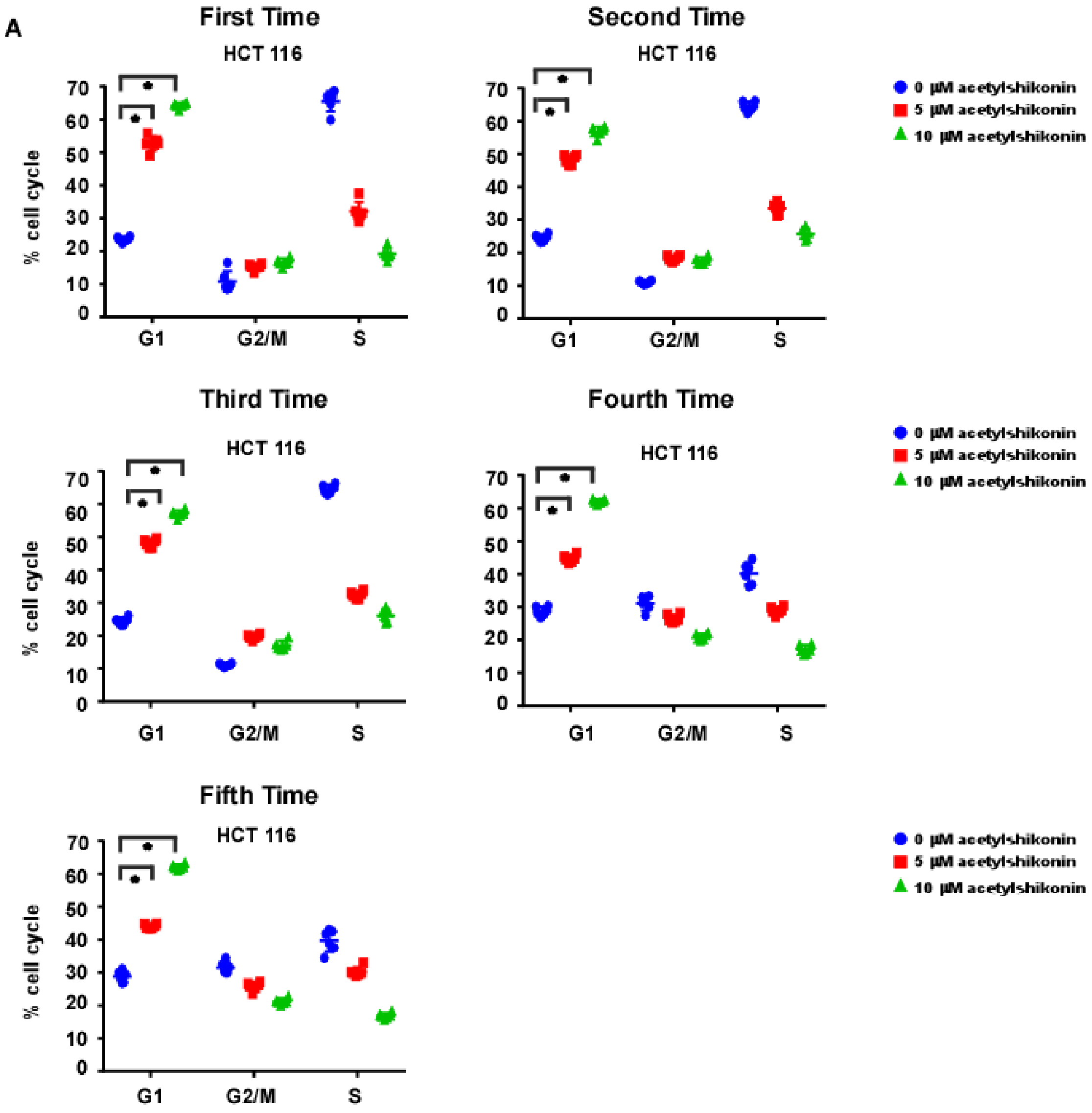
A
First Time
HCT 116
Second Time
HCT 116
Third Time
HCT 116
Fourth Time
HCT 116
Fifth Time
HCT 116
% cell cycle
G1
G2/M
S
0 µM acetylshikonin
5 µM acetylshikonin
10 µM acetylshikonin

Figure 3

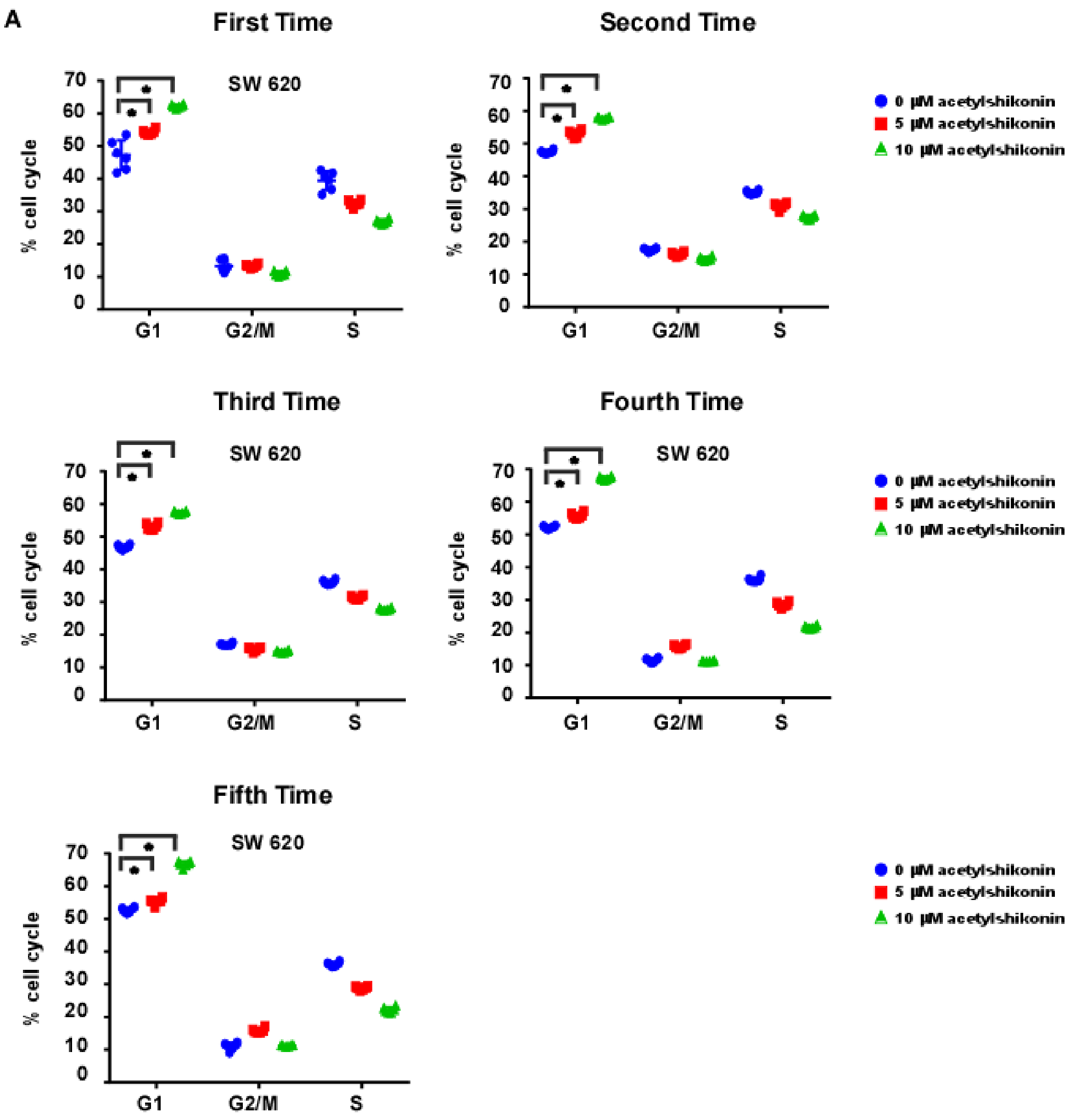
A
First Time
Second Time
Third Time
Fourth Time
Fifth Time
SW 620
% cell cycle
G1
G2/M
S
0 μM acetylshikonin
5 μM acetylshikonin
10 μM acetylshikonin

Figure 3

A

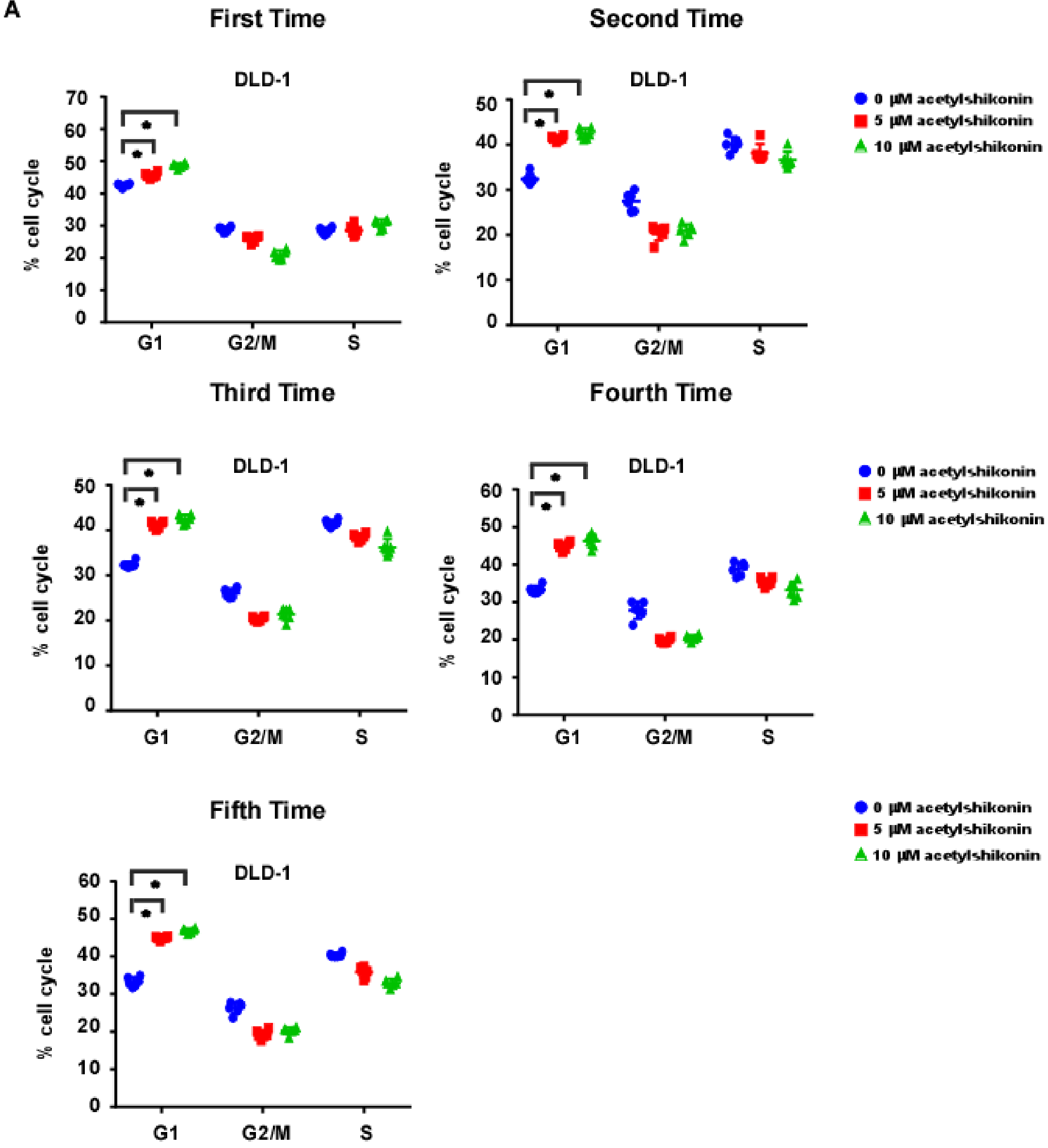
First Time
Second Time
Third Time
Fourth Time
Fifth Time
DLD-1
% cell cycle
G1
G2/M
S
0 μM acetylshikonin
5 μM acetylshikonin
10 μM acetylshikonin

Figure 3

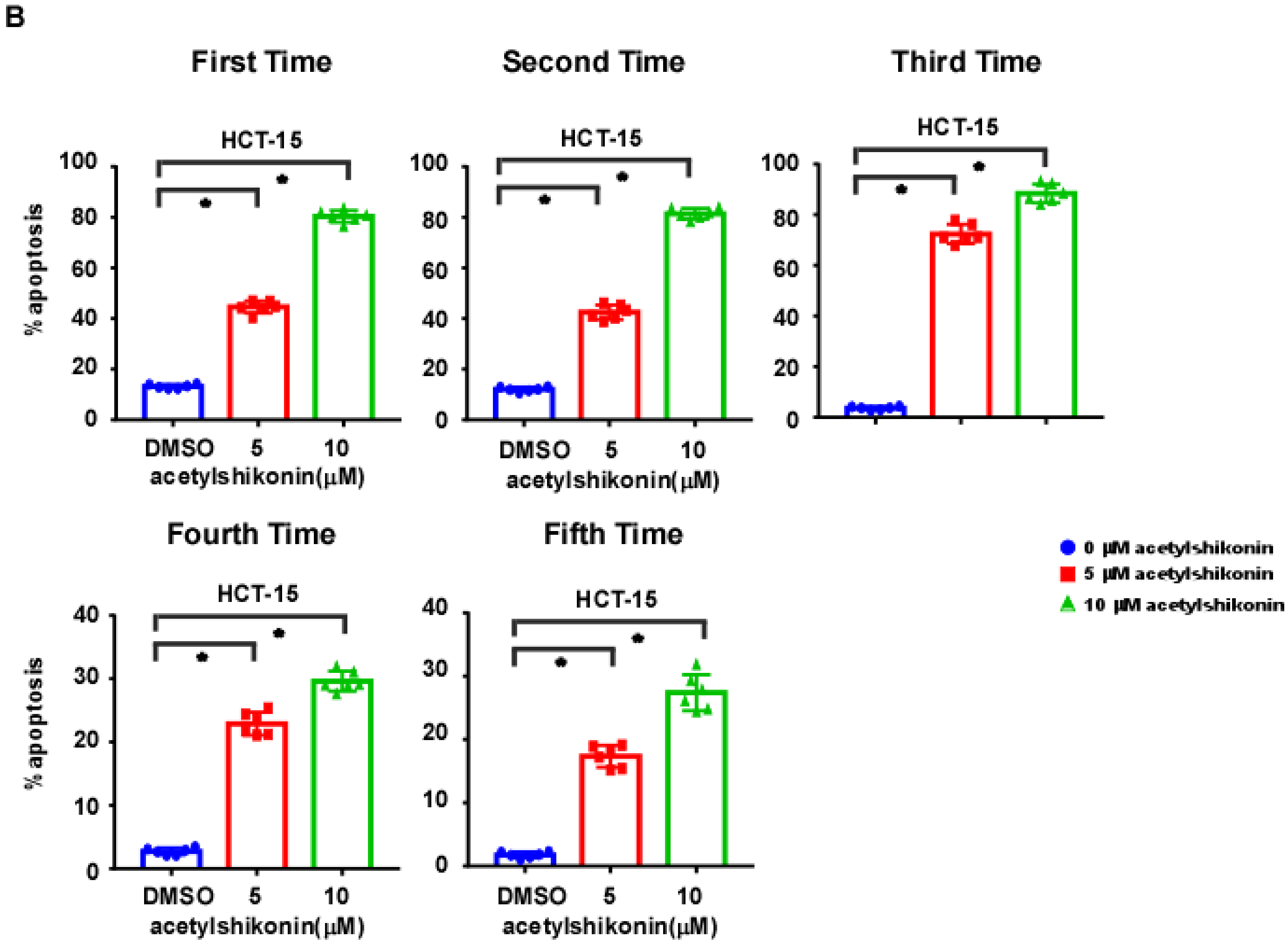
B
First Time
Second Time
Third Time
Fourth Time
Fifth Time
HCT-15
% apoptosis
DMSO
5
10
acetylshikonin(μM)
0 μM acetylshikonin
5 μM acetylshikonin
10 μM acetylshikonin

Figure 3

B

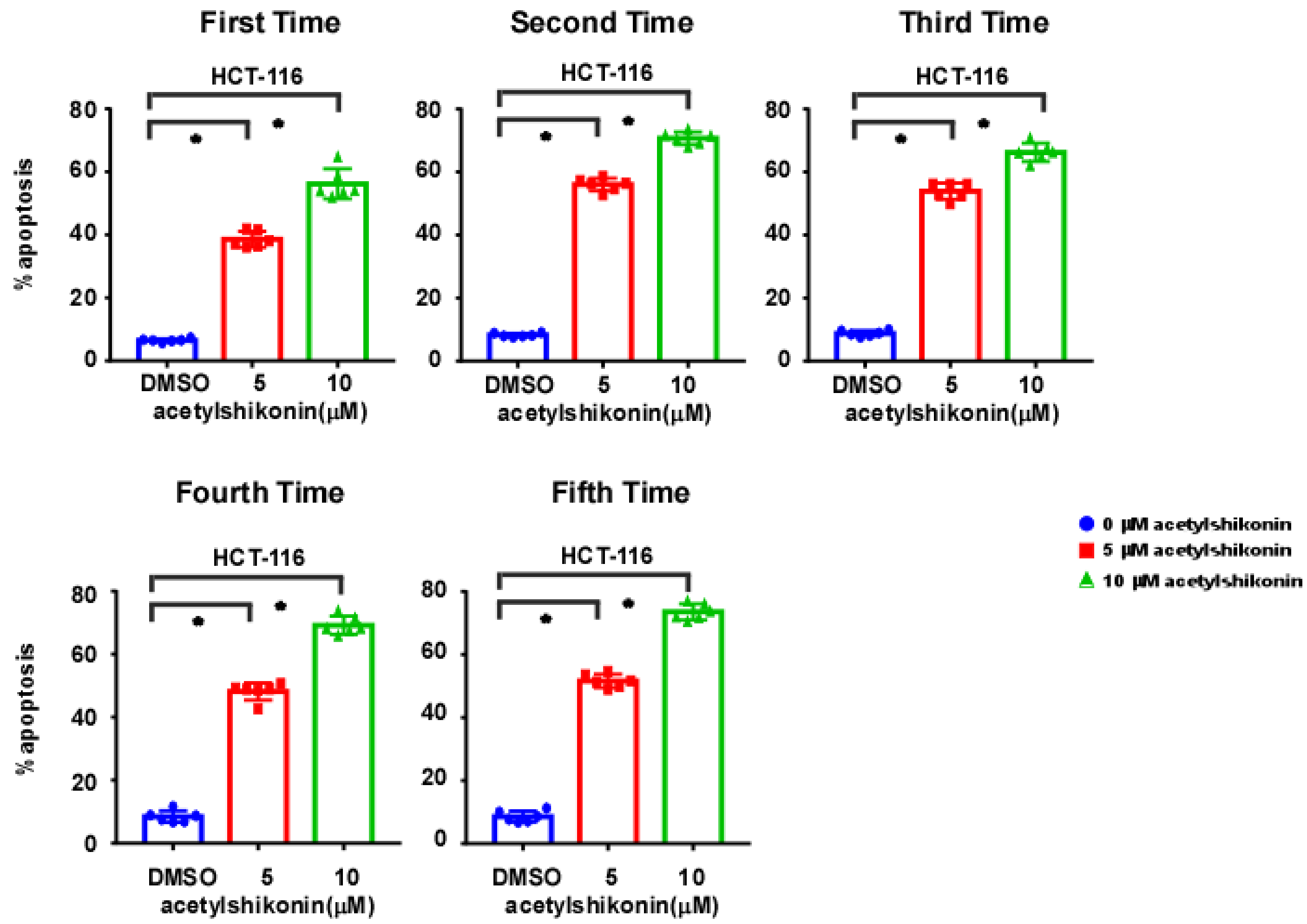
First Time
Second Time
Third Time
Fourth Time
Fifth Time
HCT-116
% apoptosis
80
60
40
20
0
DMSO
5
10
acetylshikonin(μM)
0 μM acetylshikonin
5 μM acetylshikonin
10 μM acetylshikonin

Figure 3

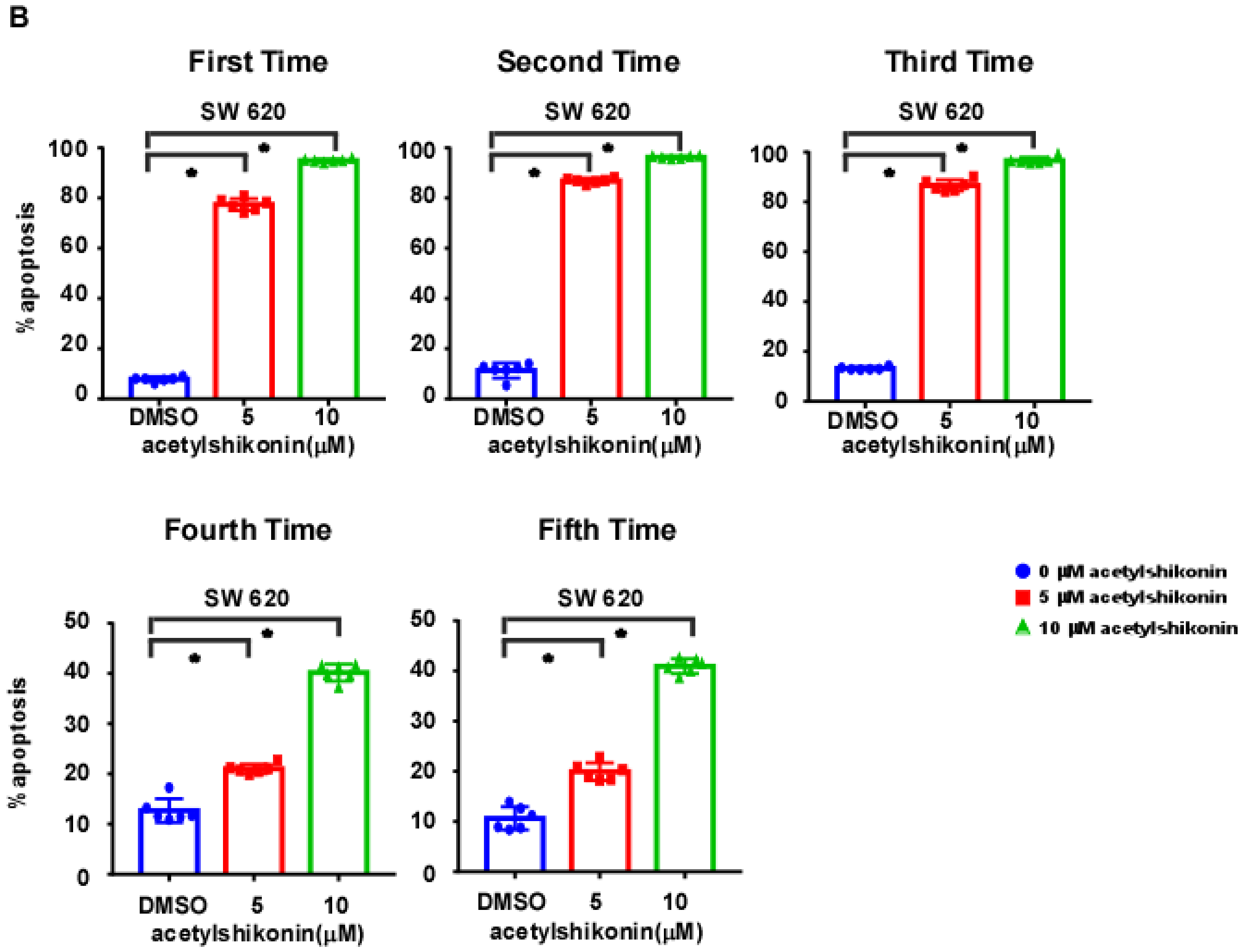

B
First Time
SW 620
Second Time
SW 620
Third Time
SW 620
Fourth Time
SW 620
Fifth Time
SW 620
% apoptosis
DMSO
5
10
acetylshikonin(μM)
0 μM acetylshikonin
5 μM acetylshikonin
10 μM acetylshikonin

Figure 3

B

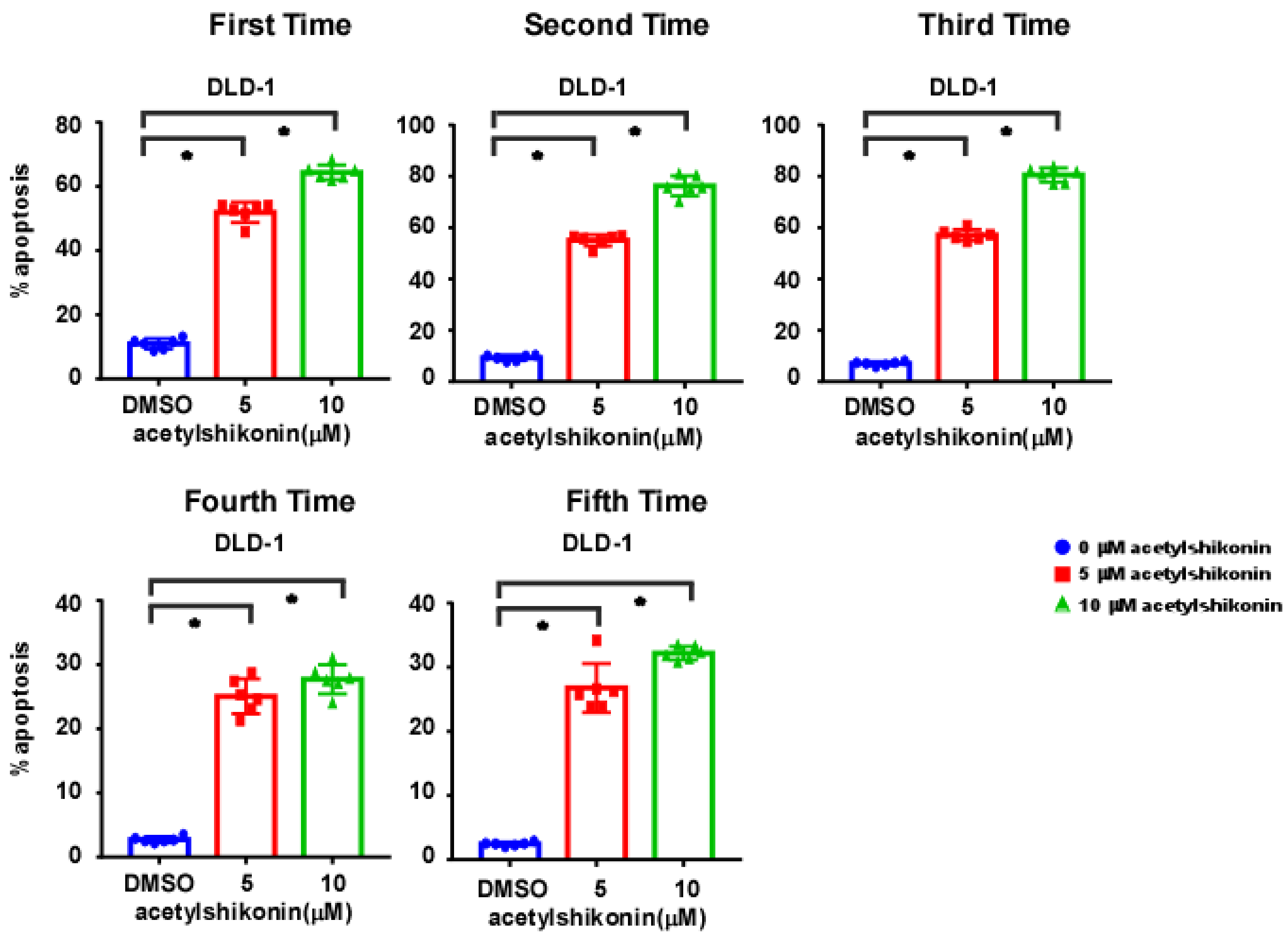

First Time
DLD-1
% apoptosis
DMSO
5
10
acetylshikonin(μM)
Second Time
DLD-1
Third Time
DLD-1
Fourth Time
DLD-1
Fifth Time
DLD-1
0 μM acetylshikonin
5 μM acetylshikonin
10 μM acetylshikonin

Figure 4

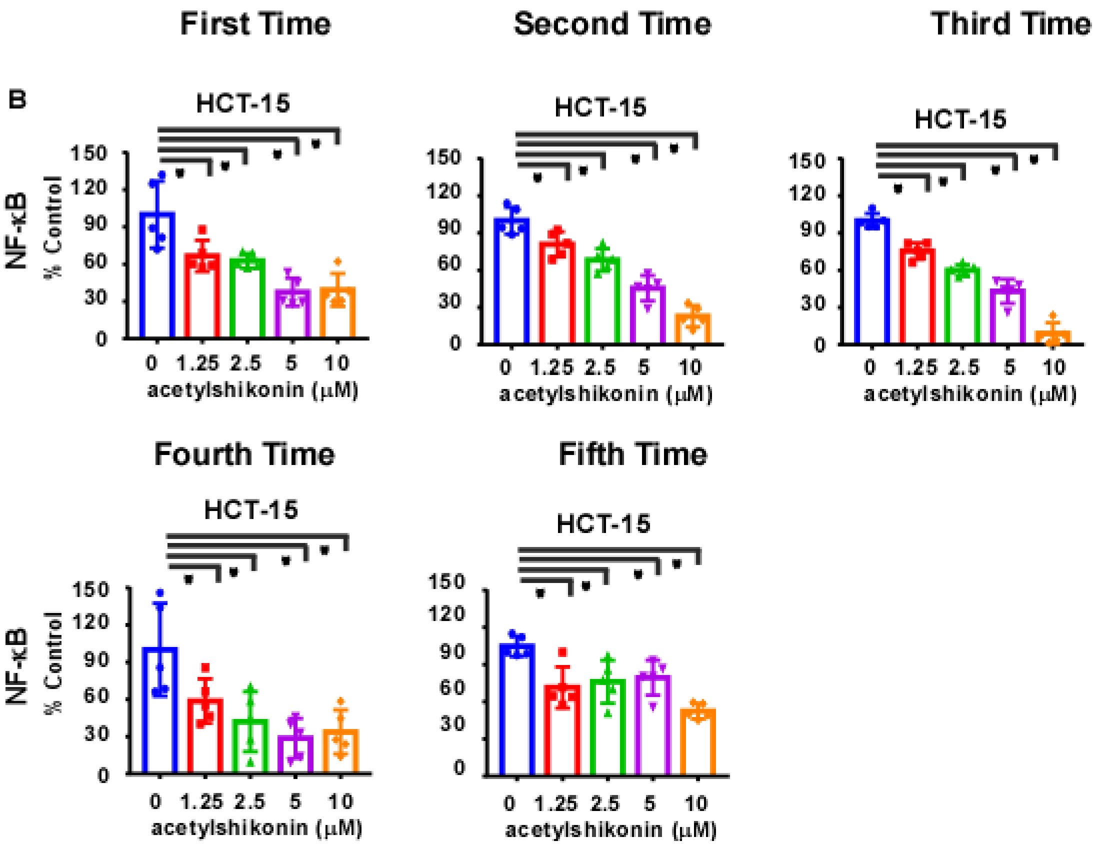
First Time
Second Time
Third Time
B
HCT-15
NF-κB
% Control
150
120
90
60
30
0
0 1.25 2.5 5 10
acetylshikonin (μM)
Fourth Time
Fifth Time

Figure 4

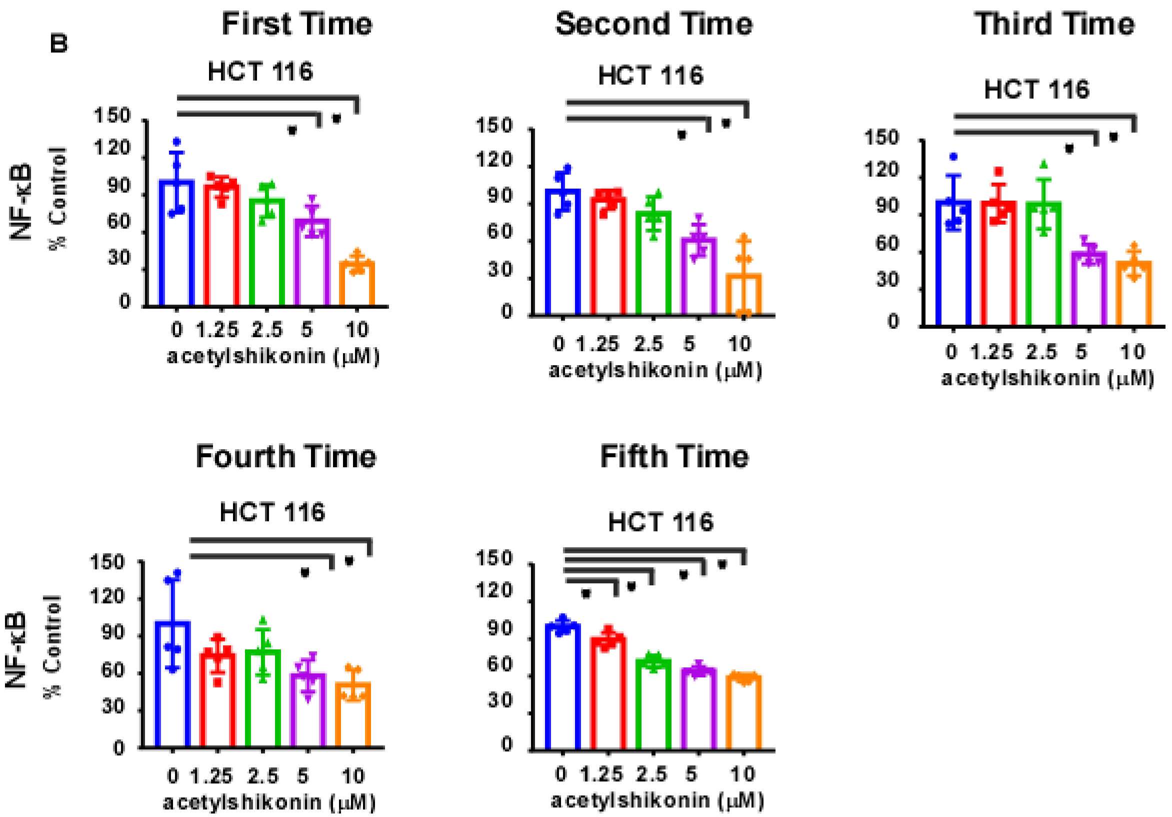
B
First Time
Second Time
Third Time
Fourth Time
Fifth Time
HCT 116
NF-κB
% Control
acetylshikonin (μM)
0 1.25 2.5 5 10
0 30 60 90 120 150

Figure 4

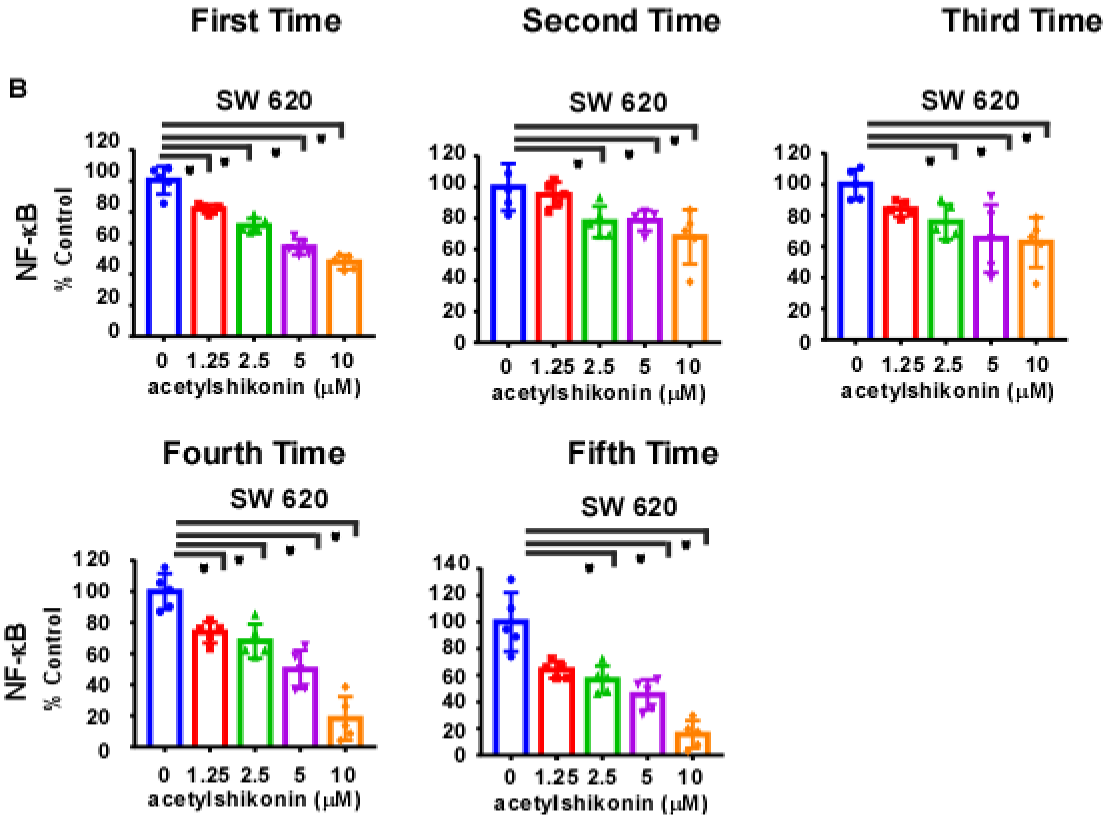
First Time
Second Time
Third Time
B
SW 620
NF-κB
% Control
0
20
40
60
80
100
120
140
0 1.25 2.5 5 10
acetylshikonin (μM)
Fourth Time
Fifth Time

Figure 4

B

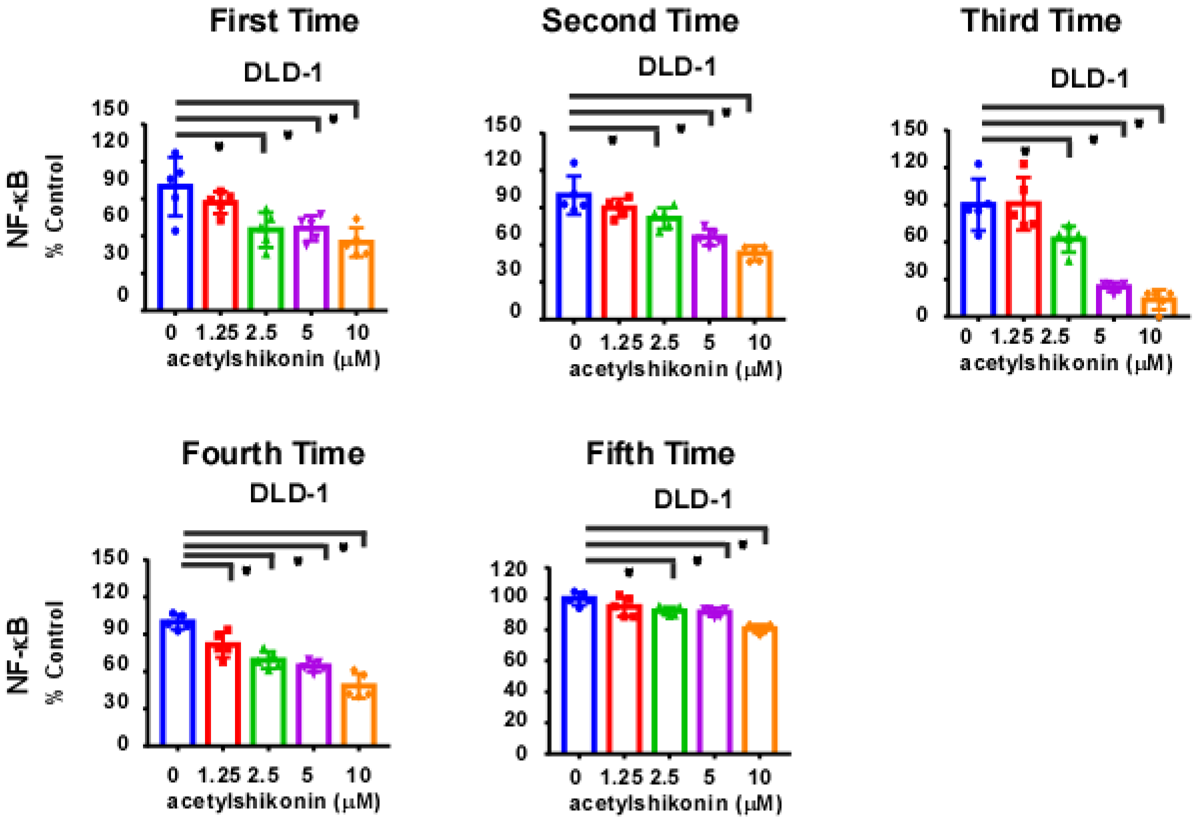
First Time
DLD-1
NF-κB
% Control
0
30
60
90
120
150
0 1.25 2.5 5 10
acetylshikonin (μM)
Second Time
DLD-1
150
120
90
60
30
0
0 1.25 2.5 5 10
acetylshikonin (μM)
Third Time
DLD-1
150
120
90
60
30
0
0 1.25 2.5 5 10
acetylshikonin (μM)
Fourth Time
DLD-1
NF-κB
% Control
150
120
90
60
30
0
0 1.25 2.5 5 10
acetylshikonin (μM)
Fifth Time
DLD-1
120
100
80
60
40
20
0
0 1.25 2.5 5 10
acetylshikonin (μM)

Figure 5

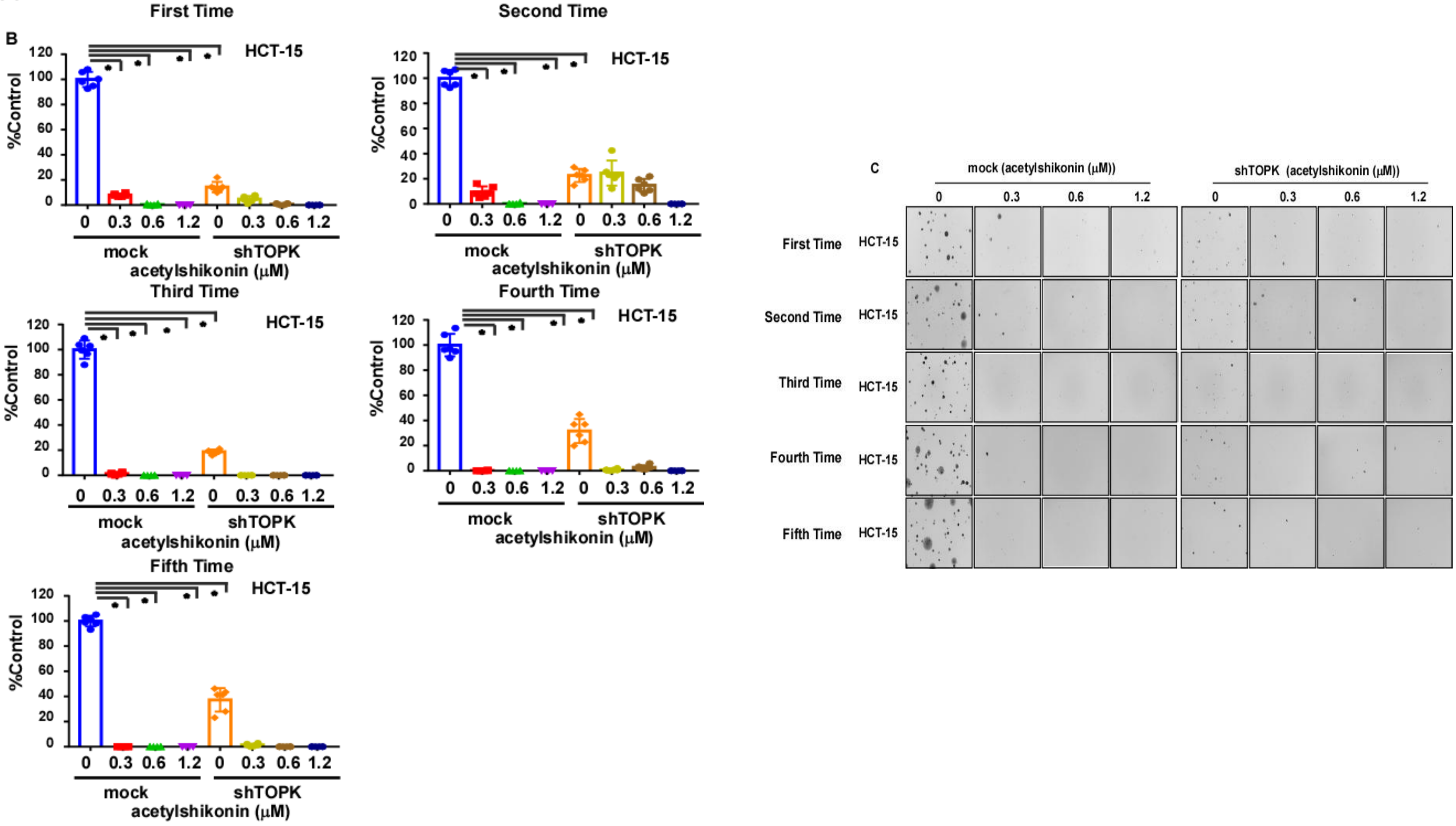
B
First Time
Second Time
Third Time
Fourth Time
Fifth Time
HCT-15
%Control
0 0.3 0.6 1.2 0 0.3 0.6 1.2
mock
shTOPK
acetylshikonin (μM)
C
mock (acetylshikonin (μM))
shTOPK (acetylshikonin (μM))
0 0.3 0.6 1.2 0 0.3 0.6 1.2
First Time HCT-15
Second Time HCT-15
Third Time HCT-15
Fourth Time HCT-15
Fifth Time HCT-15

Figure 5

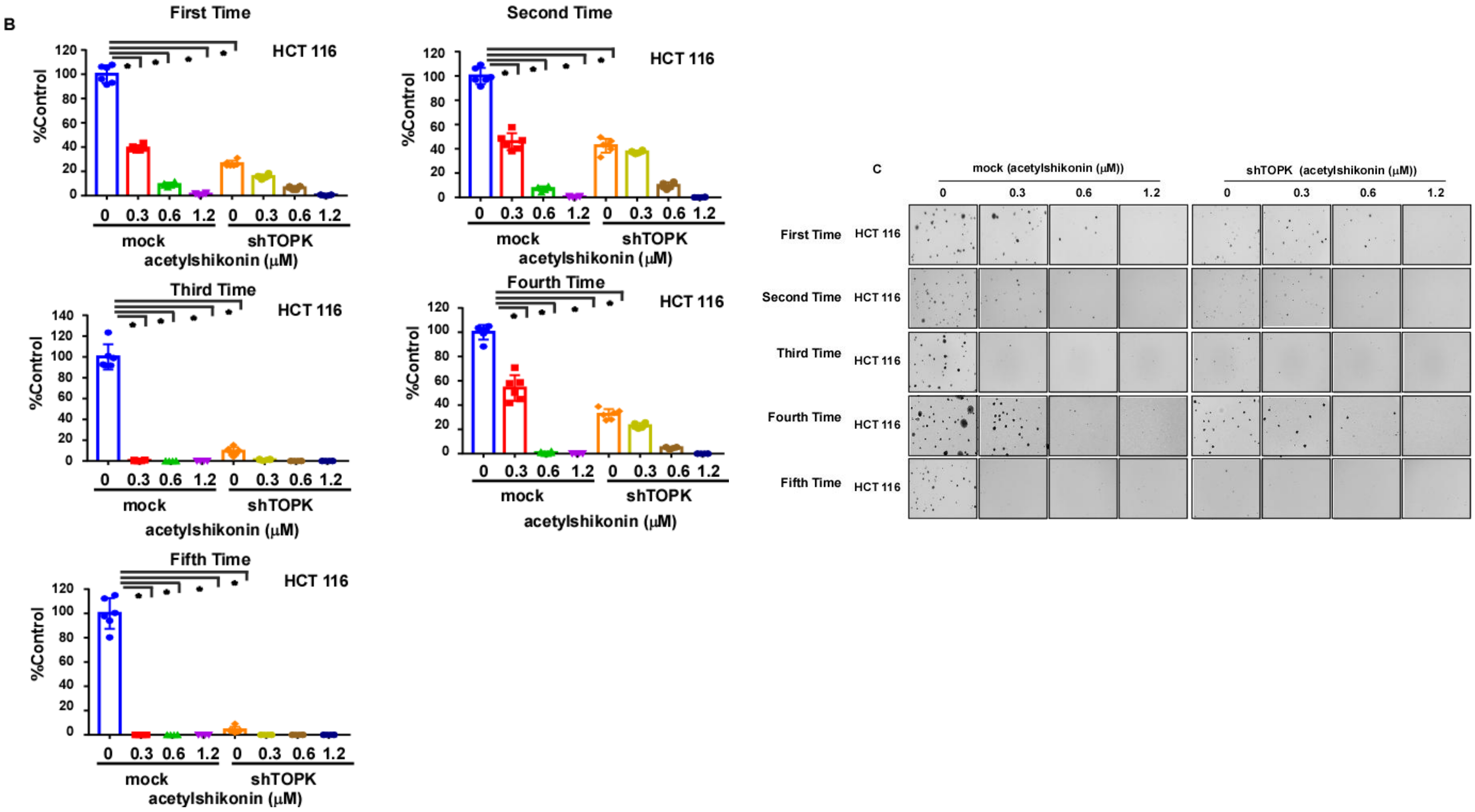
B
First Time
HCT 116
%Control
0 0.3 0.6 1.2 0 0.3 0.6 1.2
mock shTOPK
acetylshikonin (μM)
Second Time
HCT 116
Third Time
HCT 116
Fourth Time
HCT 116
Fifth Time
HCT 116
C
mock (acetylshikonin (μM))
shTOPK (acetylshikonin (μM))
First Time HCT 116
Second Time HCT 116
Third Time HCT 116
Fourth Time HCT 116
Fifth Time HCT 116

Figure 5

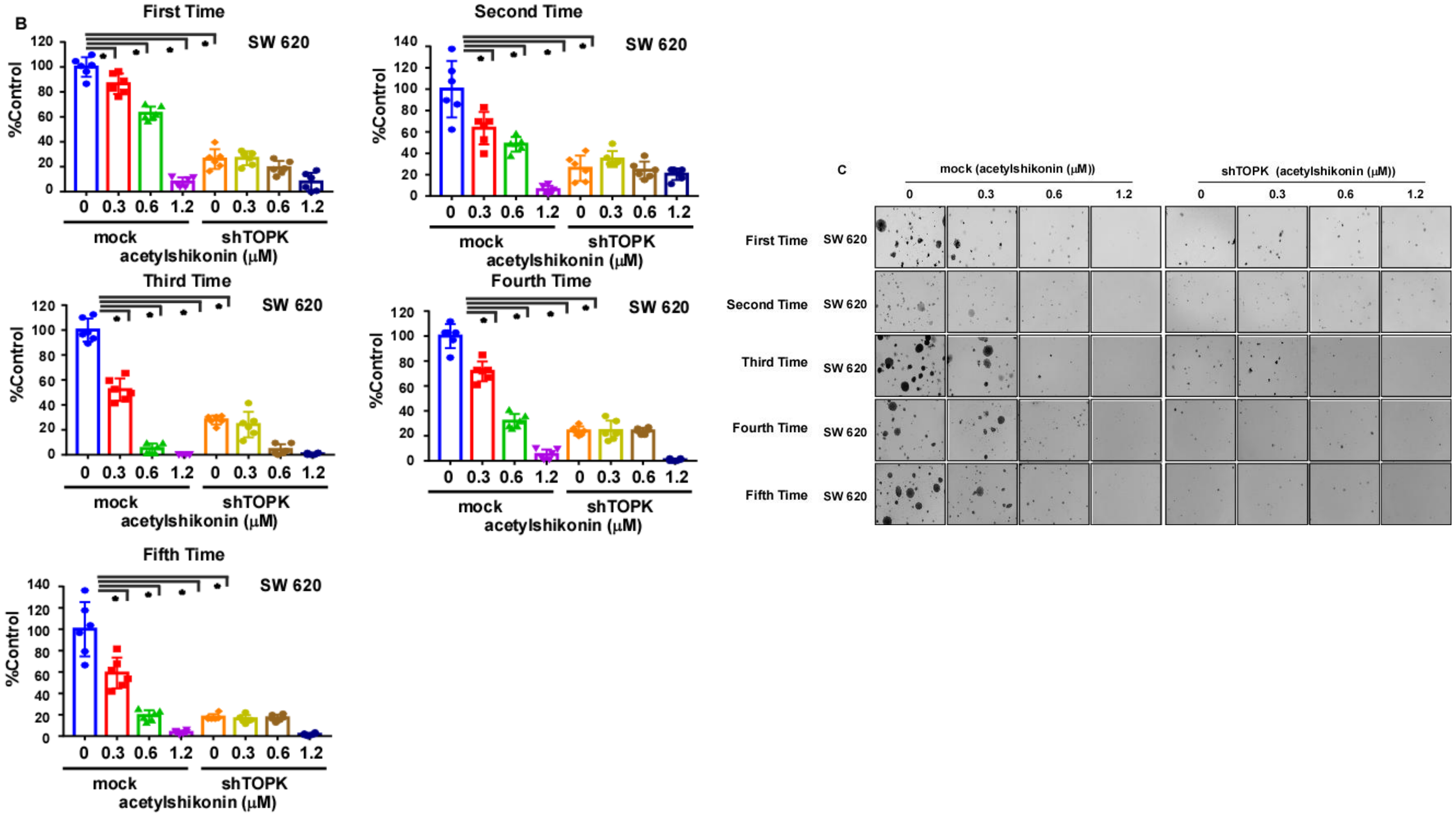
B
First Time
SW 620
Second Time
Third Time
Fourth Time
Fifth Time
%Control
0 0.3 0.6 1.2 0 0.3 0.6 1.2
mock
shTOPK
acetylshikonin (μM)
C
mock (acetylshikonin (μM))
shTOPK (acetylshikonin (μM))
First Time SW 620
Second Time SW 620
Third Time SW 620
Fourth Time SW 620
Fifth Time SW 620

Figure 5

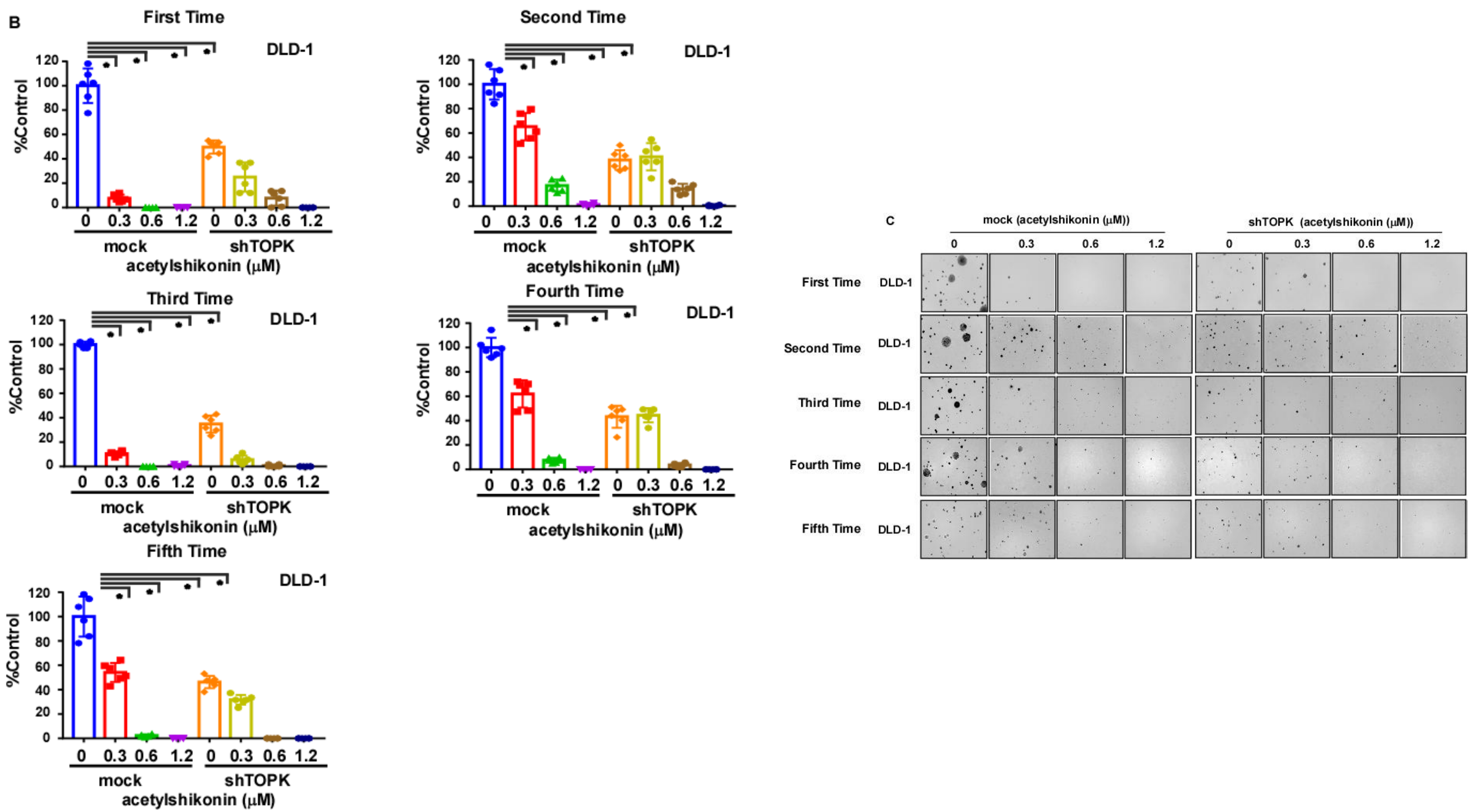
B
First Time
DLD-1
Second Time
DLD-1
Third Time
DLD-1
Fourth Time
DLD-1
Fifth Time
DLD-1
%Control
120
100
80
60
40
20
0
0 0.3 0.6 1.2 0 0.3 0.6 1.2
mock
shTOPK
acetylshikonin (μM)
C
mock (acetylshikonin (μM))
shTOPK (acetylshikonin (μM))
0
0.3
0.6
1.2
First Time DLD-1
Second Time DLD-1
Third Time DLD-1
Fourth Time DLD-1
Fifth Time DLD-1

Figure 6

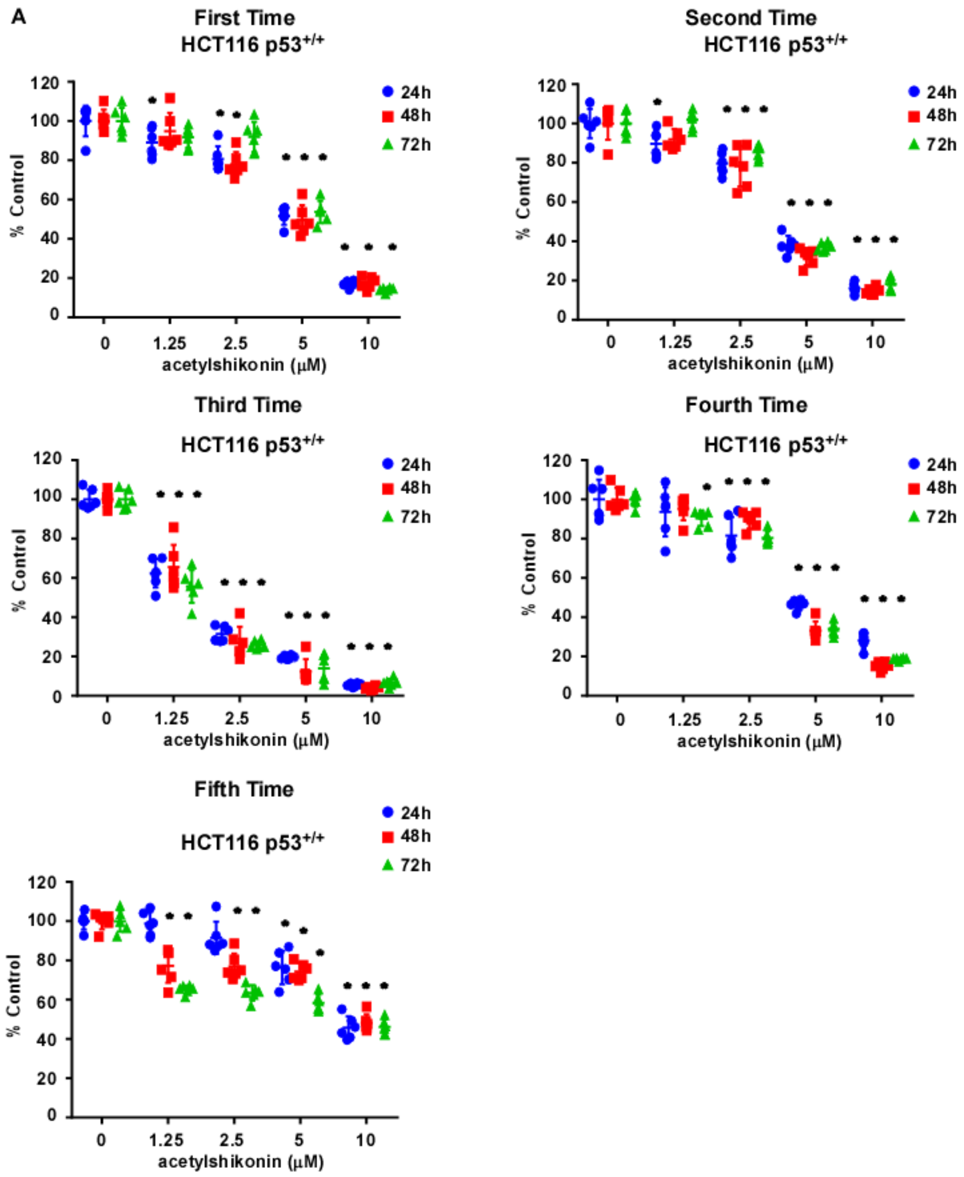
A
First Time
HCT116 p53+/+
Second Time
HCT116 p53+/+
Third Time
HCT116 p53+/+
Fourth Time
HCT116 p53+/+
Fifth Time
HCT116 p53+/+
24h
48h
72h
% Control
0
20
40
60
80
100
120
0
1.25
2.5
5
10
acetylshikonin (μM)

Figure 6

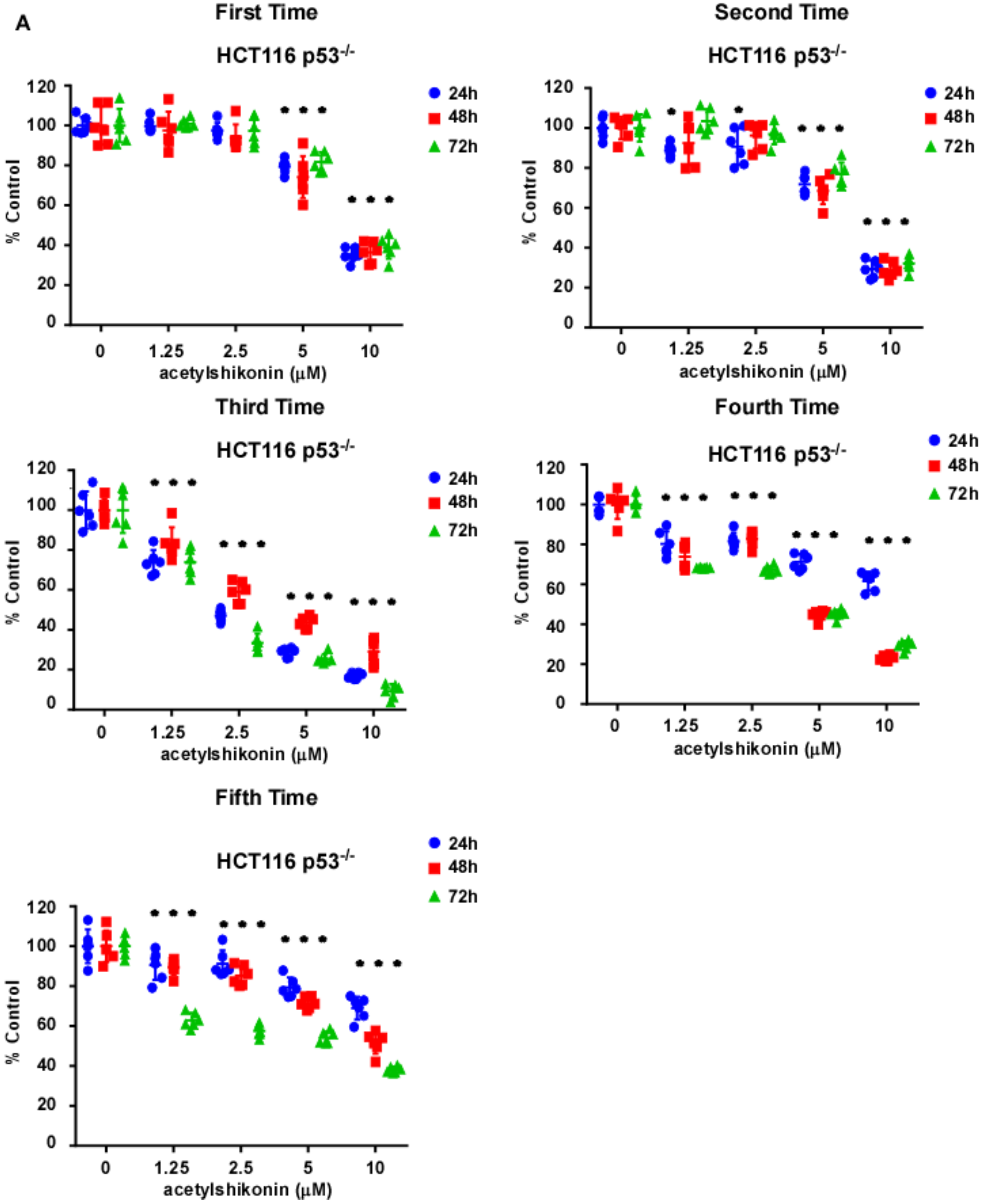
A
First Time
HCT116 p53-/-
Second Time
HCT116 p53-/-
Third Time
HCT116 p53-/-
Fourth Time
HCT116 p53-/-
Fifth Time
HCT116 p53-/-
% Control
120
100
80
60
40
20
0
0
1.25
2.5
5
10
acetylshikonin (μM)
24h
48h
72h
* * *

Figure 6

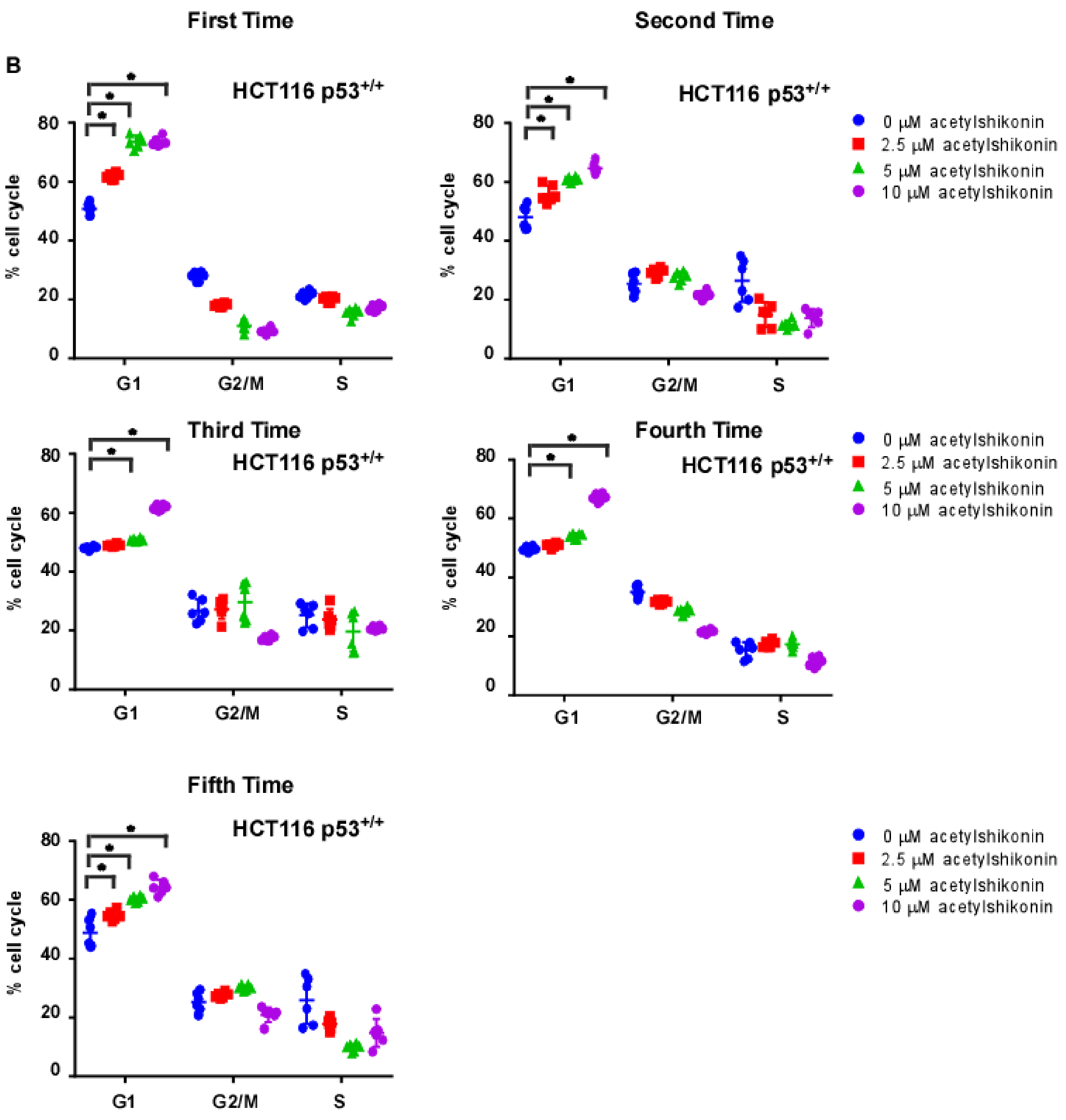

B
First Time
Second Time
Third Time
Fourth Time
Fifth Time
HCT116 p53+/+
% cell cycle
0
20
40
60
80
G1
G2/M
S
*
0 μM acetylshikonin
2.5 μM acetylshikonin
5 μM acetylshikonin
10 μM acetylshikonin

Figure 6

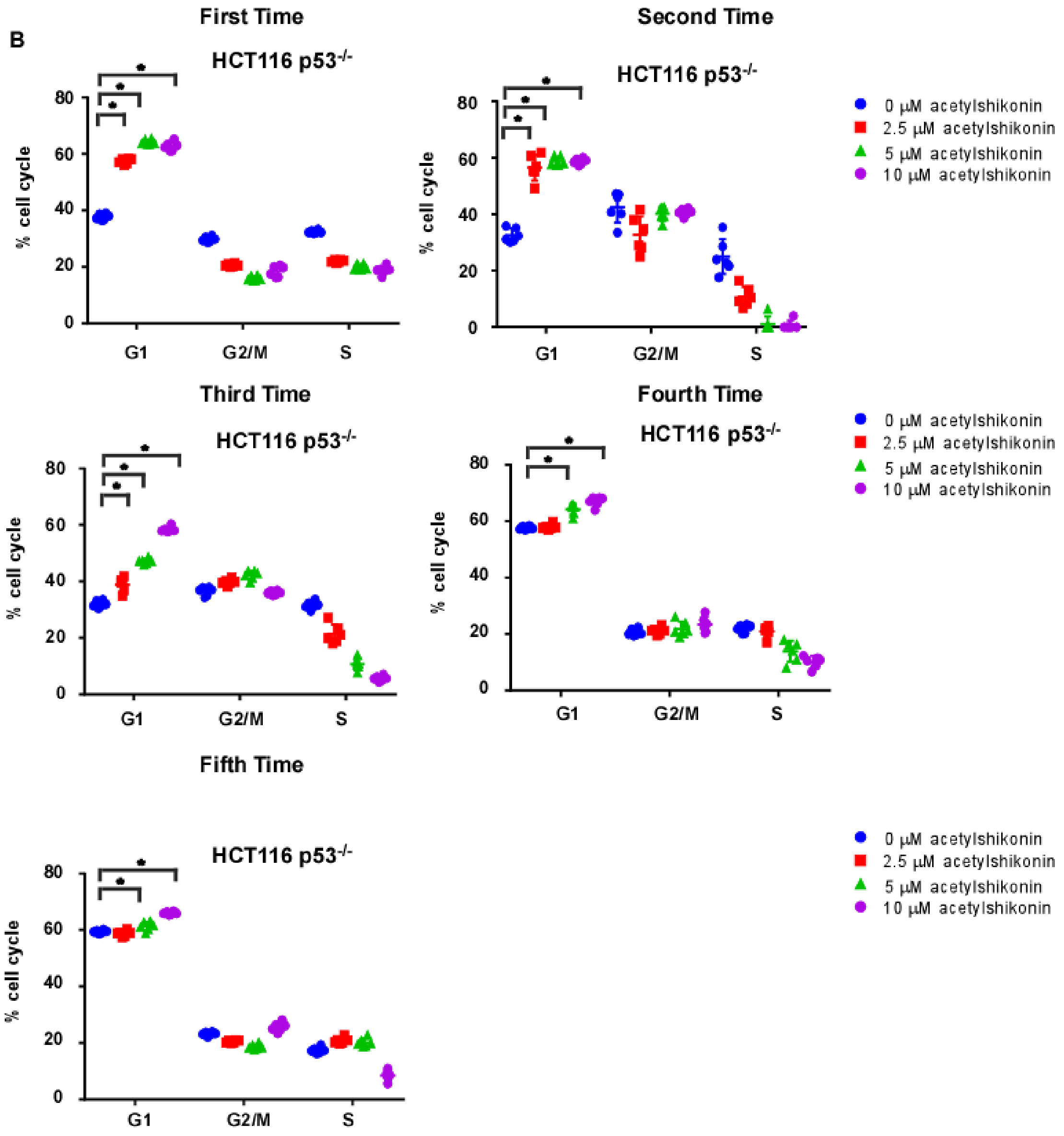
B
First Time
Second Time
Third Time
Fourth Time
Fifth Time
HCT116 p53-/-
% cell cycle
G1
G2/M
S
0 μM acetylshikonin
2.5 μM acetylshikonin
5 μM acetylshikonin
10 μM acetylshikonin

Figure 6

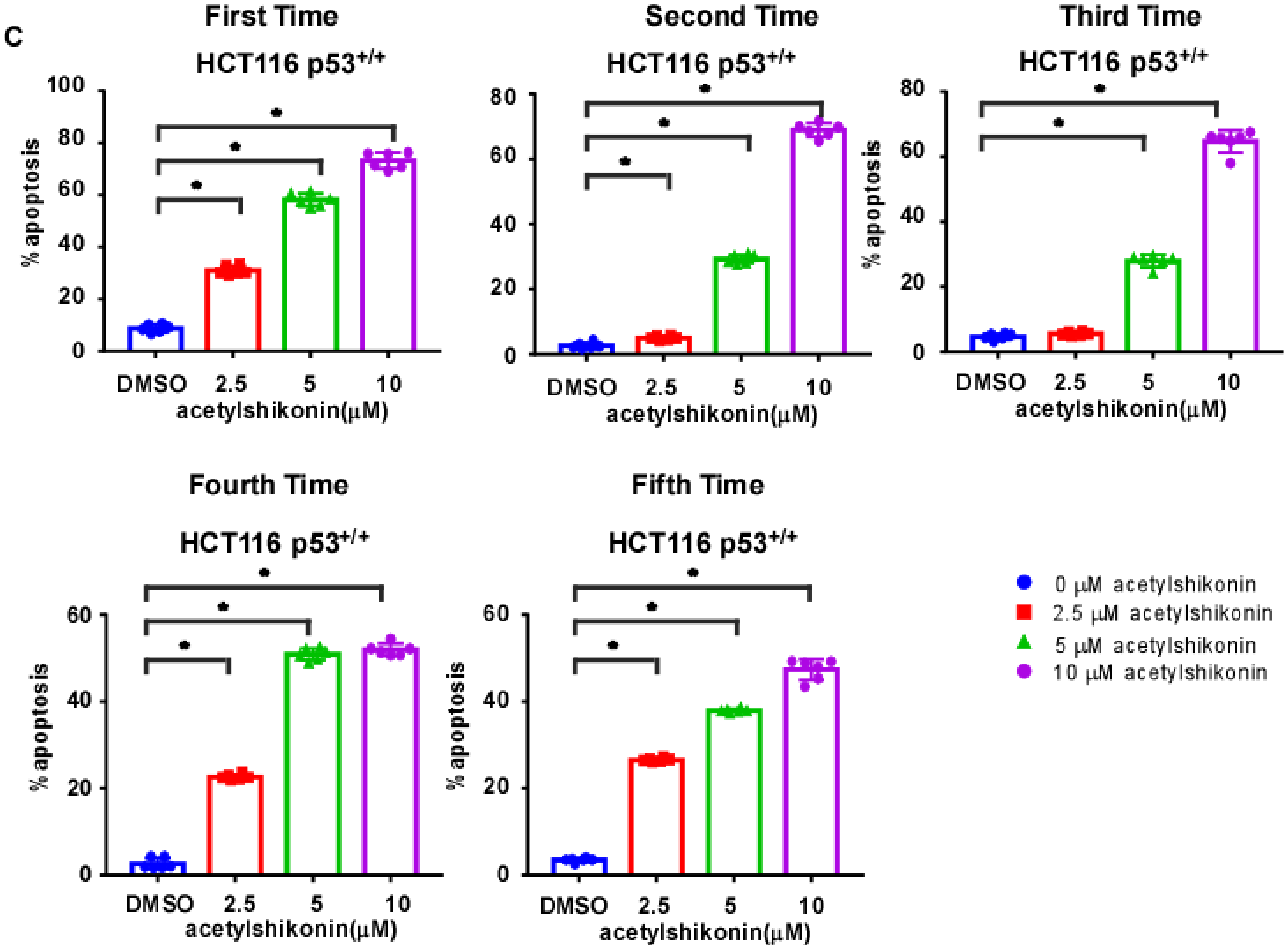
C
First Time
HCT116 p53+/+
Second Time
HCT116 p53+/+
Third Time
HCT116 p53+/+
Fourth Time
HCT116 p53+/+
Fifth Time
HCT116 p53+/+
% apoptosis
DMSO
2.5
5
10
acetylshikonin(μM)
0 μM acetylshikonin
2.5 μM acetylshikonin
5 μM acetylshikonin
10 μM acetylshikonin

Figure 6

C

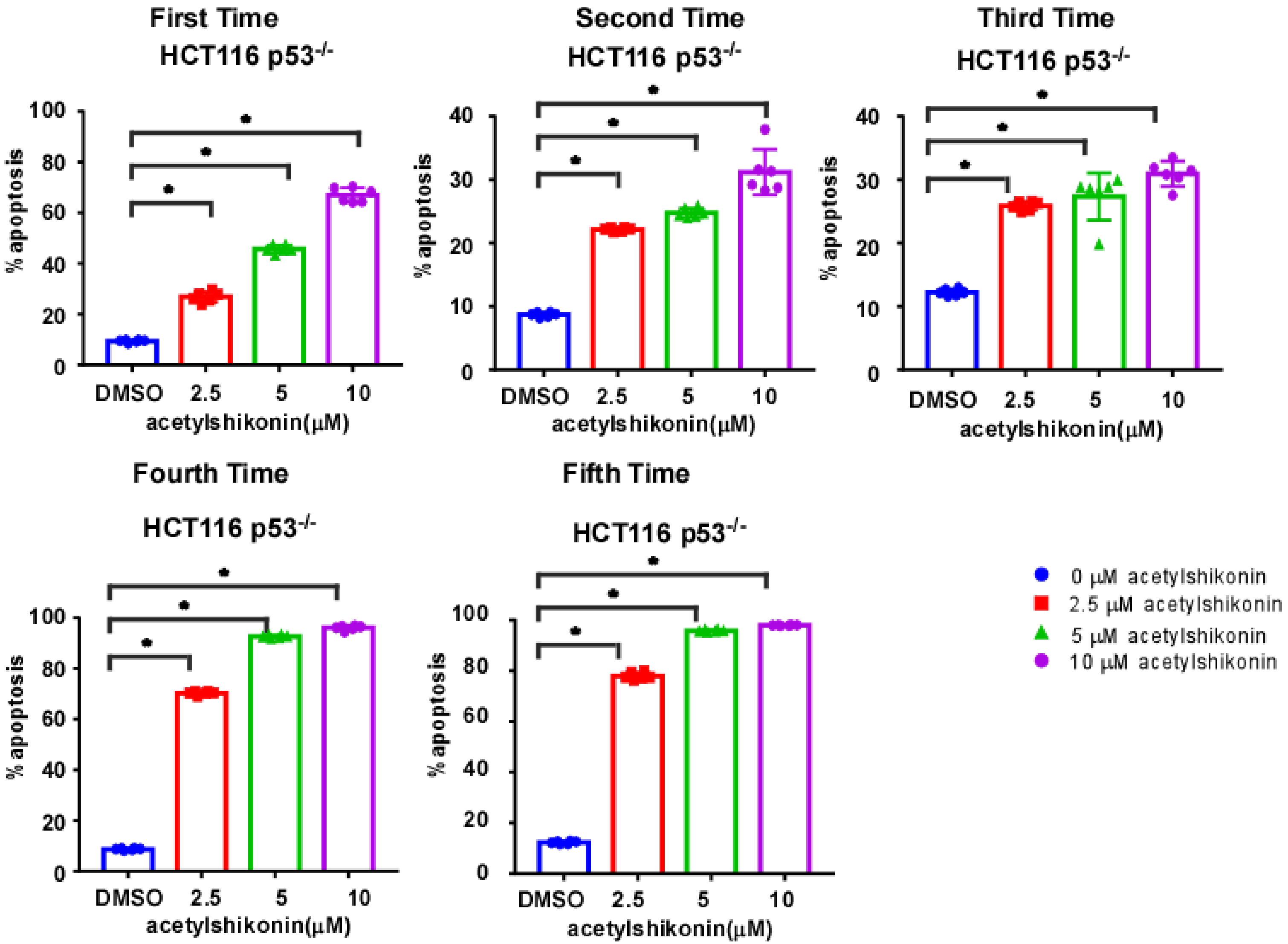

First Time
HCT116 p53-/-
Second Time
HCT116 p53-/-
Third Time
HCT116 p53-/-
Fourth Time
HCT116 p53-/-
Fifth Time
HCT116 p53-/-
% apoptosis
DMSO
2.5
5
10
acetylshikonin(μM)
0 μM acetylshikonin
2.5 μM acetylshikonin
5 μM acetylshikonin
10 μM acetylshikonin

Supplementary Figure 1

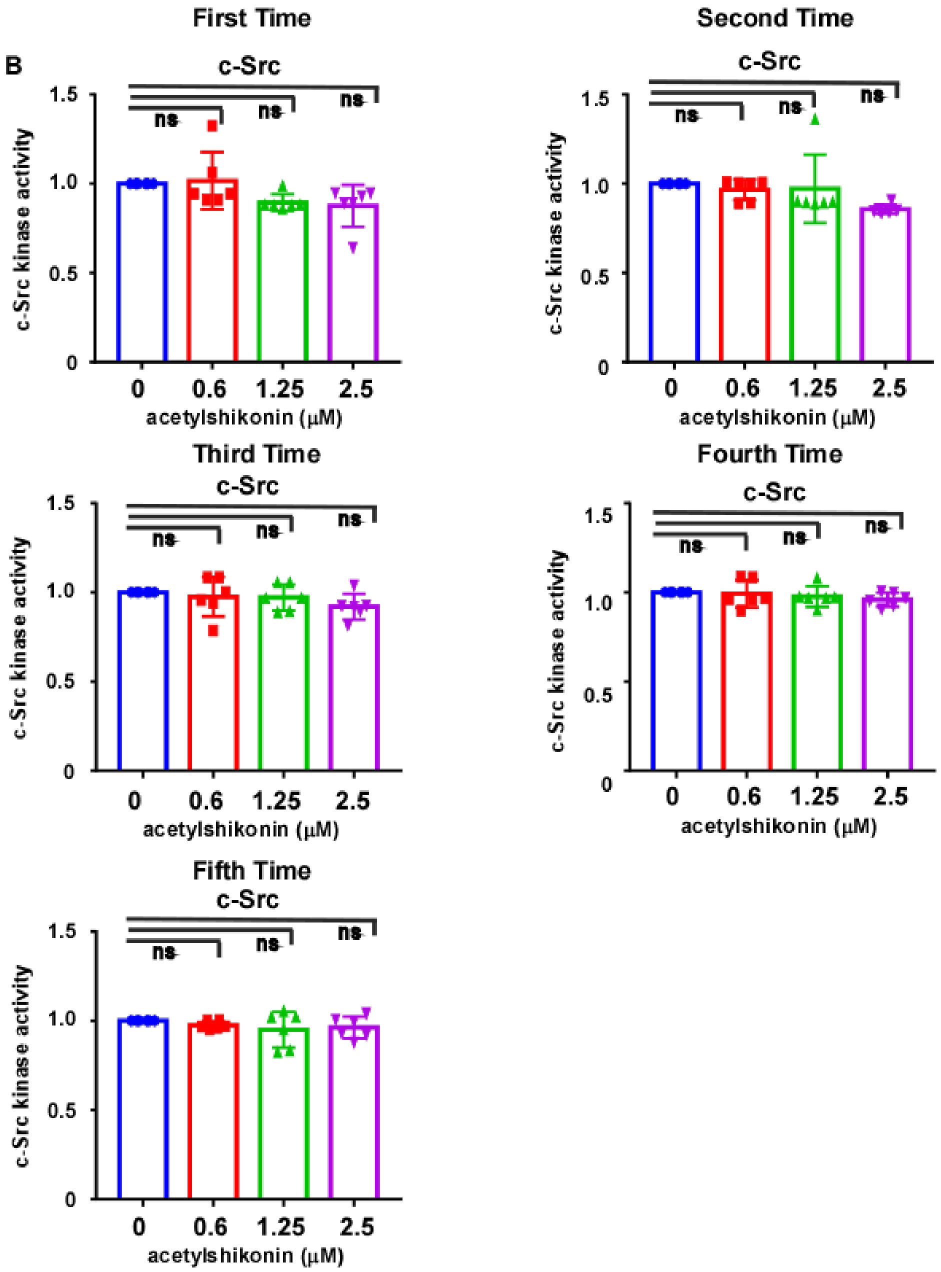

Supplementary Figure 2

A

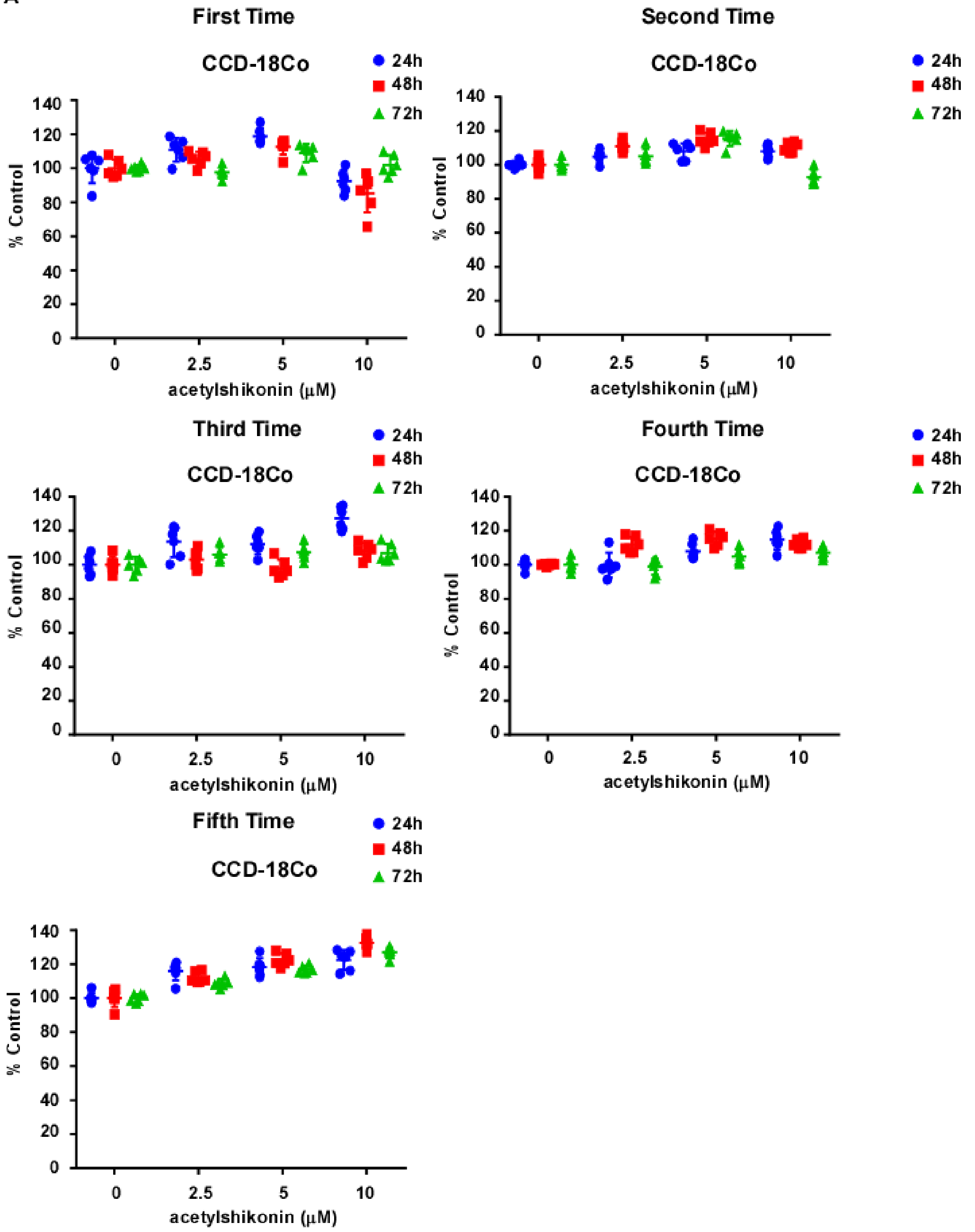

Supplementary Figure 2

C

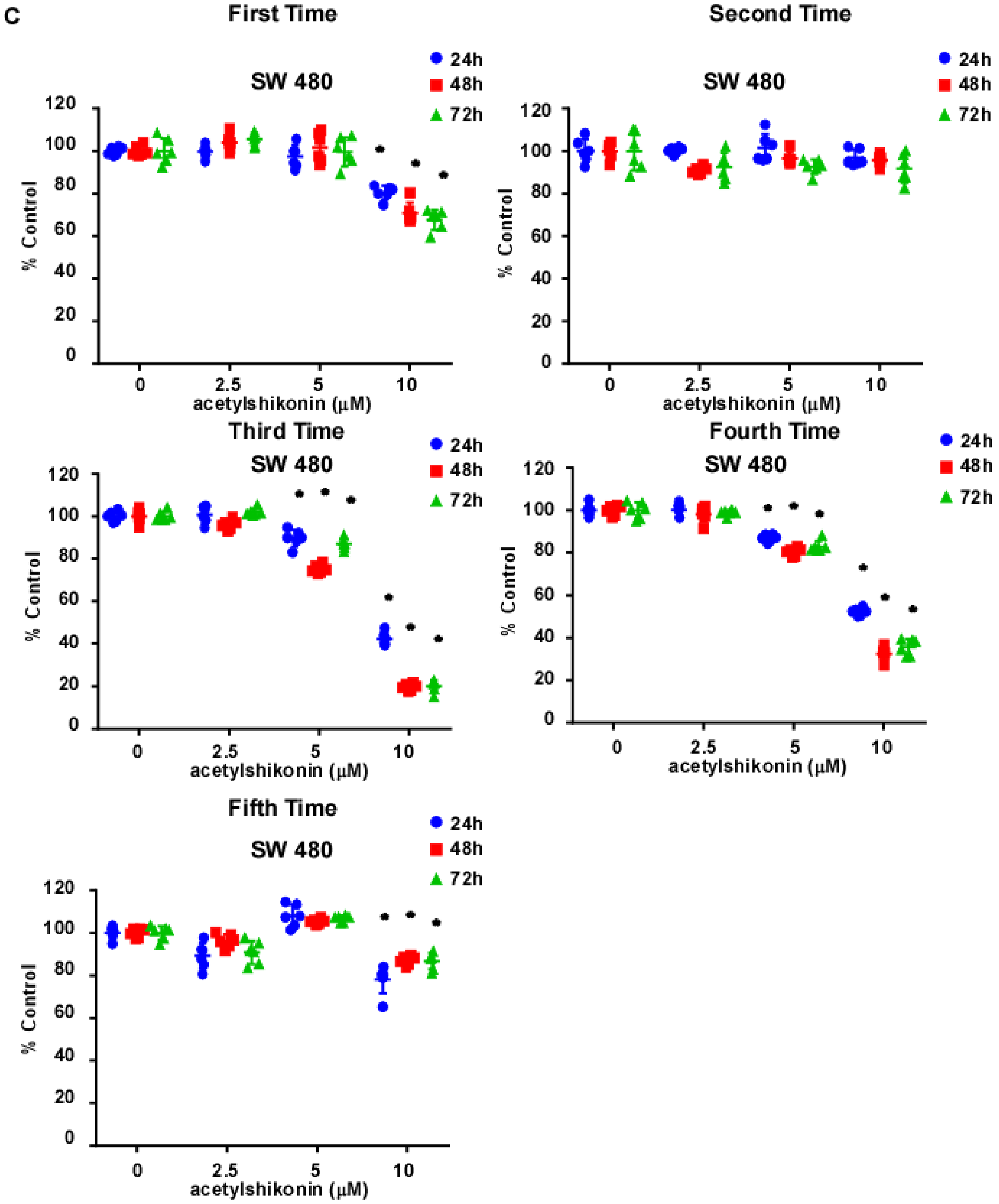

Supplementary Figure 2

C

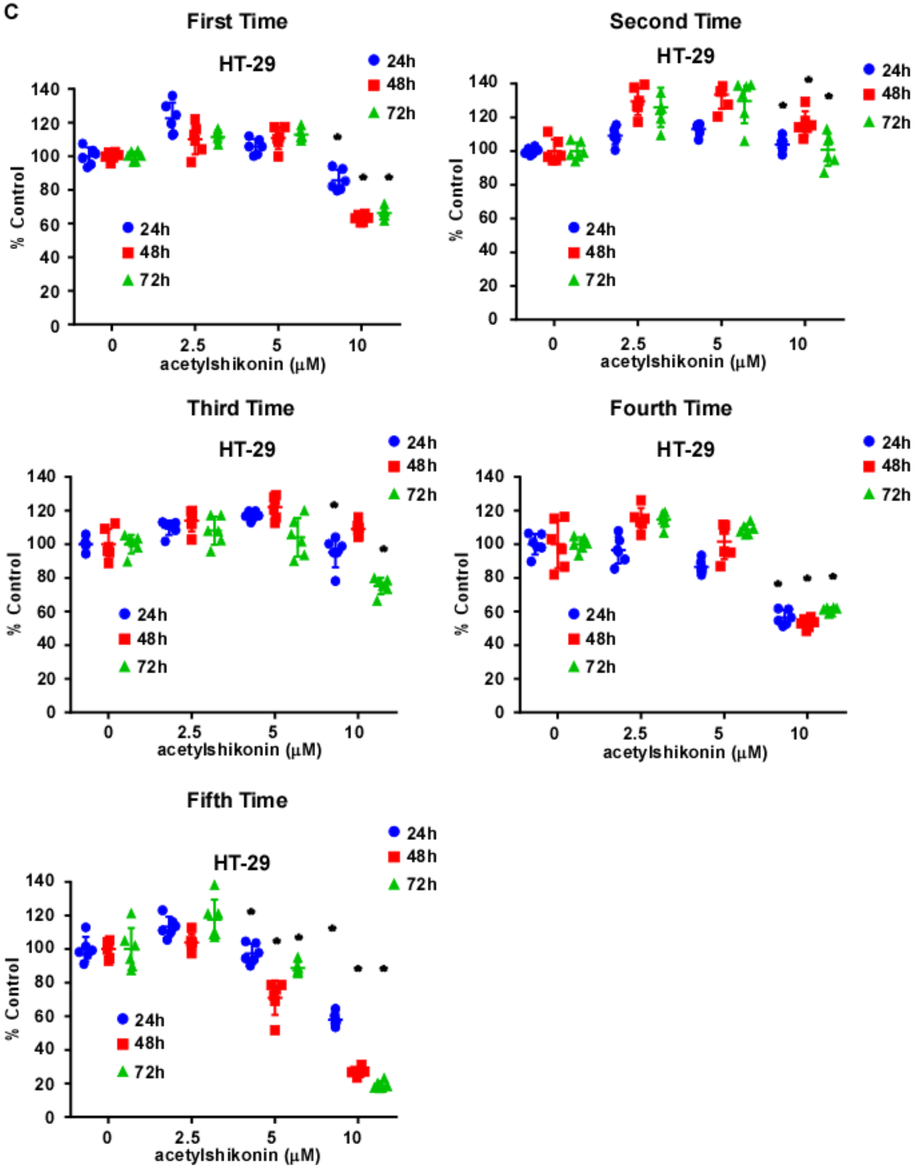

Supplementary Figure 3

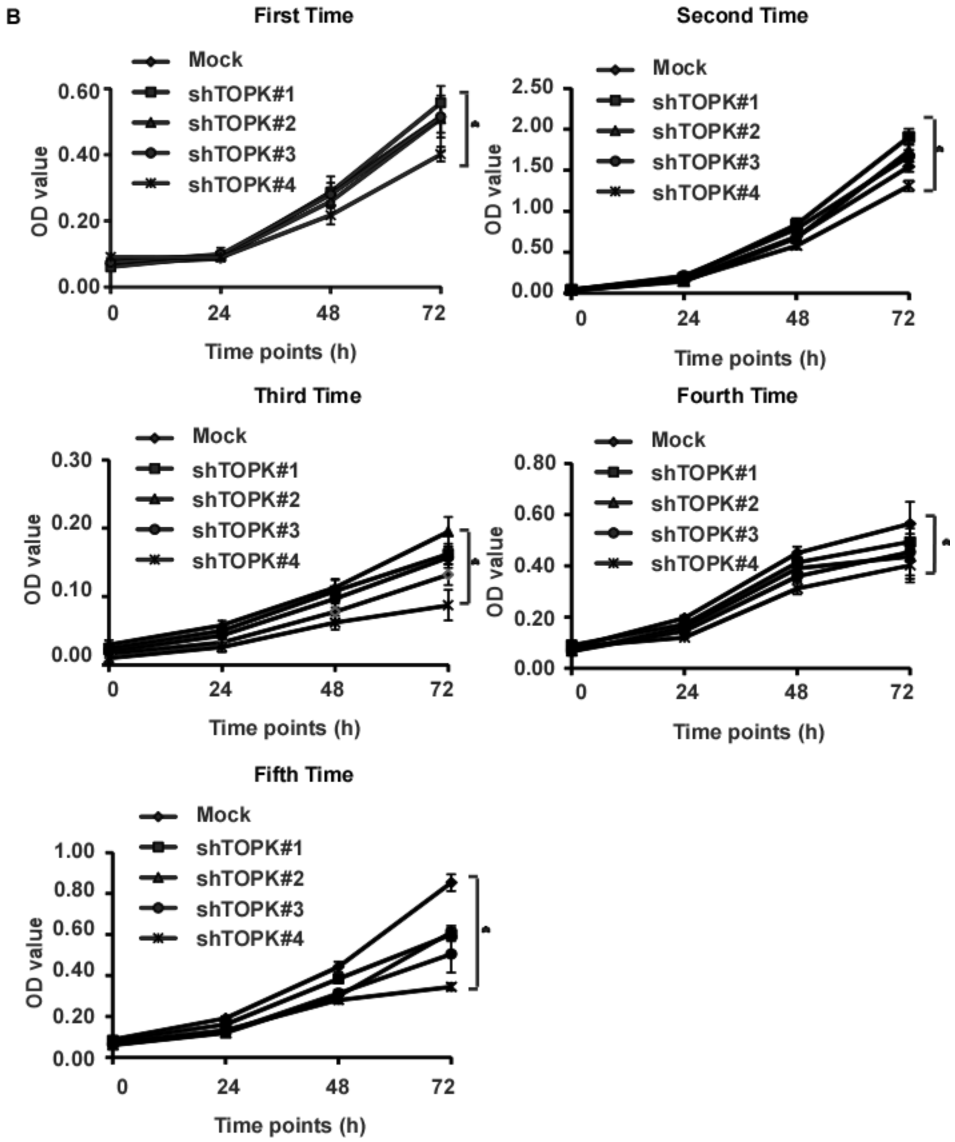

Supplementary Figure 3

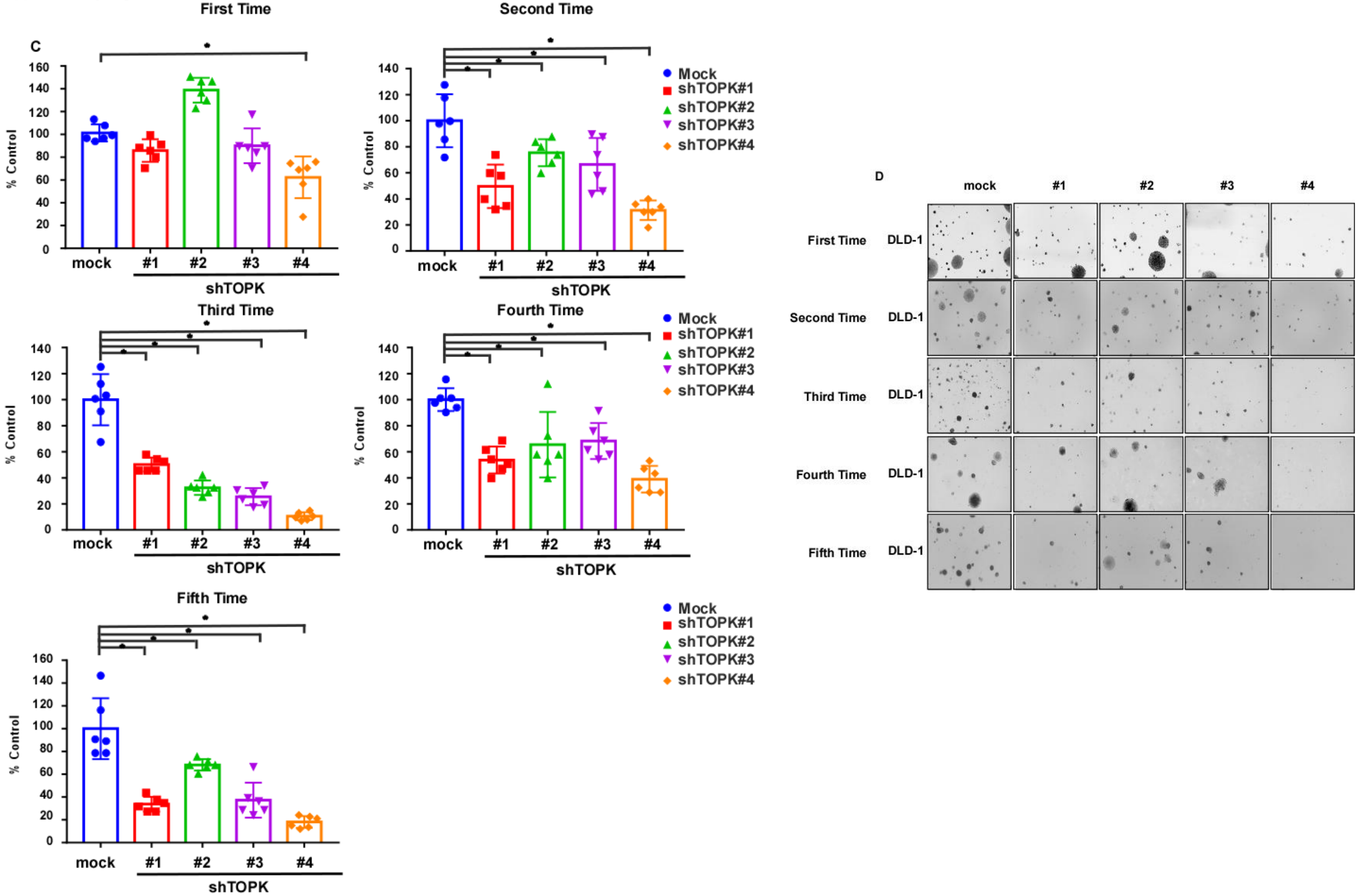

Supplement: Supplementary file 1 — Supporting info item [file BPH-177-2303-s001.pdf]
