# Supplementary material for: Acetylshikonin suppressed growth of colorectal tumour tissue and cells by inhibiting the intracellular kinase, T‐lymphokine‐activated killer cell‐originated protein kinase
Source: Br J Pharmacol. 2020 Apr 10;177(10):2303–19. doi: 10.1111/bph.14981 (PMC7174886; doi:10.1111/bph.14981)
Supplement: Supplementary file 2 — Supporting info item [file BPH-177-2303-s002.doc]

**Supplementary Figure Legends**

**Supplementary Figure 1.** The effect of acetylshikonin is assessed against several different kinases. (A) Kinase activity assay results of 7 different kinases indicate the effect of acetylshikonin at 20 μM. (B) *In vitro* kinase assay analysis the effect of acetylshikonin on Aurora A activity and densitometric quantification was evaluated by five independent experiments. Densitometric quantification data are shown as mean values ± S.D. The asterisks (* *p* < 0.05) indicate a significant inhibition of Aurora A activity treated acetylshikonin. (C) *In vitro* kinase assay analysis the effect of acetylshikonin on Aurora B activity and densitometric quantification was evaluated by five independent experiments. Densitometric quantification data are shown as mean values ± S.D. The asterisks (* *p* < 0.05) indicate a significant inhibition of Aurora B activity treated acetylshikonin and (D) *In vitro* kinase assay analysis the effect of acetylshikonin on c-Src activity was evaluated by five independent experiments. Data are shown as mean values ± S.D. The asterisks (**p* < 0.05) indicate a significant inhibition of c-Src activity treated acetylshikonin.

**Supplementary Figure 2.** Acetylshikonin suppresses growth of colon cancer cells by targeting TOPK. (A) Effects of acetylshikonin on normal CCD-18Co colon cells. Data are shown as means ±S.D. of five independent experiments. The asterisks (**p* < 0.05) indicate a significant difference between untreated control and acetylshikonin-treated cells. (B) The expression of TOPK signaling pathway in colon cancer cells was assessed by Western blot analysis and densitometric quantification was evaluated (number of independent experiment n=5). Densitometric quantification data are shown as mean values ± S.D. The asterisks (* *p* < 0.05) indicate a significant different expression of TOPK signaling pathway in colon cancer cell lines. (C) Treatment of SW 480 and HT-29 cells with acetylshikonin. Cells were treated with 0, 2.5, 5, or 10 μM acetylshikonin and proliferation was estimated by MTT assay at 24, 48, or 72 h (number of independent experiment n=5). Data are shown as mean values ±S.D. The asterisks (**p* < 0.05) indicate a significant difference between untreated control and acetylshikonin-treated cells.

**Supplementary Figure 3.** TOPK enhances proliferation of DLD-1 colon cancer cells. (A) The expression of TOPK in DLD-1 cells which was infected shRNA-mock or shRNA-TOPK #1-4 virus was evaluated by Western blotting and densitometric quantification was evaluated (number of independent experiment n=5). Densitometric quantification data are shown as mean values ± S.D. The asterisks (* *p* < 0.05) indicate a significant difference expression level of TOPK shRNA-mock and shRNA-TOPK-expressing cells. (B) The effect of acetylshikonin on growth of DLD-1 cells was estimated by MTS assay at 0, 24, 48, and 72 h (number of independent experiment n=5) Data are shown as means values ±S.D. (C) Anchorage-independent growth was assessed in DLD-1 cells expressing shRNA-mock or shRNA-TOPK (number of independent experiment n=5). Data are shown as means ±S.D. (D) Representative photos of anchorage-independent colonies. Data are shown as mean value ±S.D. The asterisks (* *p* < 0.05) indicate a significant difference between shRNA-mock and shRNA-TOPK-expressing cells, respectively.

**Supplementary Figure 4.** The expression of p53 in HCT 116 p53^+/+^ and HCT 116 p53^-/-^ cells. Cells were evaluated by Western blotting with a p53 antibody and densitometric quantification was evaluated (number of independent experiment n=5). Densitometric quantification data are shown as mean values ± S.D. The asterisks (* *p* < 0.05) indicate a significant difference expression level of p53 between HCT 116 p53^+/+^ and HCT 116 p53^-/-^ cells.

**Supplementary Figure 5.** The characteristics of patient tumor samples in the PDX mouse model. (A) Expression of TOPK in tumor samples used for the PDX mouse model and densitometric quantification was evaluated (number of independent experiment n=5). Densitometric quantification data are shown as mean values ± S.D. The asterisks (* *p* < 0.05) indicate a significant difference expression level of TOPK in the PDX mouse model. (B) Characteristics of patients (HJG41, HJG175, and HJG152) tumors were used in the PDX mouse model.

**Supplementary Figure 6.** Acetylshikonin attenuates the growth of PDX tumors (HJG175 and HJG152) in mice. (A, E) The effect of acetylshikonin on the volume of PDX tumors (HJG175 and HJG152) was plotted over 46 and 88 days, respectively. Vehicle or acetylshikonin (80 or 160 mg/kg for HJG175 and 60 or 120 mg/kg for HJG 152) were administered by oral gavage. Tumor volume was measured twice a week, n=10 in each group for the case of HJG175 and n=8 in each group for the case of HJG152. The asterisk (** p < 0.05*) indicates a significant decrease in volume of tumors from vehicle or acetylshikonin-treated mice. Data are shown as mean values ± S.D. (B, F) PDX tumor weight from mice treated with vehicle or acetylshikonin. (C, G) No changes in body weight were observed in mice treated with vehicle, or acetylshikonin. (D, H) White blood cell (WBC) count from mice treated with vehicle or acetylshikonin (80 or 160 mg/kg for HJG175 and 60 or 120 mg/kg for HJG 152).

**Supplementary Figure 7.** The expression of Ki-67, pTOPK, pERK, pRSK, or pcJun in HJG175 and HJG152 PDX tumors. (A) Representative photos of Ki-67, pTOPK, pERK, pRSK, or pcJun expression (B) Quantified graphs of the expression of Ki-67, pTOPK, pERK, pRSK, or pcJun. Each sample was quantified from 4 separate areas on each slide and an average of n=5 (vehicle and treatment) samples per group. Data are expressed as IOD values ±S.D. The asterisks (** p < 0.05*) indicate a significance difference between treated tissues compared to untreated controls.
