# Supplementary material for: Acetylshikonin suppressed growth of colorectal tumour tissue and cells by inhibiting the intracellular kinase, T‐lymphokine‐activated killer cell‐originated protein kinase
Source: Br J Pharmacol. 2020 Apr 10;177(10):2303–19. doi: 10.1111/bph.14981 (PMC7174886; doi:10.1111/bph.14981)

Supplementary Figure 1

A

Kinase	Inhibition%
Aurora A	52
Aurora B	64
c-Src	43
PDK1	15
AKT1	14
JNK1	-2
ERK1	6

B

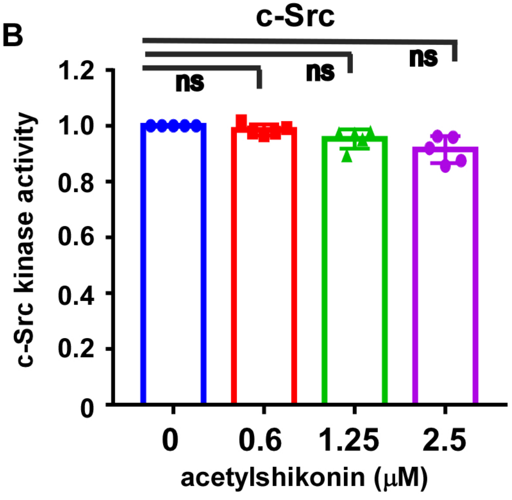

C

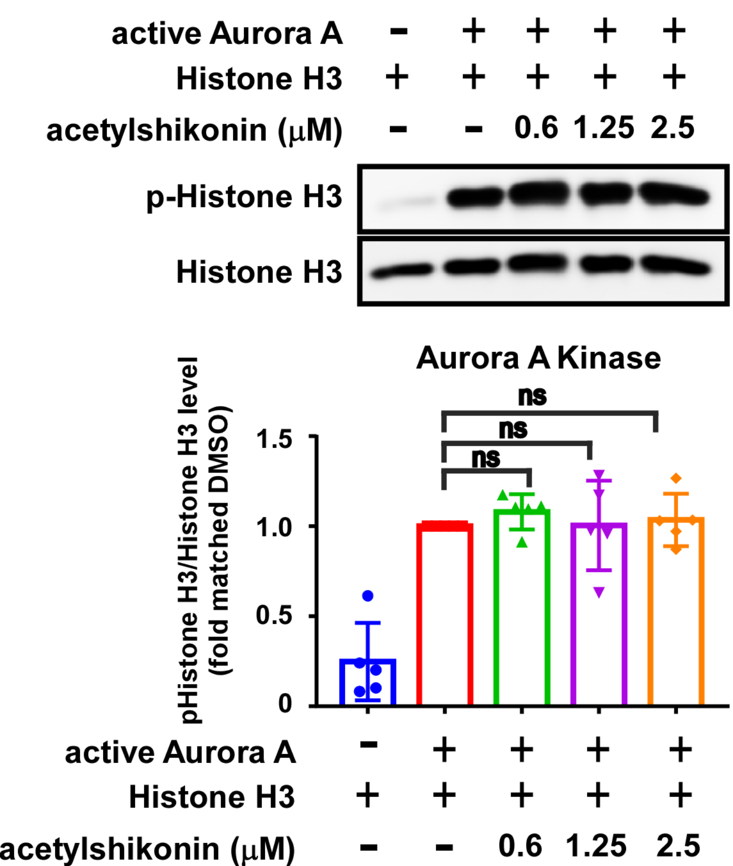

D

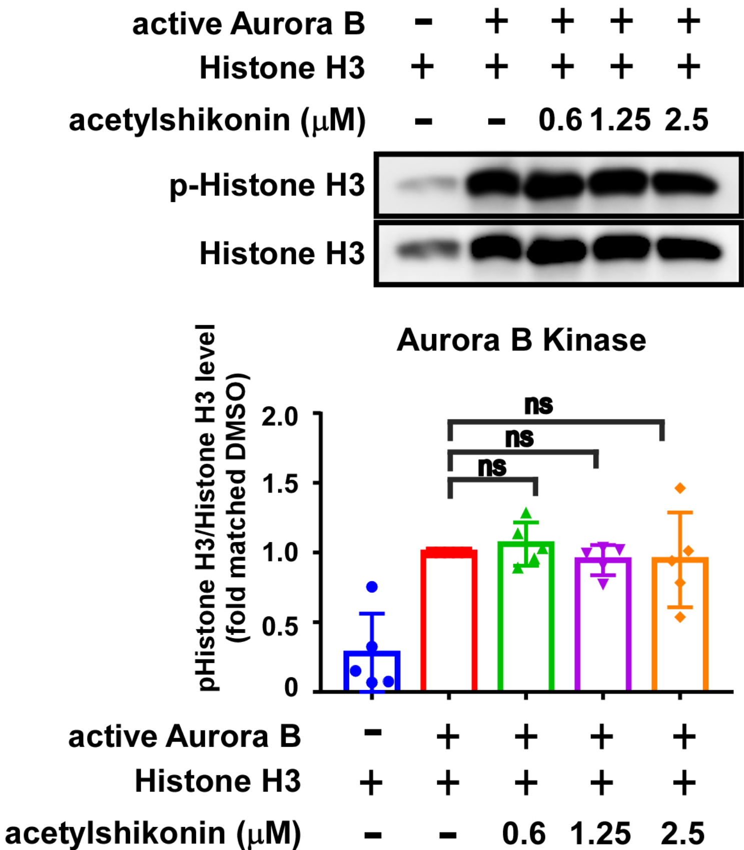

Supplement: Supplementary file 3 — Figure S1. The effect of acetylshikonin is assessed against several different kinases. (A) Kinase activity assay results of 7 different kinases indicate the effect of acetylshikonin at 20 μM. (B) in vitro kinase assay analysis the effect of acetylshikonin on Aurora A activity and densitometric quantification was evaluated by five independent experiments. Densitometric quantification data are shown as mean values ± S.D. The asterisks (* p < 0.05) indicate a significant inhibition of Aurora A activity treated acetylshikonin. (C) in vitro kinase assay analysis the effect of acetylshikonin on Aurora B activity and densitometric quantification was evaluated by five independent experiments. Densitometric quantification data are shown as mean values ± S.D. The asterisks (* p < 0.05) indicate a significant inhibition of Aurora B activity treated acetylshikonin and (D) in vitro kinase assay analysis the effect of acetylshikonin on c‐Src activity was evaluated by five independent experiments. Data are shown as mean values ± S.D. The asterisks (*p < 0.05) indicate a significant inhibition of c‐Src activity treated acetylshikonin. [file BPH-177-2303-s003.pdf]
