# Supplementary figures and images for: Acetylshikonin suppressed growth of colorectal tumour tissue and cells by inhibiting the intracellular kinase, T‐lymphokine‐activated killer cell‐originated protein kinase
Source: Br J Pharmacol. 2020 Apr 10;177(10):2303–19. doi: 10.1111/bph.14981 (PMC7174886; doi:10.1111/bph.14981)

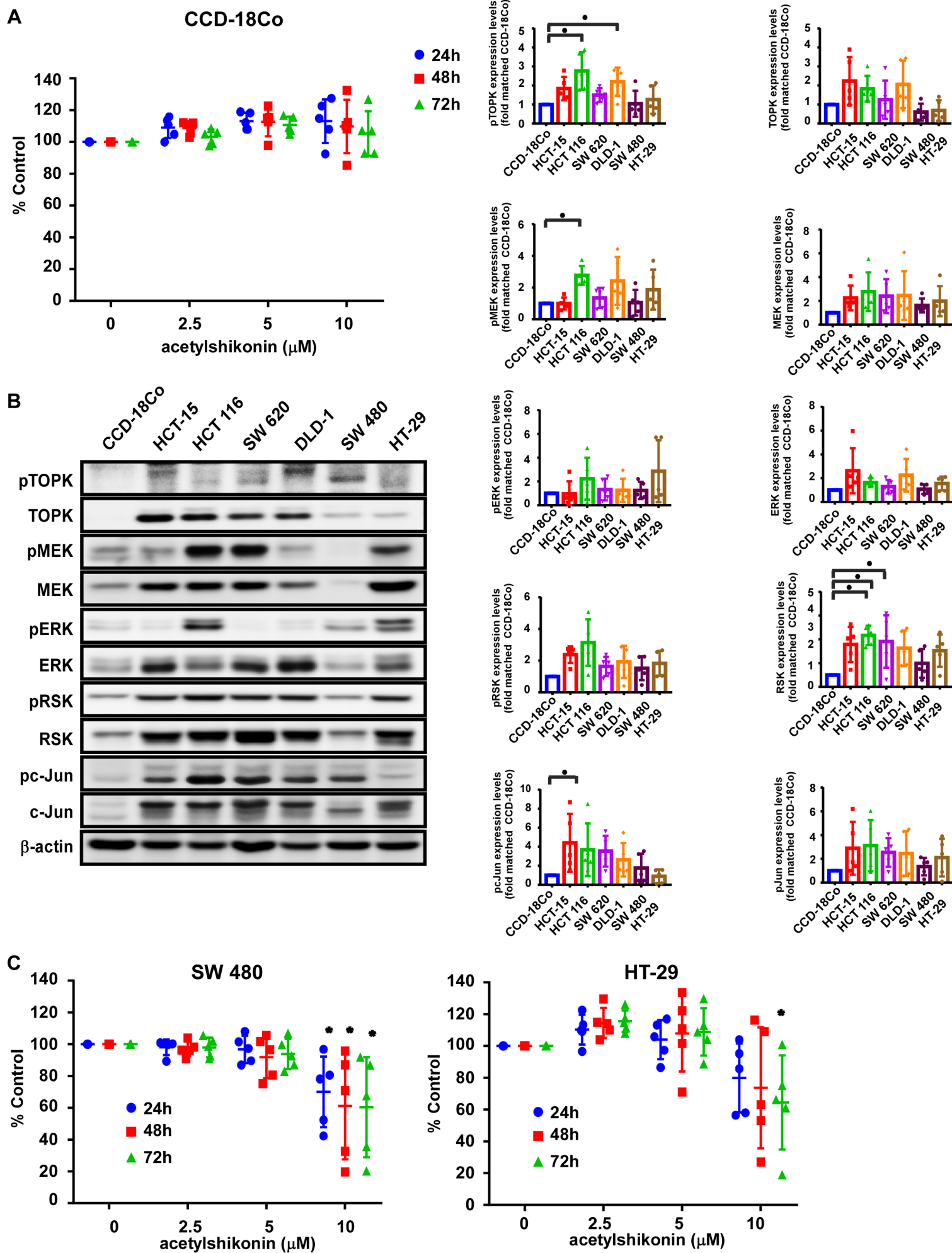

Supplement: Supplementary file 4 — Figure S2. Acetylshikonin suppresses growth of colon cancer cells by targeting TOPK. (A) Effects of acetylshikonin on normal CCD‐18Co colon cells. Data are shown as means ±S.D. of five independent experiments. The asterisks (*p < 0.05) indicate a significant difference between untreated control and acetylshikonin‐treated cells. (B) The expression of TOPK signaling pathway in colon cancer cells was assessed by Western blot analysis and densitometric quantification was evaluated (number of independent experiment n=5). Densitometric quantification data are shown as mean values ± S.D. The asterisks (* p < 0.05) indicate a significant different expression of TOPK signalling pathway in colon cancer cell lines. (C) Treatment of SW 480 and HT‐29 cells with acetylshikonin. Cells were treated with 0, 2.5, 5, or 10 μM acetylshikonin and proliferation was estimated by MTT assay at 24, 48, or 72 h (number of independent experiment n=5). Data are shown as mean values ±S.D. The asterisks (*p < 0.05) indicate a significant difference between untreated control and acetylshikonin‐treated cells. [file BPH-177-2303-s004.pdf]

Supplementary Figure 3

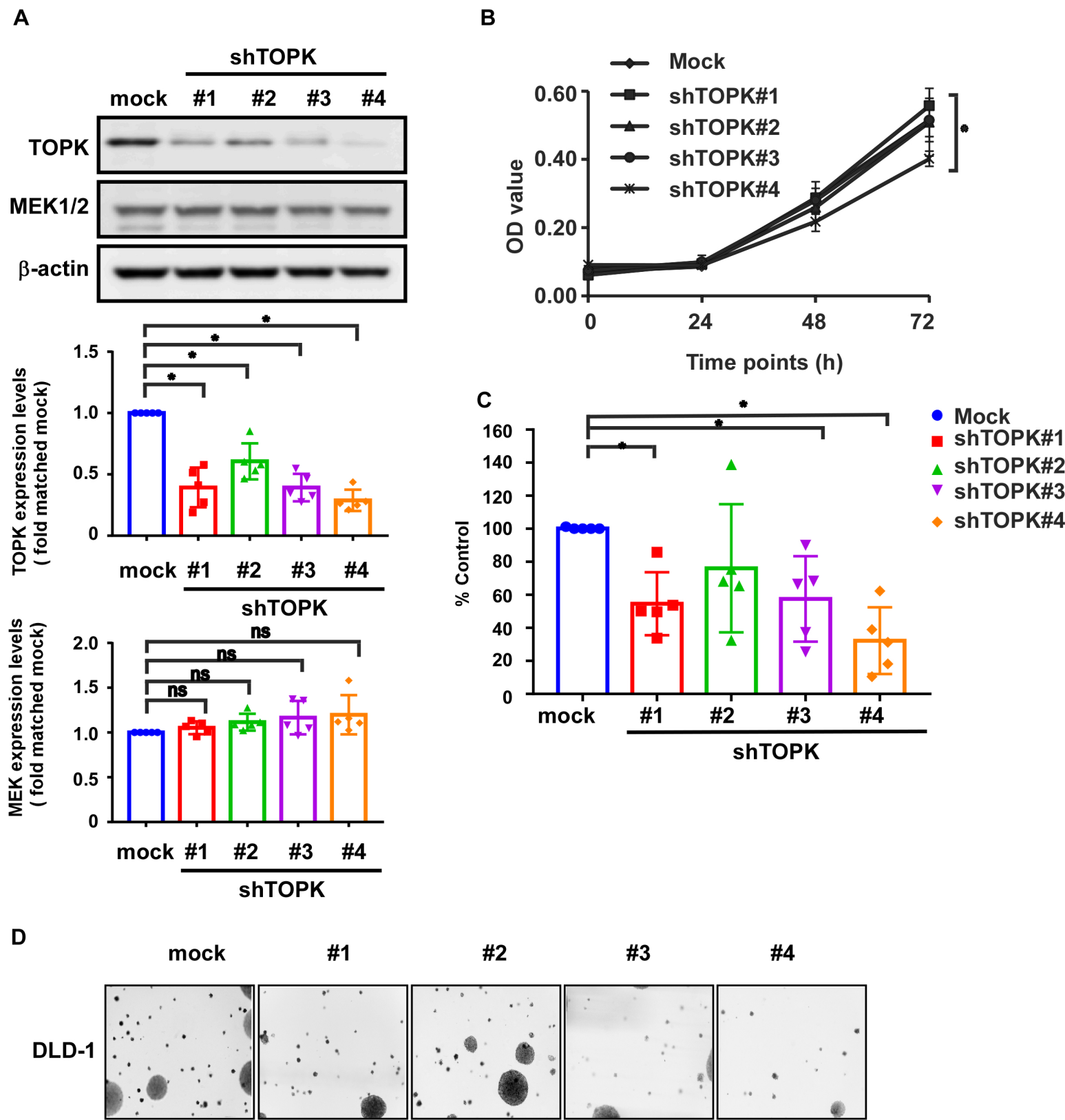

Supplement: Supplementary file 5 — Figure S3. TOPK enhances proliferation of DLD‐1 colon cancer cells. (A) The expression of TOPK in DLD‐1 cells which was infected shRNA‐mock or shRNA‐TOPK #1‐4 virus was evaluated by Western blotting and densitometric quantification was evaluated (number of independent experiment n=5). Densitometric quantification data are shown as mean values ± S.D. The asterisks (* p < 0.05) indicate a significant difference expression level of TOPK shRNA‐mock and shRNA‐TOPK‐expressing cells. (B) The effect of acetylshikonin on growth of DLD‐1 cells was estimated by MTS assay at 0, 24, 48, and 72 h (number of independent experiment n=5) Data are shown as means values ±S.D. (C) Anchorage‐independent growth was assessed in DLD‐1 cells expressing shRNA‐mock or shRNA‐TOPK (number of independent experiment n=5). Data are shown as means ±S.D. (D) Representative photos of anchorage‐independent colonies. Data are shown as mean value ±S.D. The asterisks (* p < 0.05) indicate a significant difference between shRNA‐mock and shRNA‐TOPK‐expressing cells, respectively. [file BPH-177-2303-s005.pdf]

Supplementary Figure 4

A

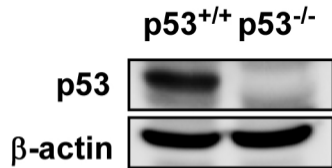

p53 expression levels  
(fold matched  $p53^{+/+}$  cell line)

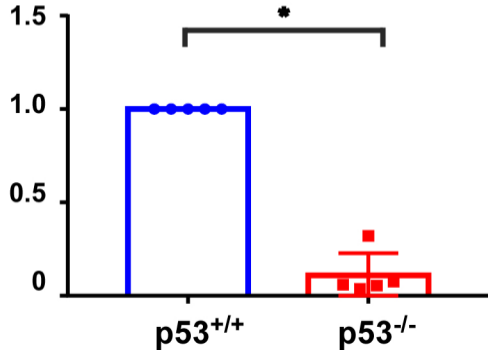

Supplement: Supplementary file 6 — Figure S4. The expression of p53 in HCT 116 p53+/+ and HCT 116 p53‐/‐ cells. Cells were evaluated by Western blotting with a p53 antibody and densitometric quantification was evaluated (number of independent experiment n=5). Densitometric quantification data are shown as mean values ± S.D. The asterisks (* p < 0.05) indicate a significant difference expression level of p53 between HCT 116 p53+/+ and HCT 116 p53‐/‐ cells. [file BPH-177-2303-s006.pdf]

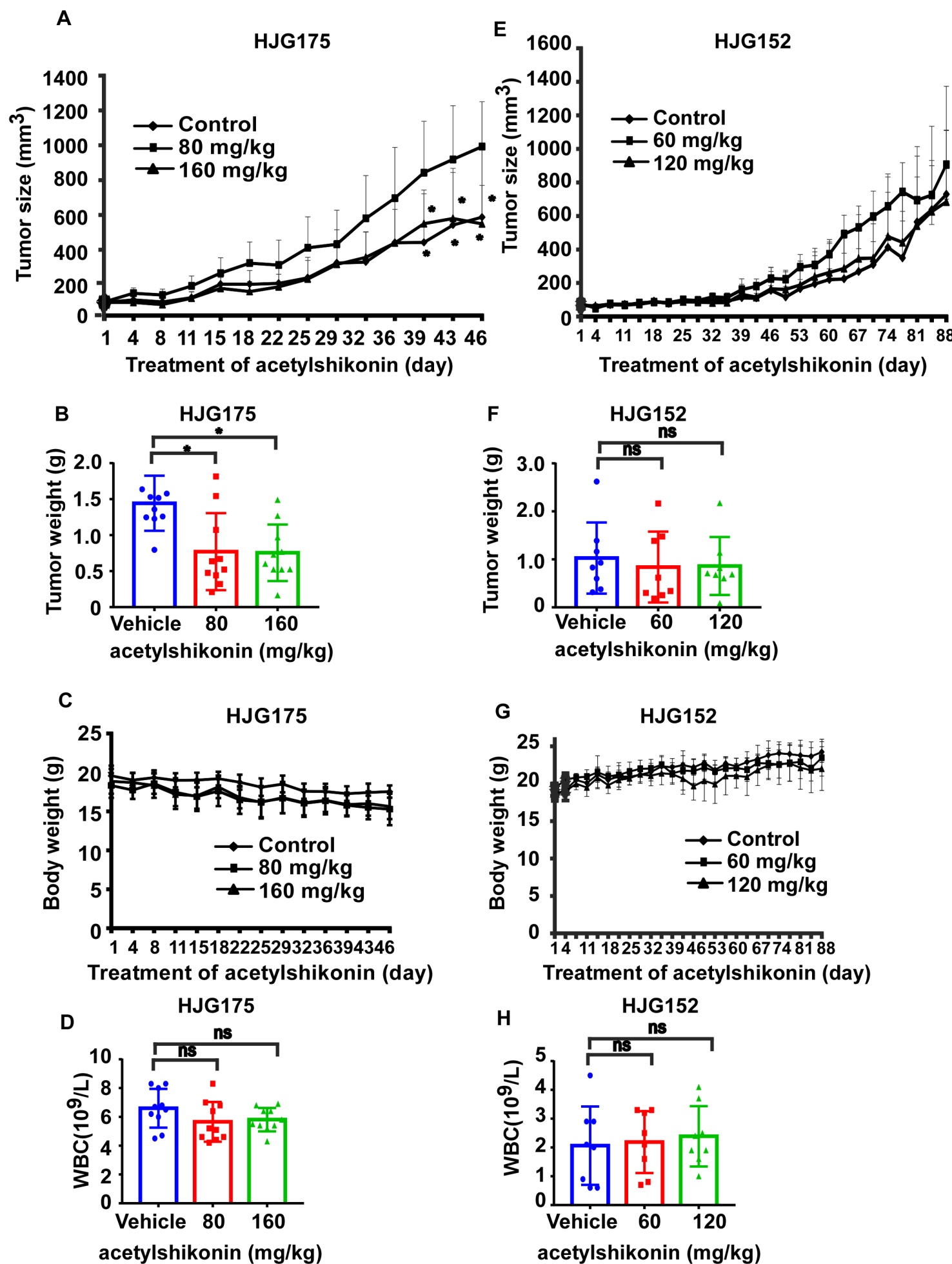

Supplement: Supplementary file 8 — Figure S6. Acetylshikonin attenuates the growth of PDX tumors (HJG175 and HJG152) in mice. (A, E) The effect of acetylshikonin on the volume of PDX tumors (HJG175 and HJG152) was plotted over 46 and 88 days, respectively. Vehicle or acetylshikonin (80 or 160 mg/kg for HJG175 and 60 or 120 mg/kg for HJG 152) were administered by oral gavage. Tumor volume was measured twice a week, n=10 in each group for the case of HJG175 and n=8 in each group for the case of HJG152. The asterisk (* p < 0.05) indicates a significant decrease in volume of tumors from vehicle or acetylshikonin‐treated mice. Data are shown as mean values ± S.D. (B, F) PDX tumor weight from mice treated with vehicle or acetylshikonin. (C, G) No changes in body weight were observed in mice treated with vehicle, or acetylshikonin. (D, H) White blood cell (WBC) count from mice treated with vehicle or acetylshikonin (80 or 160 mg/kg for HJG175 and 60 or 120 mg/kg for HJG 152). [file BPH-177-2303-s008.pdf]

Supplementary Figure 7

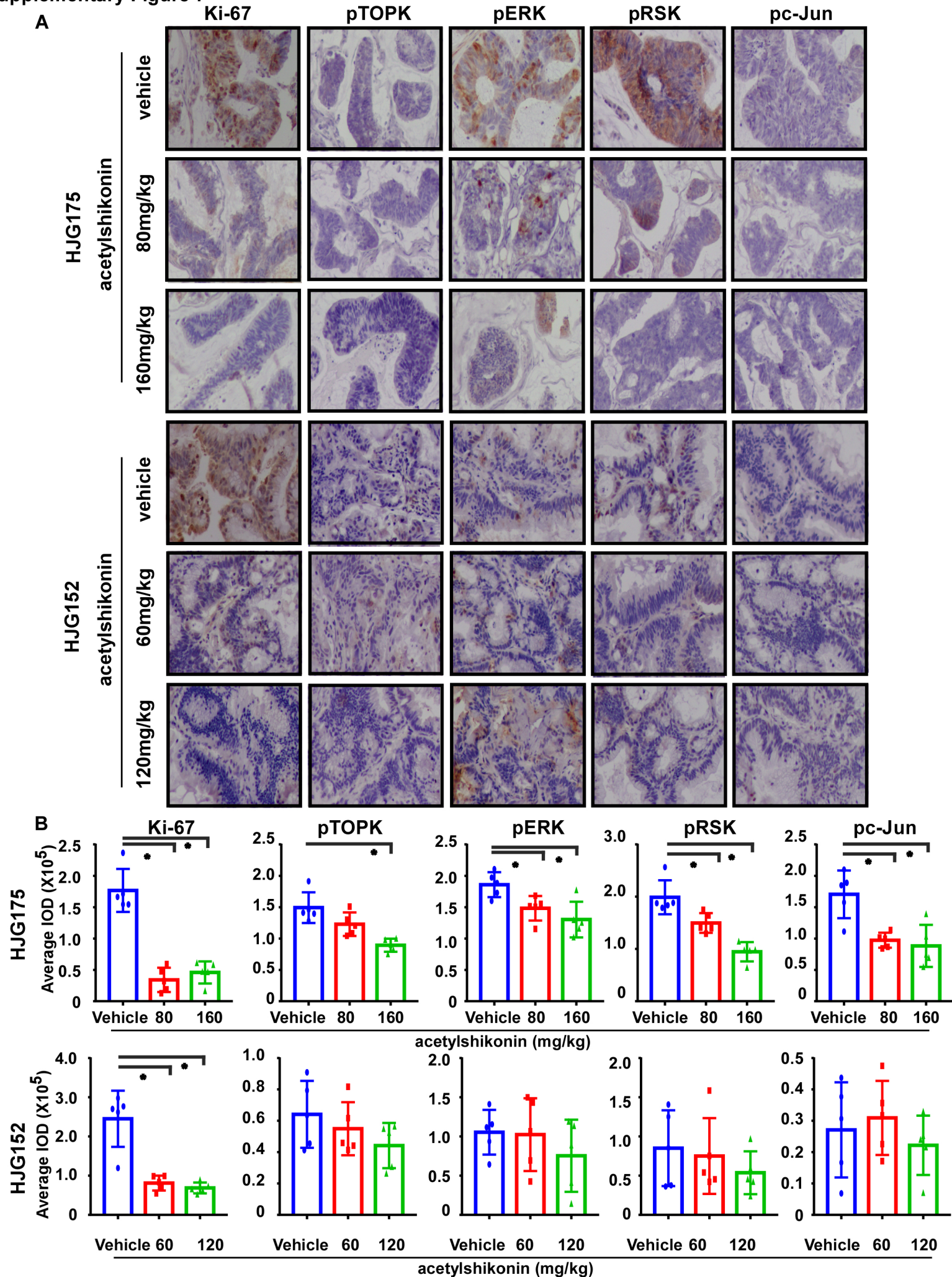

Supplement: Supplementary file 9 — Figure S7. The expression of Ki‐67, pTOPK, pERK, pRSK, or pc‐Jun in HJG175 and HJG152 PDX tumors. (A) Representative photos of Ki‐67, pTOPK, pERK, pRSK, or pcJun expression (B) Quantified graphs of the expression of Ki‐67, pTOPK, pERK, pRSK, or pc‐Jun. Each sample was quantified from 4 separate areas on each slide and an average of n=5 (vehicle and treatment) samples per group. Data are expressed as IOD values ±S.D. The asterisks (* p < 0.05) indicate a significance difference between treated tissues compared to untreated controls. [file BPH-177-2303-s009.pdf]
