# Supplementary material for: Acetylshikonin suppressed growth of colorectal tumour tissue and cells by inhibiting the intracellular kinase, T‐lymphokine‐activated killer cell‐originated protein kinase
Source: Br J Pharmacol. 2020 Apr 10;177(10):2303–19. doi: 10.1111/bph.14981 (PMC7174886; doi:10.1111/bph.14981)
Supplement: Supplementary file 7 — Figure S5. The characteristics of patient tumor samples in the PDX mouse model. (A) Expression of TOPK in tumor samples used for the PDX mouse model and densitometric quantification was evaluated (number of independent experiment n=5). Densitometric quantification data are shown as mean values ± S.D. The asterisks (* p < 0.05) indicate a significant difference expression level of TOPK in the PDX mouse model. (B) Characteristics of patients (HJG41, HJG175, and HJG152) tumors were used in the PDX mouse model. [file BPH-177-2303-s007.pdf]

Supplementary Figure 5

A

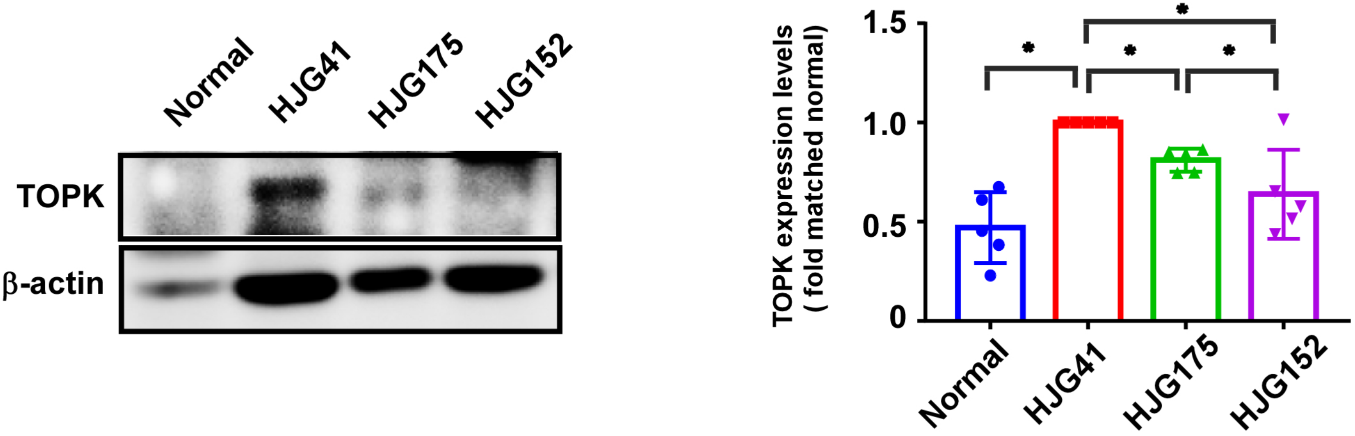

B

Model ID	Gender	Age(yrs)	Source	Histology	TNM grade	Cancer stage
HJG41	Male	46	Primary	Retcal cacner	IIIB	T3N2M0
HJG175	Male	66	Primary	Colon cancer	IIA	T3N0M0
HJG152	Female	75	Primary	Retcal cacner	IIIA	T2N1M0
